# Combining ability and heterosis analysis for mineral content in the leafy vegetable *Gynandropsis gynandra* (L.) Briq.

**DOI:** 10.1371/journal.pone.0332095

**Published:** 2025-09-12

**Authors:** Aristide Carlos Houdegbe, Enoch G. Achigan-Dako, E. O. Dêêdi Sogbohossou, Alfred O. Odindo, M. Eric Schranz, Julia Sibiya

**Affiliations:** 1 Genetics, Biotechnology and Seed Science Unit (GBioS), Laboratory of Crop Production, Physiology and Plant Breeding, Faculty of Agricultural Sciences, University of Abomey-Calavi, Abomey-Calavi, Republic of Benin; 2 School of Agriculture and Science, University of KwaZulu-Natal, Pietermaritzburg, Republic of South Africa; 3 Biosystematics Group, Wageningen University, Wageningen, The Netherlands; 4 Sustainable Agrifood Systems Program (SAS), International Maize and Wheat Improvement Center (CIMMYT), Nairobi, Kenya; KGUT: Graduate University of Advanced Technology, IRAN, ISLAMIC REPUBLIC OF

## Abstract

Spider plant (*Gynandropsis gynandra*) is a leafy vegetable rich in micronutrients, including minerals, vitamins, and secondary metabolites, making it a valuable opportunity crop for combating hidden hunger and promoting human health. However, knowledge of the inheritance of mineral content is limited, which hinders the development of improved cultivars for wider cultivation. To address this, 118 F_1_ experimental hybrids involving 26 parental lines were generated from a North Carolina mating design II. The F_1_s and their parents were evaluated across two years (2019 and 2020) for gene action, combining ability effects and heterosis of leaf mineral (zinc, copper, manganese, calcium, magnesium, sodium, phosphorus, and potassium) content. Significant differences (p < 0.001) were observed among and between hybrids and parents for iron, zinc, copper, manganese, calcium, magnesium, sodium, phosphorus, and potassium contents. The genotype × year interaction was also significant, with variance greater than the genotypic variance. Significant general and specific combining ability effects, together with variance components analysis, revealed that both additive and nonadditive gene action controlled mineral content, with a predominance of nonadditive gene action. Mid- and best-parent heterosis ranged from -80.4% to 389.5% for mineral content. Parents with good general combining ability were identified, as well as crosses with high specific combining ability and heterosis. There were significant and moderate to strong correlations between mean hybrid performance, specific combining ability effects, and heterosis levels, and low to moderate correlations between general combining ability and the performance of the mean parents. We conclude that hybridization in *G. gynandra* contributes to improving the mineral content. *G. gynandra* can be used as a model crop to study the genetic mechanism underlying heterosis in leafy vegetables.

## Introduction

The nutritive value of vegetables, particularly orphan, underutilized, or opportunity vegetables, is high, and they are an essential source of micronutrients. The increasing interest in leafy vegetables is particularly due to their distinct richness in minerals, vitamins, phytochemicals, and antioxidants [1–3], as well as their good adaptation to local conditions, representing a reliable resource for adapting to a changing climate. The nutritive richness makes leafy vegetables valuable crops to diversify the world’s food portfolio, which, to date, is limited to a few crops [4]. Ongoing actions to promote opportunity crops include the development of genomic tools to speed up new cultivar development to meet consumers’ preferences [5], as well as raising population awareness of their potential value and the Vision for Adapted Crops and Soils (VACS) [6]. In the case of leafy vegetables, the main breeding objective is to develop high leaf-yielding cultivars [7], to optimize resource use and availability. This objective might conflict or align with the primary potential of leafy vegetables, which is their high micronutrient content. Marles [8] provided evidence of a decline in mineral content in crops, including vegetables. For example, a drop of 10% to 52% of minerals content in fruits and vegetables was observed in the United Kingdom from 1940 to 2019 [9]. The decrease in mineral content is referred to as the “dilution effect”, which is an increase in yield in modern cultivars without a subsequent increase in mineral content or higher mineral nutrient concentrations in old cultivars than in improved cultivars. The decline was observed to be more pronounced in vegetables than in other commodity groups [10]. Therefore, assessing the nutritional value of the breeding material throughout the breeding process is essential for the conservation of the primary benefits of leafy vegetables while increasing their productivity.

*Gynandropsis gynandra* (L.) Briq. (syn. *Cleome gynandra* L.), the commonly known spider plant is interesting because of its high content of vitamins, minerals, and secondary metabolites [2,11–17]. The major vitamins reported in the species include vitamins C, A, and E, and minerals include microelements (iron, zinc, copper, manganese) and macrominerals (calcium, potassium, magnesium, phosphorus, and sodium) [11,12,17]. Minerals and vitamins, referred to as micronutrients, are essential in very small quantities and play a crucial role in maintaining health. Although all micronutrients are necessary for human health and bodily function, potassium, calcium, iron, zinc, and magnesium are of global health concern as their deficiencies affect billions of people worldwide [18]. The consumption of spider plant leaves could provide 10–100% of the recommended dietary allowance for human body needs, depending on the age group and the mineral [17]. The species also contains several secondary metabolites, including flavonoids, terpenoids, tannins, glucosinolates, and various phenolic compounds, which are essential for human health [2,12,13,19,20]. All these nutritional and health-promoting compounds exhibit a wide range of variation in the species, providing a strong basis for crop improvement [11,12,16,17,19].

More importantly, *G. gynandra* leaves have 4.7- and 3.2-fold of vitamin C, 4- and 2-fold iron, 1.2- and 2.1-fold zinc, 2.7- and 10.4-fold calcium, 3.3- and 5.5-fold phosphorus, 1.4- and 1.8-fold potassium, 2.59- and 1.37-fold total phenolics, and 5.70- and 2.46-fold flavonoids concentrations higher than two world leading commercial and consumed vegetables namely cabbage (*Brassica oleracea* var. *capitata* cv. Drumhead) and Swiss chard (*Beta vulgaris* L. cv. Fordhook Giant), respectively [2]. Breeding efforts in *G. gynandra* will result in significant impacts on healthier and balanced diets for local communities in Africa and Asia where the species is mostly consumed [7,21] and high micronutrient deficiencies occurs. Moreover, *G. gynandra* is a climate resilient crop as it is a C_4_ plant with the ability to withstand various harsh conditions [21]; the species was used as a model crop to understand C_4_ traits, evolution and gene expression in plants [22–24]. The production and the exploitation of the nutritional potential of *G. gynandra* like many other opportunity crops are limited due to lack of improved varieties resulting from the lack of sustainable breeding program.

Breeding strategies and type of cultivars for a given crop species are guided by the species reproductive biology and mating systems. Both self- and cross-compatibility occurs in *G. gynandra* with outcrossing being predominant [25–27], offering the possibility for developing both hybrids and inbred cultivars. Because of the predominance of outcrossing, the species might exhibit heterosis and the choice for developing hybrids cultivars sounds as good strategy to exploit the potential heterosis that needs to be properly documented. Heterosis or hybrid vigour refers to the outperformance of the first generation of progenies compared to their parents [28–31] and has been highly researched by breeders for open-pollinated crops but also for self-pollinated crops for higher productivity. Four steps are crucial in the development of a hybrid cultivar: (i) the establishment of populations for selection; (ii) inbred lines development; (iii) inbred lines’ evaluation for combining ability; and (iv) the production of hybrid seed [32]. Following the development of the inbred/advanced lines in *G. gynandra* [17,33], their testing for combining ability is the next step towards hybrids creation. Combining ability refers to the ability of a line to combine with another one during hybridization so that desirable genes or traits are transferred to their progenies and encompasses two types, namely the general combining ability (GCA) and the specific combining ability (SCA) [34]. According to Sprague and Tatum [34], a GCA of a line is the average performance of this line in a set of its hybrid combinations and the SCA is the deviation of a specific cross performance from the sum of the average performance or GCA of its parental lines. Combining ability informs on the nature and magnitude of gene action controlling the considered trait. GCA are associated with additive genes effects, while the SCA effects are attributed to nonadditive gene action, including dominance and epistasis gene effects [34]. The GCA helps in the selection of good parents for breeding and the SCA is useful for the selection of the best hybrid combination to better exploit heterosis. The assessment of the combining ability of a set of lines and the inheritance patterns for a target species is done using mating designs such as North Carolina Design II, diallel and line by tester, among others [35–37].

*G. gynandra* belongs to Cleomaceae family, a sister family of Brassicaceae and could gain more from breeding strategies implemented in *Brassica* crops. Most developed varieties for *Brassica* crops nowadays are hybrids and the evaluation of combining ability, heterosis level and gene action has been intensively done for several parents in *Brassica oleracea* var. *capitata* [38], *Brassica rapa* L. [39], and *Brassica oleracea* var. *botrytis* L. [40,41] for nutritional traits such as minerals, antioxidant pigments, and vitamins. Unfortunately, to the best of our knowledge, the heterosis level and the combining ability potential for mineral content in *G. gynandra* are yet to be investigated to inform breeding program of good parents, heterotic crosses and breeding methods for improved cultivars development.

In this study, we generated knowledge on the genetic mechanism controlling mineral content in *G. gynandra* with a good perspective for hybrid cultivar development. The present study aimed to investigate the mineral profile of experimental hybrids of spider plant to select high-nutrient hybrids for cultivar development. Specifically, the study (i) compared the mineral content of 118 experimental F_1_ *G. gynandra* hybrids and their parental lines; (ii) quantified heterosis magnitude for leaf mineral content in *G. gynandra* to select the most suitable breeding method; (iii) evaluated the combining ability effects for leaf mineral content of the parental lines of *G. gynandra* to identify good parents and best crosses; and (iv) estimated the extent of association between mean performance, heterosis and combining ability for mineral content in *G. gynandra* to guide prediction of hybrid performance and multiple traits selection. The following research questions guided this study: (i) How does the leaf mineral content in *G. gynandra* pass from the parents to the progenies? (ii) Does heterosis exist for leaf mineral content in *G. gynandra*, and to what extent? (iii) What could be the breeding strategies for the development of nutrient-dense cultivars of spider plant? We therefore hypothesized that: (i) the F_1_ hybrids outperformed their parental lines for leaf mineral content; (ii) the inheritance of leaf mineral content in spider plant are controlled by both additive and non-additive gene action; (iii) there are lines and hybrids with good combining ability effects for leaf mineral content; (iv) estimates of combining ability are good predictors for mean hybrids performance and heterosis level for leaf mineral content in *G. gynandra*; (v) the leaf mineral elements content in *G. gynandra* are correlated; and (vi) the correlations between leaf mineral elements content are stable from parents to hybrids.

## Materials and methods

### Plant material

Twenty-six advanced lines, derived from 26 accessions originating from various Asian and African countries (Table 1) and self-pollinated, were used in this study. The lines were separated into two groups; 12 and 14 lines used as females and males, respectively, based on the male/staminate flowers/pollen and female/hermaphrodite flowers production ability (Table 1) as most individual plants in the species were reported to be andromonoecious with differential flowers productivity [25,26]. Males were crossed with females in a North Carolina design II during two summer seasons (season 1, from October 2018 to February 2019 and season 2, from October 2019 to March 2020) in a greenhouse at the Controlled Environment Facility (29°46′ S, 30°58′ E) of the University of KwaZulu-Natal, Pietermaritzburg Campus, South Africa. Across the two crossing seasons, a total of 118 successful unique single crosses with sufficient seeds were generated for evaluation (S1 Table). In addition, each line was self-pollinated during each crossing season. Crosses were performed following the protocol developed by Zohoungbogbo et al. [42].

**Table 1:** List of 26 advanced lines of *G. gynandra* used as parents to generate the 118 hybrids used in this study and their origin.

Lines	Genebank of the original accession	Country of origin	Continent	Parent
P01	WorldVeg	Malaysia	Asia	Female
P02	WorldVeg	Thailand	Asia	Female
P03	WorldVeg	Malaysia	Asia	Female
P04	WorldVeg	Thailand	Asia	Female
P05	WorldVeg	Malaysia	Asia	Female
P06	WorldVeg	Lao People’s Democratic Republic	Asia	Female
P07	WorldVeg	Lao People’s Democratic Republic	Asia	Female
P08	WorldVeg	Malawi	Africa	Female
P09	KENRIK	Kenya	Africa	Female
P10	GBioS	Benin	Africa	Female
P11	GBioS	Benin	Africa	Female
P12	GBioS	Benin	Africa	Female
P13	WorldVeg	Lao People’s Democratic Republic	Asia	Male
P14	WorldVeg	Lao People’s Democratic Republic	Asia	Male
P15	KENRIK	Kenya	Africa	Male
P16	KENRIK	Kenya	Africa	Male
P17	KENRIK	Kenya	Africa	Male
P18	WorldVeg	Kenya	Africa	Male
P19	WorldVeg	Zambia	Africa	Male
P20	WorldVeg	Uganda	Africa	Male
P21	WorldVeg	Uganda	Africa	Male
P22	WorldVeg	Uganda	Africa	Male
P23	WorldVeg	Kenya	Africa	Male
P24	WorldVeg	Zambia	Africa	Male
P25	GBioS	Togo	Africa	Male
P26	GBioS	Togo	Africa	Male

GBioS: Genetics, Biotechnology and Seed Science Unit, University of Abomey-Calavi; WorldVeg: World Vegetable Center; KENRIK: Kenya Resource Center for Indigenous Knowledge.

### Experimental design and growth conditions

The 26 parental lines and 118 F_1_ hybrids were grown in 2019 (March to June) and 2020 (September to December) using an alpha design (10 incomplete blocks with 12 entries per incomplete block for hybrids and seven incomplete blocks with four entries per incomplete block for parents) with two replications in a greenhouse at the Controlled Environment Facility of the University of KwaZulu-Natal, Pietermaritzburg Campus, South Africa. The parents were blocked separately from the hybrids. Seeds of all genotypes were pretreated by heating at 40 °C for three days to improve germination before sowing in cell trays filled with growing media. Cell trays were established in the greenhouse, and germination began three days after planting. Seedlings were grown for four weeks, after which they were transplanted.

In 2019, seedlings were transplanted into a single-row plot, 1 m in length, with a spacing of 20 cm between and within rows on raised beds measuring 1 m in width and 1 m in height. The soil had a clay content of 38.5%, a bulk density of 1 g/cm³, and a pH (KCl) of 5.6. The soil contained 78 mg kg^-1^ of phosphorus, 133 mg kg^-1^ of potassium, 2576 mg kg^-1^ of calcium, 384 mg kg^-1^ of magnesium, 22 mg kg^-1^ of zinc, 11 mg kg^-1^ of manganese, 5 mg kg^-1^ of copper, 1900 mg kg^-1^ of nitrogen, and 2% of organic carbon. In 2020, seedlings were transplanted into 10-liter pots with three plants per pot. Pots were filled with composted pine-bark growing media. The growing media was characterized by 31% of carbon, 11 g kg^-1^ of nitrogen, 13 g kg^-1^ of calcium, 3300 mg kg^-1^ of magnesium, 2500 mg kg^-1^ of potassium, 3400 mg kg^-1^ of phosphorus, 470 mg kg^-1^ of sodium, 181 mg kg^-1^ of zinc, 42 mg kg^-1^ of copper, 1034 mg kg^-1^ of manganese and 7374 mg kg^-1^ of iron on dry matter basis.

In both years, weeds were controlled manually while plants were watered daily using an automated drip irrigation with 1 litre per pot. Before transplanting, basal fertilizer composed of N:P: K (2:3:2) at a dose of 150 kg ha^-1^ was applied, and limestone ammonium nitrate (28% N) was used as topdressing two weeks after transplanting at a dose of 100 kg ha^-1^. The greenhouse structure was made of corrugated polycarbonate material, which reduced the outside solar irradiance by 50% on average. Therefore, the maximum of solar radiation was 356 and 567 W m^-2^ in 2019 and 2020, respectively. In 2019, the day temperature varied between 20 and 35°C and the night temperature ranged from 15 to 25°C (S1 Fig). At the same time, the relative humidity was between 75 and 87% at night and 45 and 70% during day hours. In 2020, while the day temperature ranged from 25 and 38°C and the night temperature ranged between 20 and 25°C. The day relative humidity was between 50 and 80% and between 80 and 90% at night (S1 Fig).

### Mineral analysis

A sample of 20 g of tenders and edible fresh leaves per genotype was randomly collected from all the plants in each replicate in paper bags four weeks after transplanting, meaning eight weeks old plants from sowing date. The leaves were picked from plants between 08:00 and 11:00 am. The samples were immediately transported to the laboratory, washed, and oven-dried at 65°C for 72 h.

In 2019, after cooling, the dried leaves were ground into a powder using a mortar and pestle and then sieved through a 1 mm screen sieve. Two independent replicates of 0.5 g each of sieved powder were weighed in porcelain crucibles using an analytical balance (D&T, ES-E200A, max = 200 g, d = 0.1 mg, China). Samples were then ashed in a muffle furnace at 550°C for 2 hours. The obtained ashes were digested using 10 ml of double acid composed of nitric acid (HNO_3_, 65%, Merck, Germany) and hydrochloric acid (HCl, 32%, Merck, Germany) mixed in a ratio of 1:3 [43]. The resultant mixtures were placed on a hot plate at 250°C for 30 min and later cooled for 1 hour. Digestates were filtered using Whatman paper Grade 1 (Qualitative Filter Paper Standard Grade, circle, 125 mm, Merck, Germany) into a 100 ml volumetric flask and made up to the mark using deionized water. The resultant solutions were analyzed using a fast sequential atomic absorption spectrometer (Varian AA280FS, Varian Inc., Mulgrave, Victoria, Australia) for calcium (Ca), copper (Cu), iron (Fe), zinc (Zn), potassium (K), manganese (Mn), magnesium (Mg) and sodium (Na). Flame atomic absorbance spectroscopy was used for all elements except potassium, for which flame atomic emission spectroscopy was employed. The wavelengths used were 324.8 nm for Cu, 248.3 nm for Fe, 279.5 nm for Mn, 766.5 nm for K, 213.9 nm for Zn, 422.7 nm for Ca, 285.2 nm for Mg, and 589.0 nm for Na. Multielement standard solution IV (23 elements) (1000 mg l^-1^ in HNO_3_ Suprapur® 6.5%) was purchased from Merck, KGaA, Darmstadt, Germany, and used for calibration. The phosphorus was analyzed in the digested solution according to the 4500-P E ascorbic acid method [44] at 670 nm using an Alpha UV–VIS spectrophotometer (Spectronic Unicam, Berlin, Germany) [45].

In 2020, due to the failure of the atomic absorption spectrometer, the dried plant materials were sent to the Plant Laboratory of the KwaZulu-Natal Department of Agriculture and Rural Development at CEDARA Research station for analysis of leaf elemental composition (Ca, Cu, Fe, K, Mn, Mg, Na, P and Zn). The study was performed based on Hunter [46] with slight modifications [47], using an inductively coupled plasma-optical emission spectrometer (ICP–OES) (Agilent 5800 VDV, Agilent Technologies Australia (M) Pty Ltd. Inc., Mulgrave, Australia). Mineral concentrations were reported in mg kg^-1^ on a dry weight basis (mg kg^-1^ DW) for microelements (Cu, Fe, Mn, and Zn) and in g kg^-1^ on a dry weight basis (g kg^-1^ DW) for macroelements (Ca, K, Mg, P, and Na). Data are presented in S2 Table.

### Statistical analysis

The software R version 4.4.0 [48] was used to perform all statistical analyses. Before proceeding to the analyses, the quality of the data was assessed, mainly for outlier detection with the Bonferroni–Holm test based on studentized residuals at the level of significance of 5%, as recommended by Bernal-Vasquez et al. [49]. Data normality was assessed using the Shapiro–Wilk test. Descriptive statistics (minimum, maximum, mean, coefficient of variation, standard error) were used to characterize the parents and hybrids using the function *describe* from the R package *psych* [50]. The significance of differences between the overall means of parents and hybrids was tested using the *t-test* or *the Wilcoxon test* when necessary. For each mineral content, estimates of variance components were computed per year and across years. Per year, each hybrid and parental dataset was analyzed separately for overall genotypic variance components and adjusted means (best linear unbiased predictors - BLUPs) using the following statistical model 1:


$$y_{ik}=\;\mu +\;R_{k}+G_{i}+\varepsilon_{ik}$$ (1)

where $y_{ik}$ is the phenotypic observation of the *i*^*th*^ genotype (hybrid or parental line) in the *k*^*th*^ replicate, μ is the overall mean, $R_{k}$ is the random effect of the *k*^*th*^ replicate, $G_{i}$ is the random effect of the *i*^*th*^ genotype, and $\varepsilon_{ik}$ is the random residual.

Standard broad-sense heritability was calculated according to Hallauer et al. [37] as follows:


$$\begin{array}{c} H^{2}=\sigma_{G}^{2}/\left(\sigma_{G}^{2}+\sigma_{e}^{2}/r\right) \end{array}$$ (2)

where $\sigma_{G}^{2}$ was the total genotypic variance, $\sigma_{e}^{2}$ was the residual variance, and r was the number of replications.

Across years, estimates of variance components for overall genotypic effects were computed using the following statistical model:


$$y_{ijk}=\;\mu +\;Y_{j}+R_{k}\left(Y_{j}\right)+G_{i}+{GY}_{ij}+\varepsilon_{ijk}$$ (3)

in which $y_{ijk}$ was the phenotypic observation of the *i*^*th*^ genotype (hybrid or parental line) in the *k*^*th*^ replicate at the *j*^*th*^ year, μ was the overall mean, $Y_{j}$ was the random effect of the *j*^*th*^ year, $R_{k}\left(Y_{j}\right)$ was the random effect of the *k*^*th*^ replicate within the *j*^*th*^ year, $G_{i}$ was the random effect of the *i*^*th*^ genotype, ${GY}_{ij}$ was the random effect of the interaction between the *i*^*th*^ genotype and the *j*^*th*^ year, and $\varepsilon_{ijk}$ was the random residual. Heterogeneous residual variances were assumed among years. Across years, broad-sense heritability was calculated according to Hallauer et al. [37] using the following formula:


$$\begin{array}{c} H^{\text{2}}=\sigma_{G}^{\text{2}}/\left(\sigma_{G}^{\text{2}}+\sigma_{G\times Y}^{\text{2}}/n+\sigma_{e}^{\text{2}}/nr\right) \end{array}$$ (4)

where $\sigma_{G}^{2}$ was the total genotypic variance, $\sigma_{G\times Y}^{2}$ was the genotype × year interaction variance, $\sigma_{e}^{2}$ was the residual variance, *r* was the number of replications, and *n* was the number of years.

Using the hybrids’ data only, the following statistical model (5) was used to determine the variance components of the specific combining ability (SCA) and general combining ability (GCA) in each year:


$$Y_{fmk}=\mu +\alpha_{f}+\beta_{m}+\gamma_{fm}+r_{k}+\varepsilon_{fmk}$$ (5)

where $y_{fmk}$ was the phenotypic observation of the hybrid between the *f*^*th*^ female and *m*^*th*^ male in the *k*^*th*^ replicate; *µ* was the overall mean; $\alpha_{f}$ was the random GCA effect of the *f*^*th*^ female; $\beta_{m}$ was the random GCA effect of the *m*^*th*^ male; $\gamma_{fm}$ was the random SCA effect of the cross between the *f*^*th*^ female and the *m*^*th*^ male; $r_{k}$ was the random effect of the *k*^*th*^ replicate; and $\varepsilon_{fmk}$ was the random residual.

Across years, the following statistical model (6) was used to determine the variance components of the specific combining ability (SCA) and general combining ability (GCA):


$$Y_{fmjk}=\mu +{ry}_{kj}+y_{j}+\alpha_{f}+\beta_{m}+\gamma_{fm}+{\alpha y}_{fj}+{\beta y}_{mj}+{\gamma y}_{fmj}+\varepsilon_{fmkj}$$ (6)

where $y_{fmjk}$ was the phenotypic observation of the hybrid between the *f*^*th*^ female and *m*^*th*^ male in the *k*^*th*^ replicate at the *j*^*th*^ year; *µ* was the overall mean; $\alpha_{f}$ was the random GCA effect of the *f*^*th*^ female; $\beta_{m}$ was the random GCA effect of the *m*^*th*^ male; $\gamma_{fm}$ was the random SCA effect of the cross between the *f*^*th*^ female and the *m*^*th*^ male; ${\alpha y}_{fj}$ was the random effect of the interaction between the GCA effect of the *f*^*th*^ female and the *j*^*th*^ year; ${\beta y}_{mj}$ was the random effect of the interaction between the GCA effect of the *m*^*th*^ male and the *j*^*th*^ year; ${\gamma y}_{fmj}$ was the random effect of the interaction between the SCA effect of the cross between the *f*^*th*^ female and the *m*^*th*^ male and the *j*^*th*^ year; $\begin{array}{c} {ry}_{kj} \end{array}$ was the random effect of the *k*^*th*^ replicate within the *j*^*th*^ year; and $\varepsilon_{fmkj}$ was the random residual.

All linear mixed-effects models were fitted using the restricted maximum likelihood (REML) implemented in the “ASReml-R” package version 4.1.0.160 [51]. The likelihood ratio test [52] was used to test the significance of the variance components using the function *lrt* implemented in the ASReml-R package. The additive genetic variance ($\sigma_{A}^{2}$), dominance genetic variance ($\sigma_{D}^{2}$), total phenotypic variance ($\sigma_{P}^{2}$), and narrow-sense ($h^{2}$) heritability estimates in each year were determined according to Hallauer et al. [37] and Isik et al. [53] as follows:


$$\sigma_{A}^{2}=2(\sigma_{GCA-M}^{2}+\sigma_{GCA-F}^{2})$$ (7)

$$\sigma_{D}^{2}=4\sigma_{SCA-F\times M}^{2}$$ (8)

$$\begin{array}{c} \sigma_{P}^{2}=\sigma_{G}^{2}+\sigma_{e}^{2}/r \end{array}$$ (9)

$$\begin{array}{c} h^{2}=\sigma_{A}^{2}/\sigma_{P}^{2} \end{array}$$ (10)

where $\sigma_{A}^{2}$ was the additive genetic variance, $\sigma_{GCA-M}^{2}$ was the male GCA variance, $\sigma_{GCA-F}^{2}$ was the female GCA variance, $\sigma_{D}^{2}$ was the dominance genetic variance, $\sigma_{P}^{2}$ was the total phenotypic variance, $\sigma_{SCA-F\times M}^{2}$ was the SCA variance, $\sigma_{e}^{2}$ was the residual variance, and *r* was the number of replications.

The average degree of dominance was computed as D =$\sqrt{\left(\text{2}\sigma_{D}^{2}/\sigma_{A}^{2}\right)}$ [54]. The phenotypic best linear unbiased predictors (BLUPs) associated with the general combining ability effect of each female (GCA_f_) and male (GCA_m_) parent and the specific combining ability effect of each cross (SCA_fm_) were derived from model 5 according to Isik et al. [53]. BLUPs associated with the combining ability effects were used due to the incomplete factorial mating design and interest in the family represented by each parental line. In addition, BLUPs were used because of their good predictive accuracy as they have high correlation with the actual values and have been recommended for phenotypic selection in plant breeding [55–57]. The significance of GCA and SCA effects was evaluated using a two-tailed t-test, at the probability levels of 0.05, 0.01, and 0.001 [58]. The importance of dominance and additive gene effects was assessed through the predictability ratio of Baker [59] using the following formula:


$$\begin{array}{c} Predictability\;ratio=\left(\sigma_{GCA-M}^{2}+\sigma_{GCA-F}^{2}\right)/\left(\sigma_{GCA-M}^{2}+\sigma_{GCA-F}^{2}+\sigma_{SCA-F\times M}^{2}\right) \end{array}$$ (11)

where $\sigma_{GCA-M}^{2}$ was the male GCA variance, $\sigma_{GCA-F}^{2}$ was the female GCA variance, and $\sigma_{SCA-F\times M}^{2}$ was the SCA variance.

For heterosis analysis, the adjusted means of each parent and hybrid generated from model 1 were used to estimate the heterosis level. Mid-parent heterosis (MPH) and best-parent heterosis (BPH) were computed for each hybrid as follows:


$$\begin{array}{c} MPH\;(\%)=[{(F}_{1}-MP)/MP]\times 100 \end{array}$$ (12)

$$\begin{array}{c} BPH\;(\%)=[{(F}_{1}-BP)/BP]\times 100 \end{array}$$ (13)

where $F_{1}$ was the adjusted mean value of the hybrid, MP was the mid-parent adjusted mean value computed as the average adjusted mean values between the two parents of the hybrid, and BP was the adjusted mean value of the best parent.

The genetic advance (GA) for each trait was computed as *GA = i × *H**^*2*^* × σ*_*P*_, where *σ*_*P*_ was the phenotypic standard deviation, *H*^*2*^ was the broad-sense heritability, and *i* was the standardized selection differential at the selection intensity of 5% (i = 2.06) [60]. Genetic advance over mean (GAM) was further computed as *GAM = (GA/µ*) x 100**, where µ is the overall mean of the trait. Spearman correlation coefficients between combining ability, heterosis and mean performance of the parents and hybrids for all characteristics and their level of significance were performed using the function *corr* from the R package *Hmisc* [61].

## Results

### Performance of parents and hybrids

Variations in the leaf contents of iron, zinc, copper, manganese, calcium, potassium, magnesium, phosphorus, and sodium for both parents and hybrids are summarized in Table 2. Trait variability ranged from low to high and depended on the trait (Table 2). For both parents and hybrids, the highest variability was observed for manganese, with a coefficient of variation (CV) of 83% and 94% for parents and hybrids, respectively. In contrast, the lowest coefficient of variation (CV) was observed for potassium (CV = 17%) in parents and for phosphorus in hybrids (21%). For both parents and hybrids, the order of mineral element content in the leaves was potassium > calcium > phosphorus > magnesium > sodium > iron or manganese > zinc > copper.

**Table 2:** Descriptive statistics of nine minerals content investigated in 118 hybrids and their 26 parents of *Gynandropsis gynandra* evaluated over two years (2019 and 2020) in a greenhouse.

Minerals	Mean		Minimum		Maximum		Standard error		Coefficient of variation (%)	
	Hybrids	Parents	Hybrids	Parents	Hybrids	Parents	Hybrids	Parents	Hybrids	Parents
**Macroelement*s***										
**Ca (g kg**^**-1**^)	23.45	24.49	10.92	11.42	49.82	53.22	0.38	0.92	35.33	37.61
**Mg (g kg**^**-1**^)	4.93	4.99	1.37	1.53	9.63	9.38	0.08	0.18	34.62	36.74
**K (g kg**^**-1**^)	34.25	25.47	16.13	12.26	63.73	34.96	0.51	0.45	32.41	17.7
**Na (g kg**^**-1**^)	0.9	1.05	0.34	0.56	3.47	1.73	0.02	0.02	38.81	23.66
**P (g kg**^**-1**^)	9.55	7.99	3.93	4.84	15.55	13.39	0.09	0.2	20.99	25.12
**Microelements**										
**Fe (mg kg**^**-1**^)	151.52	158.25	41.7	67.76	527.96	430.72	2.73	6.64	39.01	41.93
**Zn (mg kg**^**-1**^)	73.19	56.07	27.88	28.47	224.95	104.31	1.28	1.43	37.95	25.56
**Cu (mg kg**^**-1**^)	9.66	10.63	1.73	3.03	27.04	25.1	0.15	0.35	34.61	33.16
**Mn (mg kg**^**-1**^)	192.03	109.89	14.91	18.2	558.04	433.08	7.35	10.37	83	94.4

**Fig 1:**
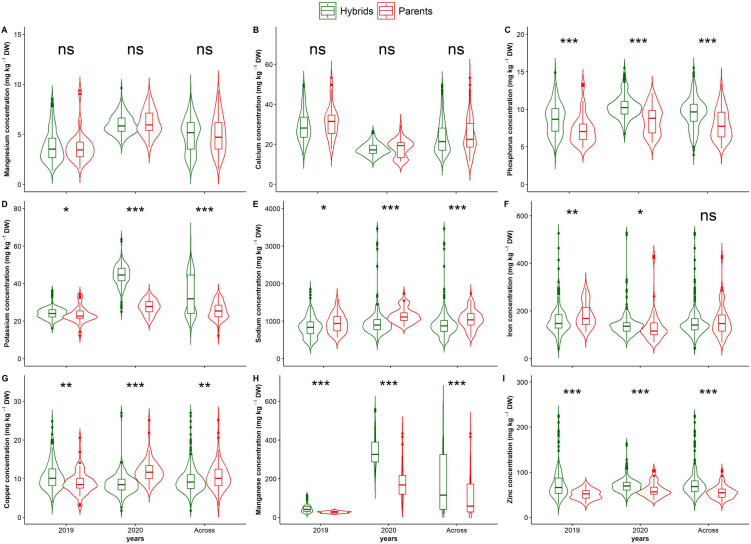
Comparison of hybrids and parents’ performance for leaf mineral content in 118 hybrids and their 26 parents of *G. gynandra* evaluated in 2019 and 2020. ***, **, * indicate significance at the 0.001, 0.01, and 0.05 probability level, respectively.

Overall, significant differences (p < 0.05) were observed between the mean of parents and hybrids for all microelements (Fe, Zn, Cu, Mn) and phosphorus, potassium, and sodium (Fig 1) in each year and across years, except for iron. Hybrids performed better than their parents for Zn, Mn, P, and K, with average increases of 30.5%, 74.7%, 34.4%, and 19.5%, respectively. In contrast, the mean Na content of the parents was higher (14.3%) than that of the hybrids. While the mean of hybrids was lower than that of parents for Fe in 2019, a higher mean hybrid value was observed in 2020. The inverse was noticed for Cu.

### Variance components and heritability estimates

Combined analysis across years showed that year and genotype by year interaction variances were highly significant for mineral contents in both parents and hybrids (Table 3). Genotypic variances were significant for K and P in parents and for Ca and Mg in hybrids only. Irrespective of the type of population (hybrids or parents), genotype × year interaction variances were higher than the genotypic variance (Table 3). The partitioning of genotypic variance in hybrids showed that general combining ability (GCA) variance for females ($\sigma_{\text{GCA}-\text{F}}^{\text{2}}$) and males ($\sigma_{\text{GCA}-\text{M}}^{\text{2}}$) were significantly different from zero for Ca only. Variances due to female general combining ability × year interaction ($\sigma_{\text{GCA}-\text{F}\times \text{Y}}^{\text{2}}$) were significant for all mineral contents except Mn and Mg. In contrast, variances due to male general combining ability × year interaction ($\sigma_{\text{GCA}-\text{M}\times \text{Y}}^{\text{2}}$) were significant for Ca and P. Specific combining ability × year interaction variances ($\sigma_{\text{SCA}-\text{F}\times \text{M}\times \text{Y}}^{\text{2}}$) were substantial for all minerals content and greater than any other combining ability variances alone or with year interaction (Table 3).

**Table 3:** Estimates of variance components for nine mineral elements in 118 hybrids and their 26 parents of *Gynandropsis gynandra* evaluated over two years in a greenhouse.

Source variation	Ca	K	Mg	Na	P	Cu	Fe	Mn	Zn
**Parents**									
$\sigma_{Y}^{2}$	85.01 ± 122.34***	7.91 ± 11.87*	2.83 ± 4.11***	0.02 ± 0.03 ± ns	0.69 ± 1.12*	4.00 ± 6.18*	1030.48 ± 1649.51*	12704.95 ± 18081.32***	40.60 ± 64.96*
$\sigma_{G}^{2}$	2.65 ± 8.22 ns	5.97 ± 3.49*	4.27 10^−07^ ± 0 ns	3.08 10^−04^ ± 9.49 10^−04^ ns	1.3 ± 0.81*	2.07 ± 1.88 ns	7.56 10^−04^ ± 0 ns	3.13 ± 50.81 ns	54.63 ± 39.41 ns
$\sigma_{G\times Y}^{2}$	35.84 ± 10.86***	7.95 ± 2.85***	1.87 ± 0.39***	0.04 ± 0.01***	2.34 ± 0.7***	5.07 ± 1.98***	2930.04 ± 703.69***	13.13 ± 51.15 ns	122.46 ± 37.51***
$\sigma_{e}^{2}$	3.09 ± 0.72	3.46 ± 0.81	0.07 ± 0.01	6.54 10^−03^ ± 1.56 10^−3^	0.1 ± 0.02	3.55 ± 0.82	924.12 ± 190.03	4156.08 ± 824.46	10.71 ± 2.19
$H^{2}$	0.12 ± 0.36	0.55 ± 0.19	4.45 10^−07^ ± 9.10 10^−08^	0.01 ± 0.40	0.52 ± 0.2	0.38 ± 0.26	4.46 10^−07^ ± 9.14 10^−08^	0.00 ± 0.05	0.46 ± 0.22
**Hybrids**									
$\sigma_{Y}^{2}$	68.70 ± 97.56**	205.55 ± 291.01***	2.30 ± 3.30**	0.004 ± 001 ns	1.25 ± 1.89*	1.91 ± 2.92*	171.38 ± 278.12*	43916.29 ± 62145.09 **	1.95 ± 11.14 ns
$\sigma_{G}^{2}$	7.99 ± 3.23**	0.72 ± 1.79 ns	0.4 ± 0.16**	0.00 ± 0.01 ns	0.23 ± 0.3 ns	0.00 ± 0.00 ns	271.11 ± 303.23 ns	127.57 ± 303.17 ns	82.04 ± 70.95 ns
$\sigma_{GCA-F}^{2}$	5.19 ± 5.16 ns	1.29 ± 1.61 ns	0.15 ± 0.09*	0.01 ± 0.01 ns	0.40 ± 0.36 ns	0.00 ± 0.00 ns	91.94 ± 221.98 ns	37.53 ± 89.05 ns	56.66 ± 99.03 ns
$\sigma_{GCA-M}^{2}$	2.38 ± 1.84 ns	1.41 ± 1.2 ns	0.33 ± 0.18**	0.00 ± 0.00 ns	0.00 ± 0.00 ns	0.10 ± 0.2 ns	0.00 ± 0.00 ns	39.56 ± 210.96 ns	7.82 ± 15.71 ns
$\sigma_{SCA-F\times M}^{2}$	0.36 ± 1.66 ns	0.00 ± 0.00 ns	0.00 ± 0.00 ns	0.00 ± 0.00 ns	0.00 ± 0.00 ns	0.00 ± 0.00 ns	176.06 ± 280.54 ns	66.75 ± 301.52 ns	12.61 ± 49.63 ns
$\sigma_{G\times Y}^{2}$	25.67 ± 3.4***	18.32 ± 2.45***	1.28 ± 0.17***	0.11 ± 0.01***	2.74 ± 0.39***	8.34 ± 0.85***	2793.87 ± 390.55***	3113.3 ± 414.17***	675.54 ± 89.13***
$\sigma_{GCA-F\times Y}^{2}$	9.33 ± 4.69***	2.37 ± 1.66**	0.00 ± 0.00 ns	0.01 ± 0.01*	0.51 ± 0.3***	2.52 ± 0.99***	338.23 ± 262.33*	0.00 ± 0.00 ns	216.40 ± 113.59***
$\sigma_{GCA-M\times Y}^{2}$	1.79 ± 1.46*	0.87 ± 1.05 ns	0.09 ± 0.09 ns	0.00 ± 0.01 ns	0.43 ± 0.19***	0.00 ± 0.00 ns	66.14 ± 110.98 ns	300.22 ± 275.53 ns	0.00 ± 0.00 ns
$\sigma_{SCA-F\times M\times Y}^{2}$	15.36 ± 2.29***	13.77 ± 1.48***	1.10 ± 0.11***	0.09 ± 0.01***	1.70 ± 0.2***	6.13 ± 0.69***	2438.36 ± 376.08***	2854.64 ± 406.01***	475.39 ± 66.14***
$\sigma_{e}^{2}$	0.66 ± 0.06	0.82 ± 0.09	0.05 ± 0.00	0.00 ± 0.00	0.42 ± 0.04	1.62 ± 0.16	339.81 ± 32.14	92.86 ± 11.47	12.30 ± 1.20
$H^{2}$	0.55 ± 0.21	0.38 ± 0.19	0.61 ± 0.11	0.32 ± 0.20	0.36 ± 0.22	0.04 ± 0.08	0.37 ± 0.32	0.21 ± 0.47	0.34 ± 0.36

$\sigma_{\text{G}}^{\text{2}}$: genotypic variance; $\begin{array}{c} \sigma_{\text{Y}}^{\text{2}} \end{array}$: year variance; $\begin{array}{c} \sigma_{\text{G}\times \text{Y}}^{\text{2}} \end{array}$: genotype × year interaction variance; $\sigma_{e}^{\text{2}}$, residual variance; $\sigma_{\text{GCA}-\text{F}}^{\text{2}}$: female general combining ability variance; $\sigma_{\text{GCA}-\text{M}}^{\text{2}}$: male general combining ability variance; $\sigma_{\text{SCA}-\text{F}\times \text{M}}^{\text{2}}$: specific combining ability variance; $\sigma_{\text{GCA}-\text{F}\times \text{Y}}^{\text{2}}$: female general combining ability × year interaction variance; $\sigma_{\text{GCA}-\text{M}\times \text{Y}}^{\text{2}}$: male general combining ability × year interaction variance; $\sigma_{\text{SCA}-\text{F}\times \text{M}\times \text{Y}}^{\text{2}}$: specific combining ability × year interaction variance; H^2^: broad-sense heritability; ***, **, *: significantly different from zero at the 0.001, 0.01, and 0.05 probability levels, respectively. ns: not significantly different from zero at the 0.05 level of probability.

Given the significant effect of year and its interaction with genotype and combining abilities, estimates of genetic variance components, heritability estimates, degree of dominance, Baker’s [59] predictability ratio, and genetic advance were estimated in each year and summarized for macroelements (Ca, Mg, Na, K, P) in Table 4 and microelements (Fe, Mn, Cu, Zn) in Table 5. For all minerals, the genotypic variance was higher than the residual variance in both parents and hybrids each year (Tables 4 and 5). The partitioning of genotypic variance in hybrids showed that general combining ability (GCA) variance for females ($\sigma_{\text{GCA}-\text{F}}^{\text{2}}$) was significantly different from zero for Ca, P and Zn each year, for Mg, Na, Fe, Cu, and Mn in 2019, and K in 2020 (Tables 4 and 5). The GCA variance for males ($\sigma_{\text{GCA}-\text{M}}^{\text{2}}$) was significantly different from zero for all macro elements, such as Ca, K, Mg, and P, in both years and for Na in 2019. In contrast, the GCA variance for males ($\sigma_{\text{GCA}-\text{M}}^{\text{2}}$) was not significant for all microelements (Fe, Cu, Mn, Zn) in 2019 and 2020. While estimates of female GCA variance for Zn were larger than the male ones, male GCA variances were greater than female GCA variances for Mg in both years. For all mineral contents, the specific combining ability (SCA) variance ($\sigma_{\text{SCA}-\text{F }\times \text{ M}}^{\text{2}}$) was significantly different from zero (p < 0.05) with an estimate greater than the average and sum of females and males GCA variances in both years. Similarly, the additive variance ($\sigma_{\text{A}}^{\text{2}}$) was lower than the dominance variance ($\sigma_{\text{D}}^{\text{2}}$) for all mineral elements. The degree of dominance was greater than unity for all mineral contents, showing their dominant nature. Overall, the predictability ratio was lower than 0.5 for all mineral contents in both years (Tables 4 and 5).

**Table 4:** Estimates of genetic variance components, heritability, degree of dominance, predictability ratio, and genetic advance for the macroelements in 118 hybrids and their 26 parents of *Gynandropsis gynandra* evaluated in 2019 and 2020.

Source	Ca		K		Mg		P		Na	
Year	2019	2020	2019	2020	2019	2020	2019	2020	2019	2020
**Parents**										
$\sigma_{G}^{2}$	62.33 ± 19***	18.48 ± 5.44***	17.9 ± 5.42***	8.46 ± 3.38***	2.24 ± 0.67***	1.55 ± 0.45***	3.87 ± 1.16***	3.42 ± 0.98***	0.05 ± 0.02***	0.04 ± 0.01***
$\sigma_{e}^{2}$	4.11 ± 1.21	1.5 ± 0.42	0.97 ± 0.28	6.21 ± 1.72	0.08 ± 0.02	0.08 ± 0.02	0.1 ± 0.03	0.09 ± 0.03	0.01 ± 0	0 ± 0
$H^{2}$	0.97 ± 0.01	0.96 ± 0.02	0.97 ± 0.01	0.73 ± 0.11	0.98 ± 0.01	0.98 ± 0.01	0.99 ± 0.01	0.99 ± 0.01	0.91 ± 0.04	0.96 ± 0.01
**Mean**	31.34 ± 1.49	18.17 ± 0.85	23.4 ± 0.78	27.39 ± 1.52	3.74 ± 0.2	6.15 ± 0.19	7.33 ± 0.25	8.61 ± 0.21	0.94 ± 0.09	1.15 ± 0.04
**GAM**	51.06	47.77	36.76	18.71	81.75	41.16	54.91	43.96	46.26	34.58
**Hybrids**										
$\sigma_{G}^{2}$	58.38 ± 7.66***	8.66 ± 1.19***	9.23 ± 1.23***	29.41 ± 3.93***	2.53 ± 0.33***	0.83 ± 0.11***	4.21 ± 0.56***	1.51 ± 0.25***	0.08 ± 0.01***	0.15 ± 0.02***
$\sigma_{GCA-F}^{2}$	27.75 ± 13.04***	1.4 ± 0.88**	0.73 ± 0.66 ns	6.6 ± 3.82***	0.26 ± 0.18**	0.04 ± 0.05 ns	1.5 ± 0.74***	0.28 ± 0.19**	0.03 ± 0.01***	0.01 ± 0.01 ns
$\sigma_{GCA-M}^{2}$	6.69 ± 3.87***	1.78 ± 0.99***	1.09 ± 0.79*	3.41 ± 2.48*	0.66 ± 0.34***	0.21 ± 0.11***	0.67 ± 0.38***	0.19 ± 0.15*	0.01 ± 0.01**	0.00 ± 0.01 ns
$\sigma_{SCA-F\times M}^{2}$	25.68 ± 3.79***	5.53 ± 0.87***	7.52 ± 1.12***	20.55 ± 3.12***	1.62 ± 0.24***	0.58 ± 0.09***	2.16 ± 0.33***	1.09 ± 0.22***	0.04 ± 0.01***	0.14 ± 0.02***
$\sigma_{e}^{2}$	0.48 ± 0.06	0.84 ± 0.11	0.29 ± 0.04	1.31 ± 0.17	0.04 ± 0.01	0.06 ± 0.01	0.16 ± 0.02	0.71 ± 0.09	0.00 ± 0.00	0.01 ± 0.00
$\sigma_{A}^{2}$	68.87 ± 27.11	6.36 ± 2.61	3.64 ± 2.09	20.03 ± 9.12	1.83 ± 0.76	0.49 ± 0.24	4.34 ± 1.66	0.93 ± 0.48	0.08 ± 0.03	0.03 ± 0.03
$\sigma_{D}^{2}$	102.7 ± 15.14	22.14 ± 3.48	30.06 ± 4.48	82.2 ± 12.48	6.48 ± 0.96	2.32 ± 0.36	8.64 ± 1.31	4.34 ± 0.87	0.16 ± 0.02	0.56 ± 0.08
$h^{2}$	0.4 ± 0.1	0.22 ± 0.08	0.11 ± 0.06	0.19 ± 0.08	0.22 ± 0.08	0.17 ± 0.07	0.33 ± 0.09	0.17 ± 0.08	0.32 ± 0.1	0.05 ± 0.05
$H^{2}$	0.99 ± 0.01	0.95 ± 0.01	0.98 ± 0.00	0.98 ± 0.00	0.99 ± 0.00	0.97 ± 0.01	0.98 ± 0.00	0.81 ± 0.04	0.99 ± 0.00	0.98 ± 0.00
$h_{F}^{2}$	0.65 ± 0.22	0.19 ± 0.12	0.09 ± 0.08	0.26 ± 0.14	0.12 ± 0.09	0.06 ± 0.06	0.46 ± 0.18	0.20 ± 0.13	0.5 ± 0.19	0.09 ± 0.08
$h_{M}^{2}$	0.16 ± 0.09	0.25 ± 0.13	0.13 ± 0.09	0.13 ± 0.09	0.32 ± 0.14	0.29 ± 0.14	0.20 ± 0.11	0.13 ± 0.10	0.14 ± 0.09	0.01 ± 0.05
**Degree of dominance**	1.73 ± 0.37	2.64 ± 0.61	4.07 ± 1.27	2.86 ± 0.72	2.66 ± 0.61	3.08 ± 0.81	1.99 ± 0.42	3.05 ± 0.9	2.07 ± 0.46	6.18 ± 3.23
**Predictability ratio**	0.57 ± 0.1	0.36 ± 0.11	0.19 ± 0.10	0.33 ± 0.11	0.36 ± 0.11	0.3 ± 0.11	0.5 ± 0.11	0.3 ± 0.12	0.48 ± 0.11	0.09 ± 0.09
**Mean**	29.32 ± 0.49	17.57 ± 0.74	24.1 ± 0.38	44.39 ± 0.89	3.85 ± 0.15	6.01 ± 0.27	8.73 ± 0.28	10.36 ± 0.65	0.84 ± 0.03	0.97 ± 0.09
**GAM**	53.57	33.69	25.76	24.89	84.80	30.62	47.99	21.93	66.89	82.60

$\sigma_{\text{A}}^{\text{2}}$: additive genetic variance; $\sigma_{\text{D}}^{\text{2}}$: dominance genetic variance; $\sigma_{\text{GCA}-\text{F}}^{\text{2}}$: female general combining ability variance; $\sigma_{\text{GCA}-\text{M}}^{\text{2}}$: male general combining ability variance; $\sigma_{\text{SCA}-\text{F}\times \text{M}}^{\text{2}}$: specific combining ability variance; $\sigma_{e}^{\text{2}}$: residual variance; $\sigma_{\text{G}}^{\text{2}}$: genotypic variance; H^2^: broad-sense heritability; h^2^: narrow-sense heritability; $h_{F}^{2}$: narrow-sense heritability for females; $h_{F}^{2}$: narrow-sense heritability for males; GAM: genetic advance over mean. ***, **, *: significantly different from zero at the 0.001, 0.01, and 0.05 probability levels, respectively. ns: not significantly different from zero at the 0.05 level of probability.

**Table 5:** Estimates of genetic variance components, heritability, degree of dominance, predictability ratio, and genetic advance for the microelements in 118 hybrids and their 26 parents of *Gynandropsis gynandra* evaluated in 2019 and 2020.

Source	Fe		Cu		Mn		Zn	
Year	2019	2020	2019	2020	2019	2020	2019	2020
**Parents**								
$\sigma_{G}^{2}$	1988.93 ± 702.42***	3925.55 ± 1273.13***	8.9 ± 2.79***	4.2 ± 2.26*	16.13 ± 6.39***	8467.17 ± 2401.96***	111.33 ± 34.31***	238.41 ± 69.04***
$\sigma_{e}^{2}$	731.98 ± 211.3	1087.85 ± 301.72	1.12 ± 0.32	6.26 ± 1.77	9.95 ± 2.87	50.04 ± 13.88	9.87 ± 2.85	11.27 ± 3.19
$H^{2}$	0.84 ± 0.06	0.88 ± 0.05	0.94 ± 0.02	0.57 ± 0.17	0.76 ± 0.1	1 ± 0	0.96 ± 0.02	0.98 ± 0.01
**Mean**	183.37 ± 17.65	135.07 ± 21.92	9.1 ± 0.73	12.04 ± 1.5	26.74 ± 1.96	186.64 ± 4.99	51.16 ± 2.18	60.61 ± 2.42
**GAM**	46.04	89.55	65.52	26.55	27.06	101.41	41.58	51.87
**Hybrids**								
$\sigma_{G}^{2}$	3584.63 ± 498.46***	2574.02 ± 354.39***	10.72 ± 1.56***	6.33 ± 0.89***	345.86 ± 46.08***	6308.96 ± 839.47***	1232.78 ± 162.24***	287.46 ± 38.13***
$\sigma_{GCA-F}^{2}$	909.69 ± 524.4***	11.78 ± 120.12 ns	5.16 ± 2.53***	0.01 ± 0.32 ns	46.63 ± 34.55*	3.16 ± 275.78 ns	502.26 ± 246.67***	44.01 ± 29.3**
$\sigma_{GCA-M}^{2}$	15.77 ± 149.21 ns	97.22 ± 159.77 ns	0.16 ± 0.39 ns	0.05 ± 0.37 ns	0.00 ± 0.00 ns	690.07 ± 594.96 ns	0.00 ± 0.00 ns	19.7 ± 18.81 ns
$\sigma_{SCA-F\times M}^{2}$	2757.39 ± 433.37***	2470.52 ± 378.24***	5.98 ± 1.04***	6.27 ± 0.99***	305.27 ± 42.92***	5726.51 ± 859.67***	746.7 ± 103.64***	228.43 ± 33.98***
$\sigma_{e}^{2}$	412.01 ± 53.87	266.54 ± 34.7	2.29 ± 0.3	0.96 ± 0.13	13.02 ± 1.7	167.4 ± 21.98	16.23 ± 2.12	8.28 ± 1.08
$\sigma_{A}^{2}$	1850.91 ± 1088.4	218.01 ± 412.88	10.65 ± 5.12	0.11 ± 1	93.27 ± 69.09	1386.47 ± 1325.99	1004.52 ± 493.34	127.42 ± 70.55
$\sigma_{D}^{2}$	11029.58 ± 1733.49	9882.07 ± 1512.96	23.91 ± 4.18	25.09 ± 3.97	1221.06 ± 171.67	22906.04 ± 3438.7	2986.8 ± 414.57	913.71 ± 135.9
$h^{2}$	0.14 ± 0.08	0.02 ± 0.04	0.3 ± 0.11	0 ± 0.04	0.07 ± 0.05	0.06 ± 0.05	0.25 ± 0.1	0.12 ± 0.06
$H^{2}$	0.95 ± 0.01	0.95 ± 0.01	0.90 ± 0.02	0.93 ± 0.01	0.98 ± 0.00	0.99 ± 0.00	0.99 ± 0.00	0.99 ± 0.00
$h_{F}^{2}$	0.28 ± 0.15	0 ± 0.05	0.58 ± 0.21	0 ± 0.05	0.14 ± 0.1	0 ± 0.05	0.5 ± 0.19	0.17 ± 0.11
$h_{M}^{2}$	0 ± 0.05	0.04 ± 0.06	0.02 ± 0.04	0.01 ± 0.06	0.00 ± 0.00	0.11 ± 0.1	0.00 ± 0.00	0.08 ± 0.07
**Degree of dominance**	3.45 ± 1.09	9.52 ± 9.31	2.12 ± 0.56	20.98 ± 92.83	5.12 ± 1.98	5.75 ± 2.91	2.44 ± 0.63	3.79 ± 1.14
**Predictability ratio**	0.25 ± 0.12	0.04 ± 0.08	0.47 ± 0.13	0.01 ± 0.08	0.13 ± 0.09	0.11 ± 0.1	0.4 ± 0.12	0.22 ± 0.1
**Mean**	161.44 ± 14.35	141.69 ± 11.26	10.68 ± 1.02	8.65 ± 0.76	44.43 ± 2.53	340.89 ± 9.72	75.17 ± 2.86	71.21 ± 2.06
**GAM**	74.29	71.92	60.03	57.75	85.43	47.68	95.90	48.70

$\sigma_{\text{A}}^{\text{2}}$: additive genetic variance; $\sigma_{\text{D}}^{\text{2}}$: dominance genetic variance; $\sigma_{\text{GCA}-\text{F}}^{\text{2}}$: female general combining ability variance; $\sigma_{\text{GCA}-\text{M}}^{\text{2}}$: male general combining ability variance; $\sigma_{\text{SCA}-\text{F}\times \text{M}}^{\text{2}}$: specific combining ability variance; $\sigma_{e}^{\text{2}}$: residual variance; $\sigma_{\text{G}}^{\text{2}}$: genotypic variance; H^2^: broad-sense heritability; h^2^: narrow-sense heritability; $h_{F}^{2}$: narrow-sense heritability for females; $h_{F}^{2}$: narrow-sense heritability for males; GAM: genetic advance over mean. ***, **, *: significantly different from zero at the 0.001, 0.01, and 0.05 probability levels, respectively. ns: not significantly different from zero at the 0.05 level of probability.

Broad-sense heritability (H^2^) estimates were moderate to high for all minerals in both hybrids and parents per year, ranging between 0.57 and 0.99 for parents and between 0.81 and 0.99 for hybrids (Tables 4 and 5). In contrast, narrow-sense heritability (h^2^) estimates were low to moderate. Low h^2^ values (≤ 0.30) were observed for all microelements each year. Similarly, low h^2^ values were obtained for macro elements in 2019 and 2020, except Na (0.32), P (0.33), and Ca (0.40) in 2019, for which moderate h^2^ values were observed. Furthermore, differential h^2^ values were observed between males and females and varied from year to year (Tables 4 and 5). Genetic gains (> 20% of the current mean of the hybrid population) at 5% selection intensity were significant for all minerals (Tables 4 and 5). The highest genetic gain estimates were 95.90% in 2019 and 82.60% in 2020 for Zn and Na, respectively.

### General combining ability effects of the parents

**Fig 2:**
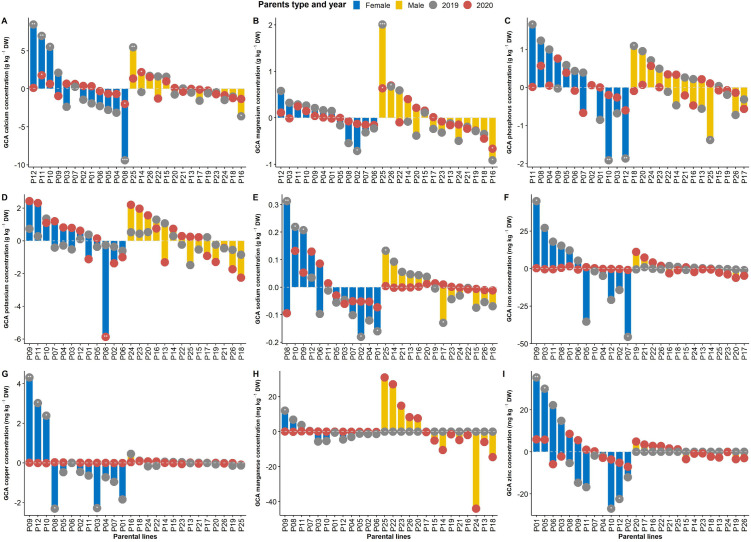
Estimates of general combining ability effects of male and female lines for mineral elements involved in 118 experimental hybrids of *Gynandropsis gynandra* evaluated in 2019 and 2020. ***, **, * refer to estimate of general combining ability effect significantly different from zero at p < 0.001, 0.01, and 0.05, respectively.

Estimates of the general combining ability (GCA) effects of male and female parents are presented in Fig 2. Female parents had significant GCA effects for all minerals, while no significant GCA effects were observed for male parents for Zn, Fe, Mn, and Cu in both years. Some female and male parents displayed multiple significant and positive GCA effects. Good general female combiners included parent P12 for Ca, Mg and Cu; parent P09 for K, Na, Fe, Cu and Mn; P11 for Ca, Mg, K and Fe; parent P10 for Ca, K, Mg, Na and Cu; parent P05 for Zn and P. Parent P01 was good combiner for Zn. Males with multiple positive GCA effects comprised parent P25 for Ca, Mg, Na, and Mn; parents P26 and P15 for Ca and Mg, and P24 for P and K.

### Specific combining ability effects of hybrids

A wide range of specific combining ability (SCA) effects from negative to positive was observed for all mineral content (S2–S10 Fig). The hybrids P10 × P13, P10 × P14, P07 × P20, P02 × P15 and P05 × P25 exhibited highly significant and positive SCA effects for Ca (S2 Fig). Similarly, significant and positive SCA effects for Mg in both years were observed for crosses P12 × P26, P10 × P13, P04 × P17, P02 × P15, and P10 × P14 (S3 Fig). In contrast, negative desirable SCA effects were observed in crosses P10 × P17, P05 × P13, P03 × P23, P08 × P18 and P08 × P15 for Ca, and P08 × P18, P02 × P13, P10 × P17, P05 × P13, and P08 × P19 for Mg. As phosphorus concerned, crosses with positive and significant SCA effects were P05 × P15, P02 × P24, P03 × P24, P09 × P19, P08 × P22, and P08 × P15 and hybrids with negative and significant SCA effects included P09 × P16, P07 × P17, P07 × P21 and P07 × P26 (S4 Fig). While the hybrids P10 × P18, P11 × P16, P12 × P25, P10 × P17, P09 × P24 had significant and positive SCA effects for potassium content, P08 × P16, P01 × P25, P02 × P18, P05 × P13, P08 × P19 had a negative and significant SCA effects (S5 Fig). The crosses such as P10 × P16, P09 × P19, P03 × P25, P12 × P25, P11 × P20 and P02 × P20 displayed highly significant and positive SCA effects for sodium. In contrast, progenies of P05 × P24, P01 × P26, P04 × P15, P08 × P18, P06 × P18 showed negative and significant SCA effects (S6 Fig). For Fe, the highest and most significant SCA effects were observed in hybrid P01 × P21, followed by P09 × P19, P05 × P24, P08 × P22 and P10 × P25. Negative and significant SCA effects were displayed by hybrids P06 × P21, P09 × P24, P07 × P17 and P10 × P20 for Fe content (S7 Fig). Hybrids P02 × P16, P09 × P19, P12 × P16, P08 × P16, P02 × P14 and P12 × P13 were the best for SCA effects, while P11 × P25, P04 × P25, P12 × P26, P09 × P15 had negative and significant SCA effects for copper content (S8 Fig). For Mn content, while crosses with significant and positive SCA effects included P09 × P23, P12 × P13, P09 × P21, P07 × P25, P11 × P22 and P11 × P19, the ones with negative SCA effects included P09 × P15, P06 × P13, P06 × P26, P04 × P20, P04 × P24 and P02 × P13 (S9 Fig). The highest and significantly positive SCA effect for Zn was observed in P01 × P21, followed by P06 × P16, P09 × P18, P11 × P16, and P02 × P16, whereas crosses such as P01 × P16, followed by P09 × P15, P02 × P25, P11 × P16, and P05 × P19 had negative desirable SCA effects (S10 Fig).

### Heterosis

**Fig 3:**
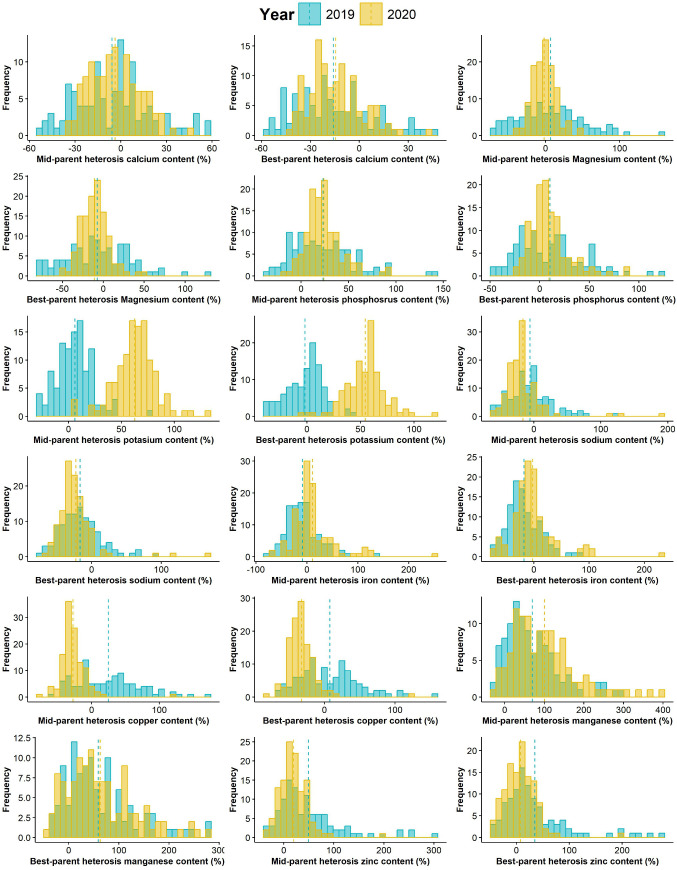
Distribution of mid- and best-parent heterosis for nine mineral elements in 118 experimental hybrids of *Gynandropsis gynandra* evaluated in 2019 and 2020. A dotted line indicates the mean of each year.

The distributions of mid- and best-parent heterosis (MPH and BPH, respectively) for mineral elements are shown in Fig 3. A similar distribution pattern was observed for most traits each year. The heterosis for mineral content ranged between −80.4% and 389.5%, respectively, when mid- and best-parent heterosis were pooled. The species displayed both negative and positive heterosis. For Ca, the hybrids with positive BPH and MPH were P07 × P20, P09 × P20, P09 × P21, and P10 × P14 across years (S11 and S12 Figs). While the hybrids P04 × P17, P07 × P20, P04 × P18, P12 × P25, P10 × P13 and P10 × P14 exhibited positive MBP and BPH for magnesium (S13 and S14 Figs), the crosses P04 × P25, P11 × P25, P11 × P13, P05 × P14, P02 × P14, P08 × P15 had positive mid- and best parents heterosis for phosphorus across years (S15 and S16 Figs). For potassium, P01 × P13, P02 × P14, P10 × P16, P08 × P15 and P10 × P20 were the best (S17 and S18 Figs) and P09 × P19, P10 × P16 and P12 × P25 were identified for sodium (S19 and S20 Figs). The top five crosses with positive mid- and best parents heterosis in both years for iron included P01 × P21, P06 × P16, P09 × P19, P10 × P25 and P11 × P15 (S21 and S22 Figs). Regarding copper, crosses P04 × P15 and P08 × P17 were the best (S23 and S24 Figs). The cross combinations P02 × P15, P04 × P15, P07 × P15, P07 × P20, P09 × P21, and P09 × P23 were the best with positive MPH and BPH for manganese (S25 and S26 Figs). For zinc, the hybrids P01 × P21, P01 × P23, P05 × P14, P06 × P17, P01 × P17, P04 × P18, P05 × P25 displayed positive MPH and BPH (> 30%) across years (S27 and S28 Figs).

### Correlation between combining ability, heterosis and mean performance of the parents and hybrids

The associations between combining ability, heterosis, and mean genotypic values of the parents and hybrids are summarized in Table 6. The per se performance of the parents exhibited a significantly positive correlation with their GCA effects for calcium, potassium, magnesium, and zinc in 2020 only. The F_1_ per se performance was significantly and positively correlated with SCA and the sum of GCA effects (sGCA) of the hybrids’ parents for all minerals (Table 6). The r (SCA, F_1_) was higher than the r (F_1_, sGCA) for all traits. The correlation coefficients between SCA and F_1_ were strong (r ≥ 0.70). In addition, heterosis (both MPH and BPH) showed a highly significant and positive association with SCA and F_1_ hybrid performance for all minerals, ranging from moderate to strong and trait specific. The correlation between the sum of GCA effects of the parents of hybrids and heterosis was variable and depended on the trait and year. In 2019, the sum of GCA effects of hybrid parents had a moderate and positive correlation with MPH and BPH for all minerals. In contrast, no weak correlations were observed between the sum of the GCA hybrid parents and the heterosis in 2020 (Table 6).

**Table 6:** Spearman correlation coefficients between general combining ability effects and parent *per se* performance r(*per se*, GCA), among mid- (MPH) and best- (BPH) parent heterosis, hybrid performance (F_1_) and specific combining ability (SCA) and sum of general combining abilities of hybrids’ parents (sGCA) for nine leaf mineral content in *G. gynandra.*

Traits	Year	r(*per se*, GCA)	r(F_1_, SCA)	r(F_1_, MPH)	r(F_1_, BPH)	r(SCA, MPH)	r(SCA, BPH)	r(F_1_, sGCA)	r(sGCA, MPH)	r(sGCA, BPH)
**Ca**	2019	0.21ns	0.70***	0.75***	0.71***	0.54***	0.49***	0.72***	0.52***	0.50***
	2020	0.68***	0.83***	0.54***	0.62***	0.71***	0.70***	0.69***	0.03ns	0.20*
**Cu**	2019	−0.07 ns	0.75***	0.85***	0.85***	0.52***	0.50***	0.69***	0.77***	0.75***
	2020	0.22ns	0.99***	0.62***	0.60***	0.89***	0.83***	0.41***	0.35***	0.37***
**Fe**	2019	−0.04 ns	0.80***	0.87***	0.78***	0.71***	0.66***	0.63***	0.57***	0.52***
	2020	0.16ns	0.98***	0.89***	0.83***	0.65***	0.65***	0.40***	0.09ns	0.02ns
**K**	2019	0.26ns	0.93***	0.67***	0.65***	0.65***	0.63***	0.61***	0.32***	0.33***
	2020	0.51**	0.88***	0.81***	0.83***	0.79***	0.78***	0.58***	0.33***	0.41***
**Mg**	2019	0.14ns	0.85***	0.86***	0.83***	0.77***	0.75***	0.64***	0.53***	0.52***
	2020	0.54**	0.88***	0.52***	0.52***	0.66***	0.60***	0.65***	0.06ns	0.14ns
**Mn**	2019	0.29ns	0.96***	0.97***	0.97***	0.96***	0.95***	0.41***	0.35***	0.34***
	2020	0.22ns	0.98***	0.50***	0.50***	0.52***	0.52***	0.39***	0.09ns	0.13ns
**Na**	2019	0.03ns	0.75***	0.86***	0.81***	0.67***	0.63***	0.73***	0.63***	0.62***
	2020	0.34ns	0.91***	0.84***	0.78***	0.83***	0.79***	0.45***	0.30***	0.25**
**P**	2019	0.16ns	0.76***	0.75***	0.64***	0.64***	0.56***	0.78***	0.53***	0.46***
	2020	0.35ns	0.93***	0.43***	0.48***	0.50***	0.47***	0.57***	0.02ns	0.21*
**Zn**	2019	0.32ns	0.69***	0.90***	0.88***	0.71***	0.70***	0.76***	0.61***	0.59***
	2020	0.58**	0.88***	0.60***	0.52***	0.68***	0.59***	0.62***	0.13ns	0.11ns

Ca, K, Mg and Na (g kg^-1^); Cu, Fe, Mn and Zn (mg kg^-1^); ***, **, * = significantly different from zero at the 0.001, 0.01, and 0.05 probability levels, respectively. ns = not significantly different from zero at the 0.05 level of probability.

### Correlation of parents’ performance, hybrid phenotype, specific combining ability effects, and heterosis among leaf mineral elements

**Fig 4:**
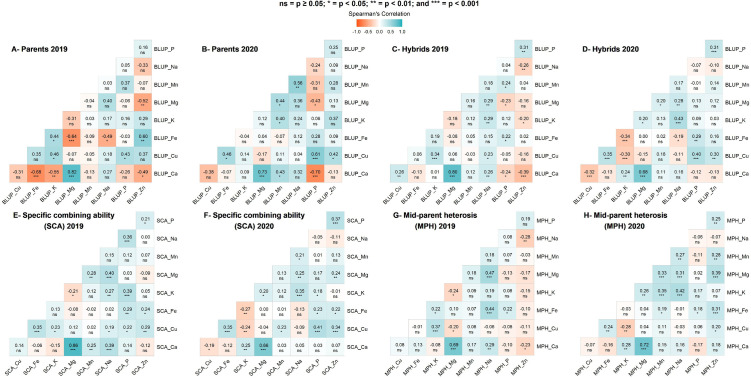
Correlations among leaf mineral elements based on the phenotypic performance for the 26 parental lines in 2019 (A) and 2020 (B), the phenotypic performance for the 118 hybrids in 2019 (C) and 2020 (D), specific combining ability effects for the 118 hybrids in 2019 (E) and 2020 (F) and mid-parent heterosis for the 118 hybrids in 2019 (G) and 2020 (H).

Variable and significant correlations among the leaf mineral contents for both parents and hybrids were observed, and the correlations ranged from weak to strong (Figs 4A–D). Despite some changes observed from 2019 to 2020 and from parents to hybrids, some leaf mineral content displayed similar correlation patterns in both years. Calcium and magnesium contents had a significant, positive, and strong correlation for both parents and hybrids (r ≥ 0.68, p < 0.001). A positive and significant association was observed between iron and phosphorus contents for both parents and hybrids. There was a moderate, positive, and significant correlation between phosphorus and copper contents (r ≥ 0.43, p < 0.05) for the parents. For hybrids, sodium content had a positive and significant correlation with potassium and magnesium contents. In addition, zinc content had a moderate and positive correlation with phosphorus content (r = 0.31, p < 0.01).

Significant and positive correlations were depicted among the specific combining ability (SCA) effects of the leaf mineral elements (Figs 4E–F). The SCA effect of magnesium had a strong, positive, and significant correlation with the SCA effect of calcium (r ≥ 0.66, p < 0.001). There was a positive and significant correlation between the SCA effects of iron and zinc (r ≥ 0.22, p < 0.05). Additionally, the SCA effect of iron had a positive and significant association with that of phosphorus (r ≥ 0.22, p < 0.05) and copper (r = 0.35, p < 0.001). Similarly, the SCA effect of zinc had a positive and significant association with the SCA effects of phosphorus (r ≥ 0.21, p < 0.05) and copper (r = 0.29, p < 0.01). There were significant and positive correlations between SCA effects of sodium and potassium (r ≥ 0.27, p < 0.01), copper and phosphorus (r ≥ 0.22, p < 0.05), magnesium and sodium (r ≥ 0.25, p < 0.01), and potassium and phosphorus (r ≥ 0.18, p < 0.05).

Overall, the correlations among heterosis in leaf mineral content varied significantly (Figs 4 G and H). We observed a significant, strong, and positive correlation between heterosis of calcium and magnesium (r ≥ 0.69, p < 0.001). There was also a positive, moderate, and significant correlation between the heterosis of magnesium and sodium (r ≥ 0.31, p < 0.001). Heterosis of iron had a positive and significant correlation with phosphorus but was weak (r < 0.30, p < 0.05).

## Discussion

In the present study, we observed highly significant variation among parents and F_1_ hybrids for leaf mineral content, showing the existence of functional genetic variation for spider plant improvement. The variation could be associated with the diversity in the origin of lines used in the present study, in line with reports by Sogbohossou et al. [11], Blalogoe et al. [62], and Reeves et al. [22]. Moreover, as observed in this study, Omondi et al. [12] reported significant variation in leaf mineral content among genotypes composed of farmers’ cultivars, advanced lines, and germplasm accessions from Eastern and Southern Africa. Similar observations for leaf mineral content were observed among Southern and Eastern genotypes [63,64] as well as among worldwide accessions in seed mineral composition [62], secondary metabolite concentrations [16], leaf vitamins content and morphological traits [11] and antioxidant activity [20]. More importantly, the genetic makeup of the genotypes has been reported to be associated with the phenotypic variation in *G. gynandra* [19,27].

Overall, hybrids outperformed their parents in several mineral contents, including zinc, potassium, phosphorus, and manganese, indicating hybrid vigor. Similar results were reported for mineral elements in *Brassica oleracea* var. *capitata* [38], in *Brassica rapa* [39], and in *Brassica oleracea* var. *botrytis* L. [40,41]. In addition, some hybrids were better or worse than their specific parents for various traits, showing that different mechanisms control the inheritance of the characteristics. The average performance of several hybrids for mineral content was higher than that of the parents and accessions previously reported by [12,14,63–66]. For instance, the average zinc content of 60 mg kg^-1^ dry weight (DW) with a maximum of 80 mg kg^-1^ DW reported by Omondi et al. [12] was lower than that of the present study, which was 73.19 mg kg^-1^ DW with a maximum of 224.95 mg kg^-1^ DW. The average Ca content in hybrids was twofold higher than that reported by Jiménez-Aguilar and Grusak [64]. However, we observed lower values for iron content than those of Moenga et al. [67], Omondi et al. [12], Gowele et al. [15], and Thovhogi et al. [63]. This might be due to the genotypes, the leaves sampling stage, cultivation practices, and growth conditions. For instance, Jinazali et al. [66] observed significant variation in mineral content between genotypes collected from different agroecological areas of Malawi. At the same time, Mamboleo et al. [68] and Makokha et al. [69] reported the effect of harvesting stage and growing locations on the leaf mineral content in *G. gynandra*. Further investigations are therefore required to assess the phenotypic plasticity and stability of the newly developed hybrids, identifying the best genotypes under various agroecological and agronomic practices in field conditions.

Knowledge of gene action and combining ability effects of traits is important for any crop breeding. Gene action is particularly crucial in the choice of breeding method [70]. Genetic variance component analysis revealed that both additive and non-additive genes influenced all traits. Nonadditive gene effects were predominant for all leaf mineral elements (Ca, Mg, K, P, Na, Fe, Zn, Cu, and Mn) in *G. gynandra*. This finding was supported by (1) the specific combining ability effects and dominance variances that were greater than the general combining ability effects and additive variances, respectively; (2) the degree of dominance greater than unity; and (3) the predictability ratio below 0.5 for all mineral contents. Consequently, the selection for nutritious cultivars in *G. gynandra* should focus on the development of hybrid cultivars for better exploitation of the dominance gene action through recurrent selection, especially reciprocal recurrent selection [70]. Similar findings were obtained by Xie et al. [39] for Ca, Fe, Mg, and Zn in *Brassica rapa* (Chinese cabbage), Singh et al. [71] for Fe, Zn, Cu, Mn, K and Ca in *Brassica oleracea* var. *capitata* (cabbage) where nonadditive gene action was predominant. Assessing epistasis was not part of the objectives of this study. Further studies using appropriate mating designs, such as North Carolina Design III and triple test cross should be implemented to estimate its contribution to the inheritance of mineral content in the species.

Heritability is a key parameter in breeding, particularly in the prediction of the response to selection. Broad-sense heritability (H^2^) is a measure of the proportion of the total phenotypic variation attributable to the variance of genetic values [72]. Broad-sense heritability estimates were high (> 0.60) for all mineral contents per year, showing that phenotypic variation observed among genotypes is primarily due to genotypic variation. Similar results were found concerning mineral content in the species [17] and *Amaranthus tricolor* L. [73]. Furthermore, narrow-sense heritability (h^2^) is a measure of the proportion of the total phenotypic variation attributable to additive genetic variance. A high h^2^ value indicates that phenotypic variation is primarily due to additive genetic effects. Thus, the higher the h^2^ value is, the better the response to selection will be. We observed low to moderate narrow-sense heritability for all mineral contents (< 50%), which agreed with the preponderance of dominance genes in the inheritance of mineral content in the species. Xie et al. [39] also reported low and moderate h^2^ for mineral content in *Brassica rapa*. In contrast, Karmakar et al. [74] reported low h^2^ for mineral and antioxidant content in ridge gourd (*Luffa acutangula* Roxb.).

Moreover, the genotype × year interaction was significant, with variance greater than the genotypic variance. This indicates phenotypic plasticity in the species. Similarly, considerable genotype by environment interaction effect was reported on leaf mineral content in the species [17], switchgrass tiller mineral content [75], rice seed elemental composition [76], maize kernel mineral concentration [77], seed iron and zinc content of sorghum [78] and common bean [79]. Moreover, the plant mineral composition is influenced by the crop genetics, the growing environment, the management practices, and their interaction [80,81]. The environment encompasses various factors, including soil conditions and climate, such as relative humidity, light intensity, and temperature, among others. Potential contributing factors to the observed genotype × year interaction might include soil type, temperature, and relative humidity. Soil could be the main driving factor, as plants absorb nutrients from the soil, and different types were used in this study, with soil in 2019 and growing media in 2020. However, this requires further investigation by evaluating these parents and progenies in multiple environments to confirm the level of environmental influence and genotype-by-environment interactions in phenotypic variation, and to identify key loci controlling leaf mineral content through ecological interaction, as reported in various species, including rice, maize, and switchgrass [75–77].

The expected genetic gain, another important metric for breeding for quantitative traits, estimates the quantity of increase in performance between the selected and base populations and is key, along with heritability, in any breeding program [82]. Our expected genetic gain at a selection intensity of 5% for all the mineral content ranged between 21.93% and 95.90%. A genetic gain of over 20% was observed for all minerals, indicating that significant improvements would be achieved through selection.

General combining ability is crucial for parental selection, while specific combining ability is essential for optimal cross selection to harness heterosis. Although nonadditive gene effects were predominant, high and significant general combining ability effects were observed for some female and/or male parents in terms of mineral contents, and these effects were mainly due to additive and additive × additive gene effects [83]. The parents with good GCA effects are excellent founders for the development of improved populations and could be exploited through several generations of hybridization. In the present study, neither male nor female parents simultaneously showed significant GCA effects in the desired direction for all mineral contents. This result concurs with previous findings concerning minerals in cabbage head [71] and minerals, vitamins and antioxidants in cauliflower [40,83]. However, some females (P12, P09, P11, P10, P05) and males (P25, P26, P15, P24) parents are suitable for their multiple positive and significant GCA effects. These parents are excellent and valuable candidates and resources for developing improved populations for research and breeding purposes.

Specific combining ability effects result from nonadditive gene effects, comprising dominance and epistasis [34]. In this study, none of the crosses displayed high and significant SCA effects for all minerals. This finding is similar to the results of Parkash et al. [84] for antioxidant compounds in *Brassica oleracea* var. *capitata*. Still, it contrasts with those of Singh et al. [71] and Xie et al. [39]. The latter researchers were able to identify at least one cross with significant and positive SCA effects for all the investigated minerals in cabbage head and non-heading Chinese cabbage. However, depending on the targeted mineral, hybrids with the highest and most significant SCA in the desirable direction involved (i) both parents with good and significant GCA effects (e.g., P12 × P25 for Mg, P09 × P24 for K, P09 × P19 for Fe); (ii) one good and one poor combiner (e.g., P05 × P25 for Ca, P09 × P21 for Mn, P10 × P18 for K); and (iii) both parents with medium or bad GCA effects (e.g., P12 × P13 for Mn, P02 × P15 for Mg, P09 × P18 for Zn). This finding shows that depending on the trait, the observed SCA effect might result from (i) the cumulative effects of additive genes (good x good parents); (ii) the interaction between additive and nonadditive genes (good x poor general combiners or vice versa); and (iii) the over manifestation of the interaction between nonadditive genes, especially complementary epistatic effects [34,39,71,83,85]. We also observed that some crosses involving both parents with good GCA displayed significant and negative SCA effects. This might be the result of the absence of or weak interaction between the desirable alleles. Therefore, crosses from good general combiners might not always display desirable SCA effects. Based on the above, breeding strategies for high-quality leaves in *G. gynandra* should consider both GCA and SCA in the selection of superior parents and crosses. Heterosis breeding and recurrent selection, along with multiple crossing programs, can be implemented. Types of cultivars may include hybrids, synthetics, composites, and population improvements. Strategies implemented for allogamous species can also be applied to this species. Therefore, breeding strategies should focus on (i) selecting parents with good general combining ability, followed by (ii) a selection based on specific combining ability. Reciprocal recurrent selection would be the most effective method for exploiting both additive and non-additive gene action in the species.

Heterosis, also known as hybrid vigor, refers to the outperformance of F1 progeny over their parents and has significantly contributed to increased crop productivity. Here, we report for the first time this phenomenon in *G. gynandra*. The level of heterosis over the mid and best parents was significant and variable between leaf mineral concentrations. This agrees with earlier reports on the existence of heterosis for mineral content, vitamins, antioxidants, and proteins in vegetable crops such as cabbage, tomato, cauliflower, pea and bean [38,39,83,86–90]. Specifically, the level of heterosis observed in the present study was higher than that reported for minerals in non-heading Chinese cabbage [39] and *Brassica oleracea* var. *capitata* [38]. The result was comparable to the level of heterosis for vitamins and antioxidant pigments in cauliflower [83] and cabbage [84] and some bioactive properties in interspecific crosses between cultivated and wild relatives of eggplant [91]. The wide range of heterosis could be explained by the previous observations on the reproductive biology of the species, revealing that the species is predominantly outcrossing [25–27].

Both negative and positive mid- and best parent heterosis were observed in the species for all mineral content. This might be because several mechanisms are underlying heterosis expression in *G. gynandra*. Three main models have been widely used to explain heterosis in crops, including dominance, overdominance, and epistasis [29,92–94]. Moreover, hybrids exhibiting a high level of heterosis are a combination of parents with either both good, both poor, average x good, good x poor, average x average, or average x poor general combining abilities. The results showed that all three models or their combination could explain heterosis in *G. gynandra,* as most research has highlighted that a single model rarely occurs in plants [92,95]. The present observation of the existence of heterosis in *G. gynandra* adds to previous reports [7,96] that the species could be used as a model for heterosis studies. A good exploitation of heterosis in *G. gynandra* requires the identification of heterotic patterns and groups in the species. To this end, the observed genetic differentiation between accessions based on geographical origin is key, and further investigation to assess the cross-compatibility between them, as well as within each region, is essential to avoid possible incompatibility between accessions. Additionally, identifying familiar testers will help expedite the exploitation of heterosis in the species.

Both additive and nonadditive gene action are controlling minerals, with a predominance of nonadditive genes in *G. gynandra*. More importantly, we observed a positive association between F_1_ performance and SCA, the sum of both parents’ GCA effects and F_1_ performance, showing that the prediction of F_1_ performance in spider plant should be based on models involving both GCA and SCA. Specifically, the correlations between F_1_ performance and SCA and heterosis were strong. Similarly, a more substantial, significant, and positive correlation between SCA and heterosis (MPH and BPH) was reported for agronomic traits in *Brassica oleracea* [85]. The SCA was, therefore, the major driver of hybrid performance and heterosis for leaf mineral content in spider plant and should be considered in selecting parents or populations for improvement. This opens the door to investigating genomic selection methods and machine learning techniques for predicting hybrid performance in the species.

This study revealed that hybridization represents an important breeding strategy for improving the nutritional values of *G. gynandra*. This is attributable to the observed high heterosis, the superiority of specific combining ability over the general combining ability, and the dominance gene action for all leaf mineral element contents investigated (zinc, copper, manganese, calcium, magnesium, sodium, phosphorus, and potassium).

Giving the current global inadequate intakes of calcium (66% of the world population with 5 billion people) and magnesium (31% of the world population with 2.4 billion people) [18] and the high concentration of calcium and magnesium in the leaves of spider plant, the regular consumption of the leaves of spider plant will significantly contribute to combatting calcium and magnesium deficiencies [17]. The consistency of the positive and strong correlation between calcium and magnesium (r ≥ 0.66, p < 0.001) for their concentrations in the leaves of the parents and hybrids, specific combining ability effects, and heterosis, showed that simultaneous selection could be undertaken. Therefore, improving calcium content could result in enhancing magnesium content and could be *the top mineral breeding priority* in *G. gynandra*. A strong correlation between calcium and magnesium concentrations has been previously reported in the species [17,63] and in the leaves of *Brassica napus* L. [97], in shoots of *Brassica oleracea* L. [98]. Possibly, a strong linkage might exist between genes involved in the accumulation of magnesium and calcium in the leaves of the spider plant, as reported in *Brassica* species [97,98]. For instance, Broadley et al. [98] reported that shoot calcium and magnesium contents in *B. oleracea* are heritable and controlled by pleiotropic loci. Similarly, Alcock et al. [97] also identified loci associated with both calcium and magnesium concentrations in leaves of *B. napus*, which are colocalized in the same regions of the chromosomes. Further studies could investigate the genomic regions controlling the calcium and magnesium accumulation in the leaves of *G. gynandra*. Moreover, this strong association could be explained by the antagonistic interaction between calcium and magnesium in plant cells [99,100]. Consequently, a homeostatic balance between calcium and magnesium ions are important for plant development and growth [99,101]. While calcium play a crucial role for cytosolic signalling, membrane and cell wall integrity [102], magnesium is a key constituent of chlorophyll, essential for photosynthesis, cofactor for enzymes, protein synthesis and energy metabolism [103]. Though calcium and magnesium have different biochemical and physiological roles in plants, homeostasis of calcium and magnesium ions appeared to be closely linked and at least partly regulated by common signaling networks [99]. Specifically, Ca^2+^ signaling participate in regulating the dynamic homeostasis of Mg^2+^ [99]. Therefore, it was suggested that high external magnesium ions would lead to a transient rise in cytosolic calcium ions in plant cells. In case of excess magnesium, the Ca^2+^ signal is detected by two tonoplast-localized calcineurin B-like (CBL) proteins (CBL2 and CBL3), which concomitantly activate four of CBL-interacting protein kinases (CIPKs) (CIPK3, 9, 23 and 26). This CBL–CIPK complex, therefore, recruits Mg^2+^ and regulate downstream specific transporters in the tonoplast for efficient sequestration of Mg^2+^ in vacuoles, thereby ensuring a non-toxic level of Mg^2+^ in the cell as a protective mechanism [104]. This opens rooms for the use of *G. gynandra* to uncover the physiological and regulatory mechanism of calcium and magnesium hemostasis in plants. Furthermore, improving calcium and magnesium content might lead to increased sodium and potassium content, as positive associations were observed between magnesium and sodium for heterosis, SCA effects, and leaf content in hybrids, and between sodium and potassium for SCA effects and hybrids’ leaf content.

Breeding for improved iron content in spider plant leaves could be the *second top breeding* priority. Iron is an essential micronutrient and represents the most deficient as its inadequate intake affects about 4.9 billion people, 65% of the world population [18] specifically children and expectant women. Improving the iron content in the leaves of the spider plant could lead to an increase in zinc content (another essential and deficient nutrient affecting 3.5 billion people, or 46% of the world’s population) through the indirect effect of phosphorus. Phosphorus and iron were positively correlated in the content of leaves from the parents and hybrids, as well as in specific combining ability effects and heterosis. In addition, phosphorus had a positive association with zinc for their content in the leaves of the hybrids as well as for specific combining ability effects. A positive correlation was previously reported between iron and zinc content in the species, with zinc having a positive direct effect on iron content [17]. Breeding that takes into account the SCA effect of iron could improve the zinc, copper, and phosphorus content, as positive associations were observed between their SCA effects.

As this study pointed out that hybrid cultivars will help improve the species productivity, the establishment of hybrid breeding will be beneficial. However, this might face some challenges, such as the cost-effectiveness of the breeding program and the willingness of farmers to buy hybrid seeds as hybrids seeds are often expensive compared with pure lines or open pollinated cultivars. Given the competitive nutritional advantages of *G. gynandra* over some world leading *Brassica* vegetables [2], a proper advocacy and rising awareness could boost the species demand across Africa and the globe. The success of the development of hybrid cultivars for spider plant will require (i) an investigation of male sterility to ease the hybrid production, (ii) the evaluation of the cost-effectiveness of hybrid cultivars and (iii) the establishment of seed production logistics as vegetable seed system in Africa is still dominated by informal system. Despite these challenges, experience from Brassicaceae species, a sister family to Cleomaceae family, which *G. gynandra* belongs to could be useful. The implementation of hybrid breeding program should consider a holistic approach that consider all stakeholders along the crop value chains. Therefore, actions could include: (i) the evaluation of best parents and crosses for agronomic traits and adaptability though participatory breeding and tricot citizen science taking into account social factors and networks; (ii) farmers training on good agronomic practices; (iii) the development of seed system to ensure permanent seed availability; and (iv) the assessment of market acceptability, taste and sensory attributes. At the same time, raising awareness and knowledge sharing of nutritional and health benefits of the species activities should be implemented to stimulate demand for market creation.

## Conclusion

The present study has generated critical and novel insights into the genetic mechanisms governing the inheritance of mineral content in *G. gynandra*. We observed significant variation in mineral content among parents and hybrids. Genetic variance components analysis revealed significant effects of both general and specific combining ability, indicating the action of both additive and nonadditive genes, with the predominance of nonadditive gene action in the inheritance of mineral content in the species. The degrees of dominance observed varied depending on the trait, ranging from dominance to overdominance. Our results also revealed the presence of both negative and positive mid- and best-parent heterosis for leaf mineral content in the species. High broad-sense and low to moderate narrow-sense heritability estimates were observed for all minerals. Significant genetic gain was obtained for all mineral contents at a selection pressure of 5%. The best crosses resulted from different parental combinations, ranging from good to poor combiners, suggesting that selection should be based on both general and specific combining ability effects. Heterosis breeding and reciprocal recurrent selection are ideal breeding strategies for developing mineral-dense cultivars that enhance nutrition. Several cultivars can be developed, including hybrids, open-pollinated varieties, and synthetic varieties. We therefore suggest using *G. gynandra* as a model crop for investigating the mechanisms underlying heterosis in plants. Further research on heterotic groups and patterns, as well as tester identification, is needed to fully exploit heterosis in the species. Overall, parents with good combining ability (P05, P09, P10, P11, P12, P15, P24, P25, P26) and crosses expressing promising hybrid vigor (P10 × P13, P01 × P21, P09 × P19, P10 × P14) were identified and represent resources for breeding and research purposes.

## Supporting information

S1 Table Representation of the North Carolina Design II implemented to generate the 118 F1 hybrids used in the present study. (DOCX)

S2 Table Phenotypic values of leaf minerals content of 118 experimental hybrids and their 26 parents of *Gynandropsis gynandra* evaluated in 2019 and 2020. (XLSX)

S1 Fig Variations in temperature, relative humidity and solar radiation under the greenhouse in 2019 and 2020. (A) Temperature in 2019. (B) Temperature in 2020. (C) Relative humidity in 2019. (D) Relative humidity in 2020. (E) Solar radiation in 2019. (F) Solar radiation in 2020.
(TIF)

S2 Fig Estimates of specific combining ability (SCA) effects of calcium concentration for 118 experimental hybrids of *G. gynandra* evaluated in 2019 and 2020. ***, **, * refer to estimate of specific combining ability effect significantly different from zero at p < 0.001, 0.01 and 0.05, respectively.
(TIF)

S3 Fig Estimates of specific combining ability (SCA) effects of magnesium concentration for 118 experimental hybrids of *G. gynandra* evaluated in 2019 and 2020. ***, **, * refer to estimate of specific combining ability effect significantly different from zero at p < 0.001, 0.01 and 0.05, respectively.
(TIF)

S4 Fig Estimates of specific combining ability (SCA) effects of phosphorus concentration for 118 experimental hybrids of *G. gynandra* evaluated in 2019 and 2020. ***, **, * refer to estimate of specific combining ability effect significantly different from zero at p < 0.001, 0.01 and 0.05, respectively.
(TIF)

S5 Fig Estimates of specific combining ability (SCA) effects of potassium concentration for 118 experimental hybrids of *G. gynandra* evaluated in 2019 and 2020. ***, **, * refer to estimate of specific combining ability effect significantly different from zero at p < 0.001, 0.01 and 0.05, respectively.
(TIF)

S6 Fig Estimates of specific combining ability (SCA) effects of sodium concentration for 118 experimental hybrids of *G. gynandra* evaluated in 2019 and 2020. ***, **, * refer to estimate of specific combining ability effect significantly different from zero at p < 0.001, 0.01 and 0.05, respectively.
(TIF)

S7 Fig Estimates of specific combining ability (SCA) effects of iron concentration for 118 experimental hybrids of *G. gynandra* evaluated in 2019 and 2020. ***, **, * refer to estimate of specific combining ability effect significantly different from zero at p < 0.001, 0.01 and 0.05, respectively.
(TIF)

S8 Fig Estimates of specific combining ability (SCA) effects of copper concentration for 118 experimental hybrids of *G. gynandra* evaluated in 2019 and 2020. ***, **, * refer to estimate of specific combining ability effect significantly different from zero at p < 0.001, 0.01 and 0.05, respectively.
(TIF)

S9 Fig Estimates of specific combining ability (SCA) effects of manganese concentration for 118 experimental hybrids of *G. gynandra* evaluated in 2019 and 2020. ***, **, * refer to estimate of specific combining ability effect significantly different from zero at p < 0.001, 0.01 and 0.05, respectively.
(TIF)

S10 Fig Estimates of specific combining ability (SCA) effects of zinc concentration for 118 experimental hybrids of *G. gynandra* evaluated in 2019 and 2020. ***, **, * refer to estimate of specific combining ability effect significantly different from zero at p < 0.001, 0.01 and 0.05, respectively.
(TIF)

S11 Fig Estimates of mid parent heterosis of calcium concentration for 118 experimental hybrids of *G. gynandra* evaluated in 2019 and 2020. (TIF)

S12 Fig Estimates of best parent heterosis of calcium concentration for 118 experimental hybrids of *G. gynandra* evaluated in 2019 and 2020. (TIF)

S13 Fig Estimates of mid parent heterosis of magnesium concentration for 118 experimental hybrids of *G. gynandra* evaluated in 2019 and 2020. (TIF)

S14 Fig Estimates of best parent heterosis of magnesium concentration for 118 experimental hybrids of *G. gynandra* evaluated in 2019 and 2020. (TIF)

S15 Fig Estimates of mid parent heterosis of phosphorus concentration for 118 experimental hybrids of *G. gynandra* evaluated in 2019 and 2020. (TIF)

S16 Fig Estimates of best parent heterosis of phosphorus concentration for 118 experimental hybrids of *G. gynandra* evaluated in 2019 and 2020. (TIF)

S17 Fig Estimates of mid parent heterosis of potassium concentration for 118 experimental hybrids of *G. gynandra* evaluated in 2019 and 2020. (TIF)

S18 Fig Estimates of best parent heterosis of potassium concentration for 118 experimental hybrids of *G. gynandra* evaluated in 2019 and 2020. (TIF)

S19 Fig Estimates of mid parent heterosis of sodium concentration for 118 experimental hybrids of *G. gynandra* evaluated in 2019 and 2020. (TIF)

S20 Fig Estimates of best parent heterosis of sodium concentration for 118 experimental hybrids of *G. gynandra* evaluated in 2019 and 2020. (TIF)

S21 Fig Estimates of mid parent heterosis of iron concentration for 118 experimental hybrids of *G. gynandra* evaluated in 2019 and 2020. (TIF)

S22 Fig Estimates of best parent heterosis of iron concentration for 118 experimental hybrids of *G. gynandra* evaluated in 2019 and 2020. (TIF)

S23 Fig Estimates of mid parent heterosis of copper concentration for 118 experimental hybrids of *G. gynandra* evaluated in 2019 and 2020. (TIF)

S24 Fig Estimates of best parent heterosis of copper concentration for 118 experimental hybrids of *G. gynandra* evaluated in 2019 and 2020. (TIF)

S25 Fig Estimates of mid parent heterosis of manganese concentration for 118 experimental hybrids of *G. gynandra* evaluated in 2019 and 2020. (TIF)

S26 Fig Estimates of best parent heterosis of manganese concentration for 118 experimental hybrids of *G. gynandra* evaluated in 2019 and 2020. (TIF)

S27 Fig Estimates of mid parent heterosis of zinc concentration for 118 experimental hybrids of *G. gynandra* evaluated in 2019 and 2020. (TIF)

S28 Fig Estimates of best parent heterosis of zinc concentration for 118 experimental hybrids of *G. gynandra* evaluated in 2019 and 2020. (TIF)

## References

[1] Nyadanu D, Lowor ST. Promoting competitiveness of neglected and underutilized crop species: comparative analysis of nutritional composition of indigenous and exotic leafy and fruit vegetables in Ghana. Genet Resour Crop Evol. 2014;62(1):131–40. doi: 10.1007/s10722-014-0162-x

[2] Moyo M, Amoo SO, Aremu AO, Gruz J, Šubrtová M, Jarošová M, et al. Determination of Mineral Constituents, Phytochemicals and Antioxidant Qualities of *Cleome gynandra*, Compared to *Brassica oleracea* and *Beta vulgaris*. Front Chem. 2018;5:128. doi: 10.3389/fchem.2017.00128 29354633 PMC5758552

[3] Orech FO, Christensen DL, Larsen T, Friis H, Aagaard-Hansen J, Estambale BA. Mineral content of traditional leafy vegetables from western Kenya. Int J Food Sci Nutr. 2007;58(8):595–602. doi: 10.1080/09637480701350288 17852510

[4] van Zonneveld M, Kindt R, McMullin S, Achigan-Dako EG, N’Danikou S, Hsieh W-H, et al. Forgotten food crops in sub-Saharan Africa for healthy diets in a changing climate. Proc Natl Acad Sci U S A. 2023;120(14):e2205794120. doi: 10.1073/pnas.2205794120 36972432 PMC10083591

[5] Jamnadass R, Mumm RH, Hale I, Hendre P, Muchugi A, Dawson IK, et al. Enhancing African orphan crops with genomics. Nat Genet. 2020;52(4):356–60. doi: 10.1038/s41588-020-0601-x 32203464

[6] Karl K, MacCarthy D, Porciello J, Chimwaza G, Fredenberg E, Freduah BS. Opportunity Crop Profiles for the Vision for Adapted Crops and Soils (VACS) in Africa. 2024.

[7] Sogbohossou EOD, Achigan-Dako EG, Maundu P, Solberg S, Deguenon EMS, Mumm RH, et al. A roadmap for breeding orphan leafy vegetable species: a case study of *Gynandropsis gynandra* (Cleomaceae). Hortic Res. 2018;5:2. doi: 10.1038/s41438-017-0001-2 29423232 PMC5798814

[8] Marles RJ. Mineral nutrient composition of vegetables, fruits and grains: The context of reports of apparent historical declines. Journal of Food Composition and Analysis. 2017;56:93–103. doi: 10.1016/j.jfca.2016.11.012

[9] Mayer A-MB, Trenchard L, Rayns F. Historical changes in the mineral content of fruit and vegetables in the UK from 1940 to 2019: a concern for human nutrition and agriculture. Int J Food Sci Nutr. 2022;73(3):315–26. doi: 10.1080/09637486.2021.1981831 34651542

[10] Davis DR. Declining Fruit and Vegetable Nutrient Composition: What Is the Evidence?. horts. 2009;44(1):15–9. doi: 10.21273/hortsci.44.1.15

[11] Sogbohossou EOD, Kortekaas D, Achigan-Dako EG, Maundu P, Stoilova T, Van Deynze A, et al. Association between vitamin content, plant morphology and geographical origin in a worldwide collection of the orphan crop *Gynandropsis gynandra* (Cleomaceae). Planta. 2019;250(3):933–47. doi: 10.1007/s00425-019-03142-1 30911886

[12] Omondi EO, Engels C, Nambafu G, Schreiner M, Neugart S, Abukutsa-Onyango M, et al. Nutritional compound analysis and morphological characterization of spider plant (*Cleome gynandra*) - an African indigenous leafy vegetable. Food Res Int. 2017;100(Pt 1):284–95. doi: 10.1016/j.foodres.2017.06.050 28873690

[13] Neugart S, Baldermann S, Ngwene B, Wesonga J, Schreiner M. Indigenous leafy vegetables of Eastern Africa - A source of extraordinary secondary plant metabolites. Food Res Int. 2017;100(Pt 3):411–22. doi: 10.1016/j.foodres.2017.02.014 28964364

[14] Schönfeldt HC, Pretorius B. The nutrient content of five traditional South African dark green leafy vegetables—A preliminary study. Journal of Food Composition and Analysis. 2011;24(8):1141–6. doi: 10.1016/j.jfca.2011.04.004

[15] Gowele VF, Kinabo J, Jumbe T, Kirschmann C, Frank J, Stuetz W. Provitamin A Carotenoids, Tocopherols, Ascorbic Acid and Minerals in Indigenous Leafy Vegetables from Tanzania. Foods. 2019;8(1):35. doi: 10.3390/foods8010035 30669425 PMC6352189

[16] Sogbohossou EOD, Achigan-Dako EG, Mumm R, de Vos RCH, Schranz ME. Natural variation in specialised metabolites production in the leafy vegetable spider plant (*Gynandropsis gynandra* L. (Briq.)) in Africa and Asia. Phytochemistry. 2020;178:112468. doi: 10.1016/j.phytochem.2020.112468 32771676

[17] Houdegbe AC, Achigan-Dako EG, Sogbohossou EOD, Schranz ME, Odindo AO, Sibiya J. Leaf elemental composition analysis in spider plant [*Gynandropsis gynandra* L. (Briq.)] differentiates three nutritional groups. Front Plant Sci. 2022;13:841226. doi: 10.3389/fpls.2022.841226 36119621 PMC9478508

[18] Passarelli S, Free CM, Shepon A, Beal T, Batis C, Golden CD. Global estimation of dietary micronutrient inadequacies: a modelling analysis. Lancet Glob Health. 2024;12(10):e1590–9. doi: 10.1016/S2214-109X(24)00276-6 39218000 PMC11426101

[19] Sogbohossou DEO. Orphan no more: Ethnobotany and genetic analysis of leaf yield and secondary metabolites content in *Gynandropsis gynandra* (Cleomaceae). Wageningen: Wageningen University. 2019.

[20] Chataika BY, Akundabweni LS-M, Houdegbe AC, Achigan-Dako EG, Sibiya J, Masamba K. Dietary Phytochemical Screening of Spider Plant (*Gynandropsis gynandra* (L.) Briq.) Accessions From Africa and Asia to Identify Genotypes for Use in Nutraceutical Breeding. Front Sustain Food Syst. 2021;5. doi: 10.3389/fsufs.2021.733909

[21] Mashamaite CV, Manyevere A, Chakauya E. *Cleome gynandra*: A wonder climate-smart plant for nutritional security for millions in semi-arid areas. Front Plant Sci. 2022;13:1003080. doi: 10.3389/fpls.2022.1003080 36212376 PMC9538671

[22] Reeves G, Singh P, Rossberg TA, Sogbohossou EOD, Schranz ME, Hibberd JM. Natural Variation within a Species for Traits Underpinning C4 Photosynthesis. Plant Physiol. 2018;177(2):504–12. doi: 10.1104/pp.18.00168 29678862 PMC6001323

[23] Hoang NV, Sogbohossou EOD, Xiong W, Simpson CJC, Singh P, Walden N, et al. The *Gynandropsis gynandra* genome provides insights into whole-genome duplications and the evolution of C4 photosynthesis in Cleomaceae. Plant Cell. 2023;35(5):1334–59. doi: 10.1093/plcell/koad018 36691724 PMC10118270

[24] Singh P, Stevenson SR, Dickinson PJ, Reyna-Llorens I, Tripathi A, Reeves G, et al. C4 gene induction during de-etiolation evolved through changes in cis to allow integration with ancestral C3 gene regulatory networks. Sci Adv. 2023;9(13):eade9756. doi: 10.1126/sciadv.ade9756 36989352 PMC10058240

[25] Zohoungbogbo HPF, Houdegbe CA, Sogbohossou DEO, Tossou MG, Maundu P, Schranz EM, et al. Andromonoecy in *Gynandropsis gynandra* (L.) Briq. (Cleomaceae) and effects on fruit and seed production. Genet Resour Crop Evol. 2018;65(8):2231–9. doi: 10.1007/s10722-018-0687-5

[26] Raju AS, Rani DS. Reproductive ecology of *Cleome gynandra* and *Cleome viscosa* (Capparaceae). Phytol Balcan. 2016;22(1):15–28.

[27] Omondi EO, Debener T, Linde M, Abukutsa‐Onyango M, Dinssa FF, Winkelmann T. Mating biology, nuclear DNA content and genetic diversity in spider plant (*Cleome gynandra*) germplasm from various African countries. Plant Breeding. 2017;136(4):578–89. doi: 10.1111/pbr.12485

[28] Shull GH. The Composition of a Field of Maize. Journal of Heredity. 1908;os-4(1):296–301. doi: 10.1093/jhered/os-4.1.296

[29] Hochholdinger F, Baldauf JA. Heterosis in plants. Curr Biol. 2018;28(18):R1089–92. doi: 10.1016/j.cub.2018.06.041 30253145

[30] Darwin C. The effects of cross and self fertilisation in the vegetable kingdom. D. Appleton. 1877.

[31] Shull GH. What is “heterosis”?. Genetics. 1948;33(5):439. 17247290 10.1093/genetics/33.5.439 PMC1209417

[32] Fehr WR, Suza WP. Principles of cultivar development. Ames, Iowa, United States of America: Iowa State University Digital Press. 2025.

[33] Houdegbe AC, Achigan-Dako EG, Sogbohossou EOD, Schranz ME, Odindo AO, Sibiya J. Phenotypic variation in biomass and related traits among four generations advanced lines of Cleome (*Gynandropsis gynandra* L. (Briq.)). PLoS One. 2022;17(10):e0275829. doi: 10.1371/journal.pone.0275829 36223403 PMC9555646

[34] Sprague GF, Tatum LA. General vs. Specific Combining Ability in Single Crosses of Corn1. Agronomy Journal. 1942;34(10):923–32. doi: 10.2134/agronj1942.00021962003400100008x

[35] Nduwumuremyi A, Tongoona P, Habimana S. Mating designs: helpful tool for quantitative plant breeding analysis. J Plant Breed Genet. 2013;1(3):117–29.

[36] Beavis W, Lamkey K, Mahama AA, Suza W. Mating Designs. In: Suza WP, Lamkey KR. Quantitative Genetics for Plant Breeding. Iowa State University Digital Press. 2023.

[37] Hallauer AR, Carena MJ, Miranda Filho J d. Quantitative genetics in maize breeding. London: Springer Science & Business Media. 2010.

[38] Singh BK, Sharma SR, Singh B. Heterosis for mineral elements in single cross-hybrids of cabbage (*Brassica oleracea* var. *capitata* L.). Scientia Horticulturae. 2009;122(1):32–6. doi: 10.1016/j.scienta.2009.04.007

[39] Xie F, Zha J, Tang H, Xu Y, Liu X, Wan Z. Combining ability and heterosis analysis for mineral elements by using cytoplasmic male-sterile systems in non-heading Chinese cabbage (*Brassica rapa*). Crop Pasture Sci. 2018;69(3):296. doi: 10.1071/cp17357

[40] Ram H, Dey SS, Gopala Krishnan S, Kar A, Bhardwaj R, Arun Kumar MB, et al. Heterosis and Combining Ability for Mineral Nutrients in Snowball Cauliflower (*Brassica oleracea* var. *botrytis* L.) Using Ogura Cytoplasmic Male Sterile Lines. Proc Natl Acad Sci, India, Sect B Biol Sci. 2017;88(4):1367–76. doi: 10.1007/s40011-017-0874-8

[41] Karigar GP, Singh S, Mangal M, Saroha S, Saini N, Ray M, et al. Dietary micronutrient content, heterosis and combining ability for breeding mineral-rich hybrids in early- and mid-maturity groups of Indian cauliflower. Scientia Horticulturae. 2023;312:111848. doi: 10.1016/j.scienta.2023.111848

[42] Zohoungbogbo HPF, Blalogoe JS, Sogbohossou EDO, Houdegbe CA, Achigan-Dako EG. Tips for successful crosses in Cleome. Cotonou, Benin: Laboratory of Genetics Horticulture and Seed Science. 2018.

[43] Jones JB. Laboratory guide for conducting soil tests and plant analysis. Boca Raton, Florida, USA: CRC press. 2001.

[44] Clesceri LS, Greenberg AE, Eaton AD. 4500-P Phosporus. Standard Methods for the Examination of Water and Wastewater. Washington, DC: American Public Health Association, American Water Works Association, Water Environment Federation. 1999. 4-146-4–162.

[45] Kalra Y. Handbook of Reference Methods for Plant Analysis. Boca Raton, Florida, USA: CRC Press. 1997.

[46] Hunter A. New techniques and equipment for routine soil/plant analytical procedures. In: Borremiza E, Alvarado A. Soil management in Tropical America. Raleigh, NC: N.C. State University. 1975.

[47] Manson A, Bainbridge S, Thibaud G. s7. Pietermaritzburg, South Africa: KwaZulu-Natal Department of Agriculture and Rural Development. 2020.

[48] R Core Team. R: A language and environment for statistical computing. Vienna, Austria: R Foundation for Statistical Computing. 2024.

[49] Bernal-Vasquez A-M, Utz H-F, Piepho H-P. Outlier detection methods for generalized lattices: a case study on the transition from ANOVA to REML. Theor Appl Genet. 2016;129(4):787–804. doi: 10.1007/s00122-016-2666-6 26883044

[50] Revelle W. Psych: procedures for psychological, psychometric, and personality research. 1.9.12 ed. Evanston, Illinois: Northwestern University. 2019.

[51] Butler D, Cullis B, Gilmour A, Gogel B, Thompson R. ASReml-R Reference Manual Version 4. Hemel Hempstead, HP1 1ES: VSN International Ltd. 2017.

[52] Self SG, Liang K-Y. Asymptotic Properties of Maximum Likelihood Estimators and Likelihood Ratio Tests under Nonstandard Conditions. Journal of the American Statistical Association. 1987;82(398):605–10. doi: 10.1080/01621459.1987.10478472

[53] Isik F, Holland J, Maltecca C. Genetic data analysis for plant and animal breeding. Springer. 2017.

[54] Dhillon BS. Estimation of Degree of Dominance and Gene Frequency in Random Mating Populations. Biometrical J. 1990;32(4):503–12. doi: 10.1002/bimj.4710320414

[55] Piepho HP, Möhring J, Melchinger AE, Büchse A. BLUP for phenotypic selection in plant breeding and variety testing. Euphytica. 2007;161(1–2):209–28. doi: 10.1007/s10681-007-9449-8

[56] Molenaar H, Boehm R, Piepho H-P. Phenotypic Selection in Ornamental Breeding: It’s Better to Have the BLUPs Than to Have the BLUEs. Front Plant Sci. 2018;9:1511. doi: 10.3389/fpls.2018.01511 30455707 PMC6230591

[57] Kleinknecht K, Möhring J, Singh KP, Zaidi PH, Atlin GN, Piepho HP. Comparison of the Performance of Best Linear Unbiased Estimation and Best Linear Unbiased Prediction of Genotype Effects from Zoned Indian Maize Data. Crop Science. 2013;53(4):1384–91. doi: 10.2135/cropsci2013.02.0073

[58] Dabholkar A. Elements of Bio Metrical Genetics. revised and enlarged edition ed. New Delhi, India: Concept Publishing Company. 1999.

[59] Baker RJ. Issues in Diallel Analysis. Crop Science. 1978;18(4):533–6. doi: 10.2135/cropsci1978.0011183x001800040001x

[60] Singh RK, Chaudhary BD. Biometrical methods in quantitative genetic analysis. Third ed. New Delhi, India: Kalyani Publishers. 1985.

[61] Harrell Jr FE, Dupont C. Hmisc: Harrell Miscellaneous. 2021.

[62] Blalogoe JS, Odindo AO, Sogbohossou EOD, Sibiya J, Achigan-Dako EG. Origin-dependence of variation in seed morphology, mineral composition and germination percentage in *Gynandropsis gynandra* (L.) Briq. accessions from Africa and Asia. BMC Plant Biol. 2020;20(1):168. doi: 10.1186/s12870-020-02364-w 32295516 PMC7160957

[63] Thovhogi F, Mchau GRA, Gwata ET, Ntushelo N. Evaluation of Leaf Mineral, Flavonoid, and Total Phenolic Content in Spider Plant Germplasm. Molecules. 2021;26(12):3600. doi: 10.3390/molecules26123600 34208409 PMC8231248

[64] Jiménez-Aguilar DM, Grusak MA. Evaluation of Minerals, Phytochemical Compounds and Antioxidant Activity of Mexican, Central American, and African Green Leafy Vegetables. Plant Foods Hum Nutr. 2015;70(4):357–64. doi: 10.1007/s11130-015-0512-7 26490448

[65] Glew RS, Amoako-Atta B, Ankar-Brewoo G, Presley J, Chuang LT, Millson M, et al. Non-cultivated plant foods in West Africa: Nutritional analysis of the leaves of three indigenous leafy vegetables in Ghana. Food. 2009;3(1):39–42.

[66] Jinazali H, Mtimuni B, Chilembwe E. Nutrient composition of cats whiskers (*Cleome gynandra* L.) from different agro ecological zones in Malawi. Afr J Food Sci. 2017;11(1):24–9. doi: 10.5897/ajfs2016.1478

[67] Moenga MN, Mogwasi R, Okemwa EK, Olale KO. Determination of Essential Minerals in the Indigenous Vegetables *Solanum nigrum* (Stout Shade) and *Gynandropsis gynandra* (Spider Plant) from Two Agroecological Zones in Kisii County, Kenya. Biol Trace Elem Res. 2025;203(4):2365–81. doi: 10.1007/s12011-024-04312-3 39023736

[68] Teddy FM, John MM, Akwilina WM. Vitamin C, iron and zinc levels of selected African green leafy vegetables at different stages of maturity. Afr J Biotechnol. 2018;17(17):567–73. doi: 10.5897/ajb2017.16346

[69] Grace M, Owuor OP, Ongeri DMK. Assessment of Levels of Nutrients in Selected ALVs at Different Harvesting Stages and Locations of Production in Western Kenya. IJBcRR. 2019;:1–16. doi: 10.9734/ijbcrr/2019/v25i330076

[70] Priyadarshan PM. Pant Breeding: Classical to Modern. Singapore: Springer Nature. 2019.

[71] Singh BK, Sharma SR, Singh B. Genetic combining ability for concentration of mineral elements in cabbage head (*Brassica oleracea* var. *capitata* L.). Euphytica. 2011;184(2):265–73. doi: 10.1007/s10681-011-0598-4

[72] Visscher PM, Hill WG, Wray NR. Heritability in the genomics era--concepts and misconceptions. Nat Rev Genet. 2008;9(4):255–66. doi: 10.1038/nrg2322 18319743

[73] Shukla S, Bhargava A, Chatterjee A, Srivastava J, Singh N, Singh SP. Mineral profile and variability in vegetable amaranth (*Amaranthus tricolor*). Plant Foods Hum Nutr. 2006;61(1):23–8. doi: 10.1007/s11130-006-0004-x 16736385

[74] Karmakar P, Munshi AD, Behera TK, Kumar R, Sureja AK, Kaur C, et al. Quantification and Inheritance of Antioxidant Properties and Mineral Content in Ridge Gourd (*Luffa acutangula*). Agric Res. 2013;2(3):222–8. doi: 10.1007/s40003-013-0070-x

[75] Zhang L, MacQueen A, Bonnette J, Fritschi FB, Lowry DB, Juenger TE. QTL × environment interactions underlie ionome divergence in switchgrass. G3 (Bethesda). 2021;11(7):jkab144. doi: 10.1093/g3journal/jkab144 33914881 PMC8495926

[76] Tan Y, Zhou J, Wang J, Sun L. The Genetic Architecture for Phenotypic Plasticity of the Rice Grain Ionome. Front Plant Sci. 2020;11:12. doi: 10.3389/fpls.2020.00012 32158453 PMC7052182

[77] Asaro A, Ziegler G, Ziyomo C, Hoekenga OA, Dilkes BP, Baxter I. The Interaction of Genotype and Environment Determines Variation in the Maize Kernel Ionome. G3 (Bethesda). 2016;6(12):4175–83. doi: 10.1534/g3.116.034827 27770027 PMC5144985

[78] Phuke RM, Anuradha K, Radhika K, Jabeen F, Anuradha G, Ramesh T, et al. Genetic Variability, Genotype × Environment Interaction, Correlation, and GGE Biplot Analysis for Grain Iron and Zinc Concentration and Other Agronomic Traits in RIL Population of Sorghum (*Sorghum bicolor* L. Moench). Front Plant Sci. 2017;8:712. doi: 10.3389/fpls.2017.00712 28529518 PMC5418227

[79] Yeken MZ. Investigation of genotype × environment interactions for the seed mineral composition in *Phaseolus vulgaris* L. J Food Compost Anal. 2023;124:105657. doi: 10.1016/j.jfca.2023.105657

[80] Sultanbawa F, Sultanbawa Y. Mineral nutrient-rich plants – Do they occur?. Applied Food Research. 2023;3(2):100347. doi: 10.1016/j.afres.2023.100347

[81] Baxter I. Ionomics: studying the social network of mineral nutrients. Curr Opin Plant Biol. 2009;12(3):381–6. doi: 10.1016/j.pbi.2009.05.002 19481970 PMC2701637

[82] Xu Y, Liu X, Fu J, Wang H, Wang J, Huang C, et al. Enhancing Genetic Gain through Genomic Selection: From Livestock to Plants. Plant Commun. 2019;1(1):100005. doi: 10.1016/j.xplc.2019.100005 33404534 PMC7747995

[83] Dey SS, Singh N, Bhatia R, Parkash C, Chandel C. Genetic combining ability and heterosis for important vitamins and antioxidant pigments in cauliflower (*Brassica oleracea* var. *capitata* L.). Euphytica. 2013;195(2):169–81. doi: 10.1007/s10681-013-0981-4

[84] Parkash C, Kumar S, Thakur N, Kumar S, Dey SS, Bhatia R, et al. Genetic analysis of important antioxidant compounds in cabbage (*Brassiaca oleraceavar*.capitataL.). Journal of Crop Improvement. 2017;31(3):418–37. doi: 10.1080/15427528.2017.1310165

[85] Singh S, Dey SS, Bhatia R, Kumar R, Sharma K, Behera TK. Heterosis and combining ability in cytoplasmic male sterile and doubled haploid based *Brassica oleracea* progenies and prediction of heterosis using microsatellites. PLoS One. 2019;14(8):e0210772. doi: 10.1371/journal.pone.0210772 31425498 PMC6699688

[86] Pavan MP, Gangaprasad S, Dushyanthakumar BM, Adivappar N, Shashikumara P. Heterosis and combining ability studies by line × tester analysis for fruit biochemical, morpho-physiological, and yield traits governing shelf life in tomato (*Solanum lycopersicum L*.). Euphytica. 2022;218(7). doi: 10.1007/s10681-022-03038-4

[87] Ceyhan E, Harmankaya M, Kahraman A. Combining ability and heterosis for concentration of mineral elements and protein in common bean (*Phaseolus vulgaris* L.). Turk J Agric For. 2014;38:581–90. doi: 10.3906/tar-1307-56

[88] Ceyhan E, Korkmaz A, Ali A, Karadaş S, Harmankaya M, Şimşek D, et al. Exploring genetic diversity: the inheritance of protein and mineral contents in dwarf common beans. Turkish Journal of Agriculture and Forestry. 2025;49(1):24–36. doi: 10.55730/1300-011x.3246

[89] Tamüksek Ş, Ceyhan E. Genetic Analysis of Grain Yield and its Components in Green Bean for Soils with High Lime Content. Braz arch biol technol. 2024;67. doi: 10.1590/1678-4324-2024231090

[90] Tekin NB, Ceyhan E. Diallel Analysis of Dry Bean Varieties for Seed Yield and Important Traits for Calcareous Soils. Braz arch biol technol. 2023;66. doi: 10.1590/1678-4324-2023230170

[91] Kaushik P, Gramazio P, Vilanova S, Raigón MD, Prohens J, Plazas M. Phenolics content, fruit flesh colour and browning in cultivated eggplant, wild relatives and interspecific hybrids and implications for fruit quality breeding. Food Res Int. 2017;102:392–401. doi: 10.1016/j.foodres.2017.09.028 29195964

[92] Fujimoto R, Uezono K, Ishikura S, Osabe K, Peacock WJ, Dennis ES. Recent research on the mechanism of heterosis is important for crop and vegetable breeding systems. Breed Sci. 2018;68(2):145–58. doi: 10.1270/jsbbs.17155 29875598 PMC5982191

[93] Liu J, Li M, Zhang Q, Wei X, Huang X. Exploring the molecular basis of heterosis for plant breeding. J Integr Plant Biol. 2020;62(3):287–98. doi: 10.1111/jipb.12804 30916464

[94] Bar-Zvi D, Lupo O, Levy AA, Barkai N. Hybrid vigor: The best of both parents, or a genomic clash?. Current Opinion in Systems Biology. 2017;6:22–7. doi: 10.1016/j.coisb.2017.08.004

[95] Zhou G, Chen Y, Yao W, Zhang C, Xie W, Hua J, et al. Genetic composition of yield heterosis in an elite rice hybrid. Proc Natl Acad Sci U S A. 2012;109(39):15847–52. doi: 10.1073/pnas.1214141109 23019369 PMC3465387

[96] Sohindji FS, Sogbohossou DEO, Zohoungbogbo HPF, Houdegbe CA, Achigan-Dako EG. Understanding Molecular Mechanisms of Seed Dormancy for Improved Germination in Traditional Leafy Vegetables: An Overview. Agronomy. 2020;10(1):57. doi: 10.3390/agronomy10010057

[97] Alcock TD, Havlickova L, He Z, Bancroft I, White PJ, Broadley MR, et al. Identification of Candidate Genes for Calcium and Magnesium Accumulation in *Brassica napus* L. by Association Genetics. Front Plant Sci. 2017;8:1968. doi: 10.3389/fpls.2017.01968 29187860 PMC5694822

[98] Broadley MR, Hammond JP, King GJ, Astley D, Bowen HC, Meacham MC, et al. Shoot calcium and magnesium concentrations differ between subtaxa, are highly heritable, and associate with potentially pleiotropic loci in *Brassica oleracea*. Plant Physiol. 2008;146(4):1707–20. doi: 10.1104/pp.107.114645 18281414 PMC2287345

[99] Tang R-J, Luan S. Regulation of calcium and magnesium homeostasis in plants: from transporters to signaling network. Curr Opin Plant Biol. 2017;39:97–105. doi: 10.1016/j.pbi.2017.06.009 28709026

[100] Bradshaw HD Jr. Mutations in CAX1 produce phenotypes characteristic of plants tolerant to serpentine soils. New Phytol. 2005;167(1):81–8. doi: 10.1111/j.1469-8137.2005.01408.x 15948832

[101] Lenz H, Dombinov V, Dreistein J, Reinhard MR, Gebert M, Knoop V. Magnesium deficiency phenotypes upon multiple knockout of *Arabidopsis thaliana* MRS2 clade B genes can be ameliorated by concomitantly reduced calcium supply. Plant Cell Physiol. 2013;54(7):1118–31. doi: 10.1093/pcp/pct062 23628997

[102] Thor K. Calcium—nutrient and messenger. Front. Plant Sci. 2019;10:440. 10.3389/fpls.2019.00440 31073302 PMC6495005

[103] Ahmed N, Zhang B, Bozdar B, Chachar S, Rai M, Li J, et al. The power of magnesium: unlocking the potential for increased yield, quality, and stress tolerance of horticultural crops. Front Plant Sci. 2023;14:1285512. doi: 10.3389/fpls.2023.1285512 37941670 PMC10628537

[104] Tang R-J, Zhao F-G, Garcia VJ, Kleist TJ, Yang L, Zhang H-X, et al. Tonoplast CBL-CIPK calcium signaling network regulates magnesium homeostasis in Arabidopsis. Proc Natl Acad Sci U S A. 2015;112(10):3134–9. doi: 10.1073/pnas.1420944112 25646412 PMC4364200

