# Supplementary material for: Combining ability and heterosis analysis for mineral content in the leafy vegetable Gynandropsis gynandra (L.) Briq
Source: PLoS One. 2025 Sep 12;20(9):e0332095. doi: 10.1371/journal.pone.0332095 (PMC12431277; doi:10.1371/journal.pone.0332095)
Supplement: S1 Table — (DOCX) [file pone.0332095.s001.docx]

**S1 Table.** Representation of the North Carolina Design II implemented to generate the 118 F_1_ hybrids used in the present study.

| **Females** | **Males** | | | | | | | | | | | | | |
| --- | --- | --- | --- | --- | --- | --- | --- | --- | --- | --- | --- | --- | --- | --- |
|  | **P13** | **P14** | **P15** | **P16** | **P17** | **P18** | **P19** | **P20** | **P21** | **P22** | **P23** | **P24** | **P25** | **P26** |
| **P01** | X | X |  | X | X | X | X | X | X | X | X |  | X | X |
| **P02** | X | X | X | X | X | X | X | X |  |  | X | X | X | X |
| **P03** | X | X | X | X | X | X | X | X |  | X | X | X | X |  |
| **P04** |  |  | X |  | X | X | X | X | X |  |  | X | X |  |
| **P05** | X | X | X | X |  |  | X |  |  | X | X | X | X | X |
| **P06** | X | X |  | X | X | X | X |  | X | X |  |  |  | X |
| **P07** |  |  | X | X | X | X | X | X | X |  |  |  | X | X |
| **P08** |  | X | X | X | X | X | X |  | X | X | X |  |  |  |
| **P09** |  |  | X | X |  | X | X | X | X |  | X | X |  |  |
| **P10** | X | X |  | X | X | X | X | X |  | X |  |  | X | X |
| **P11** | X | X | X | X |  |  | X | X |  | X |  |  | X | X |
| **P12** | X | X | X | X | X |  | X | X |  | X |  |  | X | X |

**X** : represents a single cross hybrid.
