# Supplementary figures and images for: Combining ability and heterosis analysis for mineral content in the leafy vegetable Gynandropsis gynandra (L.) Briq
Source: PLoS One. 2025 Sep 12;20(9):e0332095. doi: 10.1371/journal.pone.0332095 (PMC12431277; doi:10.1371/journal.pone.0332095)

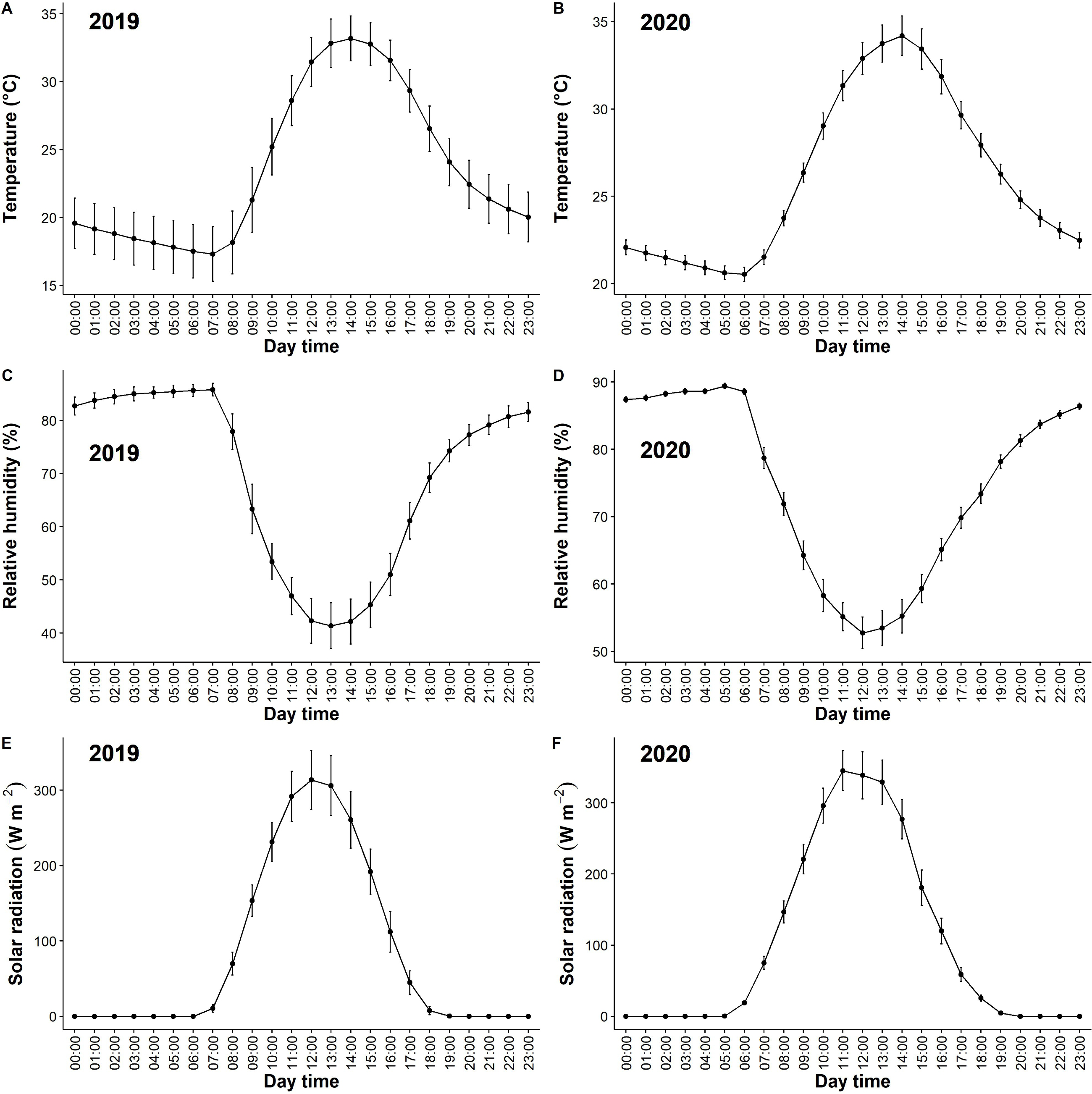

Supplement: S1 Fig — (A) Temperature in 2019. (B) Temperature in 2020. (C) Relative humidity in 2019. (D) Relative humidity in 2020. (E) Solar radiation in 2019. (F) Solar radiation in 2020. (TIF) [file pone.0332095.s003.tif]

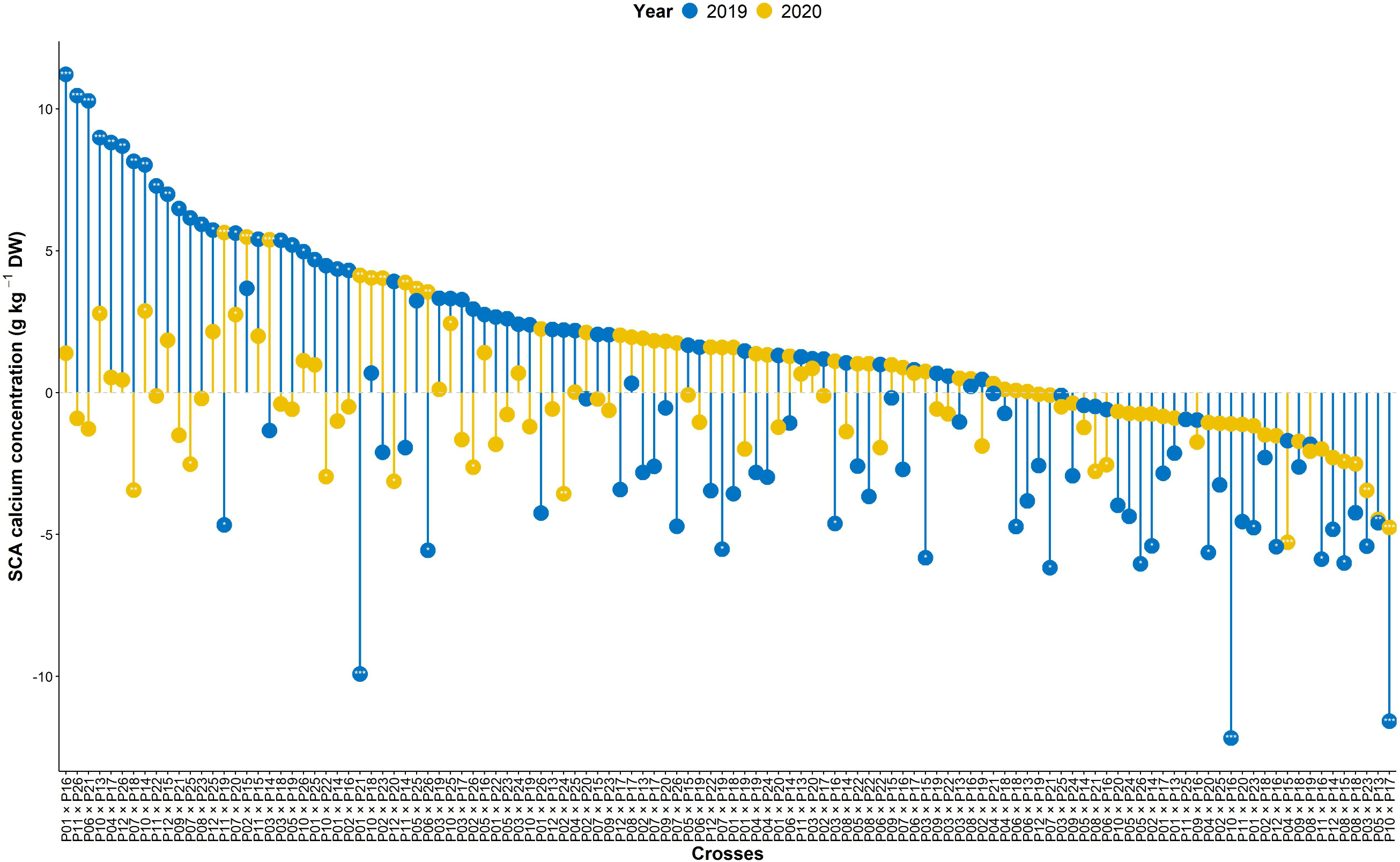

Supplement: S2 Fig — ***, **, * refer to estimate of specific combining ability effect significantly different from zero at p < 0.001, 0.01 and 0.05, respectively. (TIF) [file pone.0332095.s004.tif]

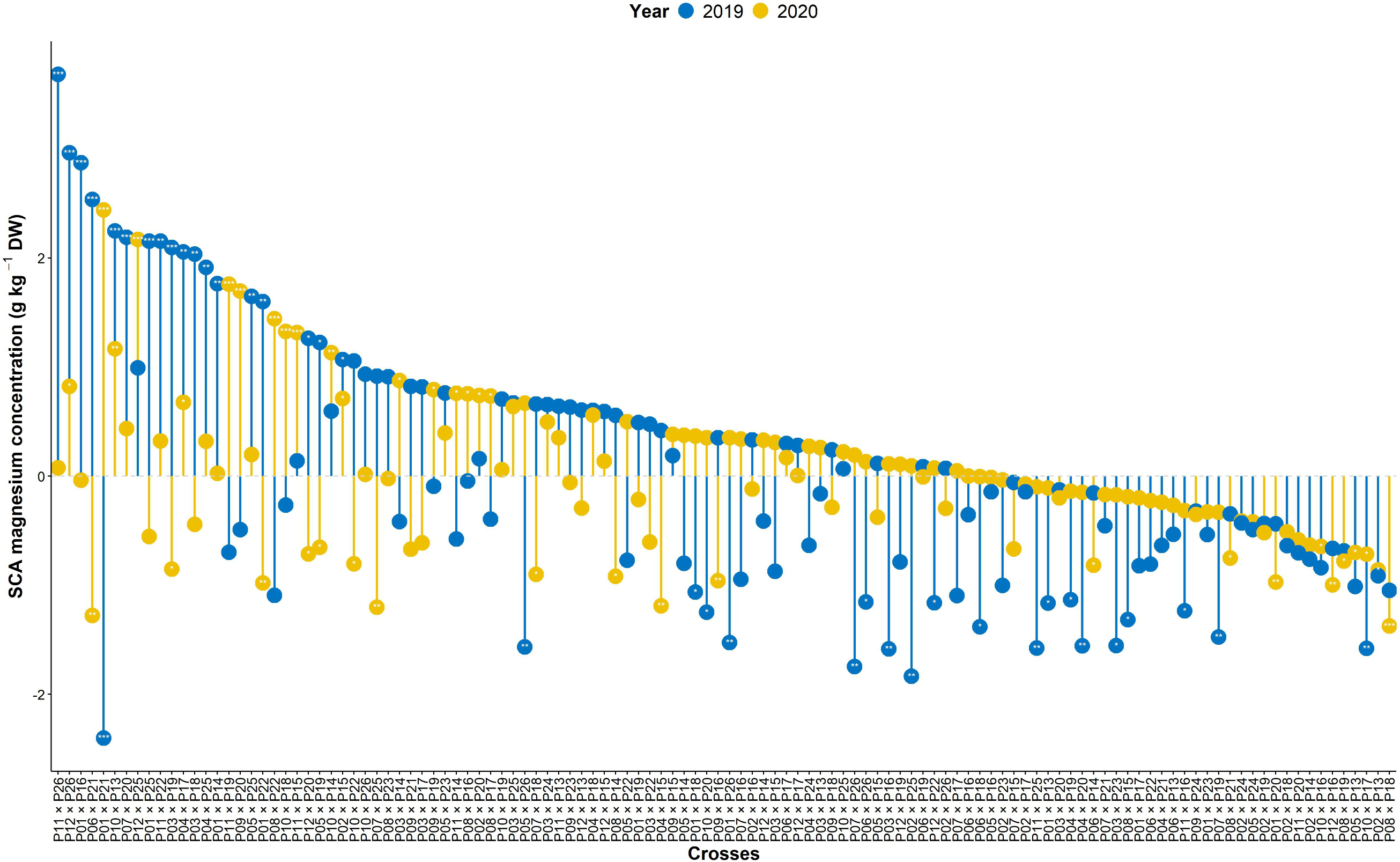

Supplement: S3 Fig — ***, **, * refer to estimate of specific combining ability effect significantly different from zero at p < 0.001, 0.01 and 0.05, respectively. (TIF) [file pone.0332095.s005.tif]

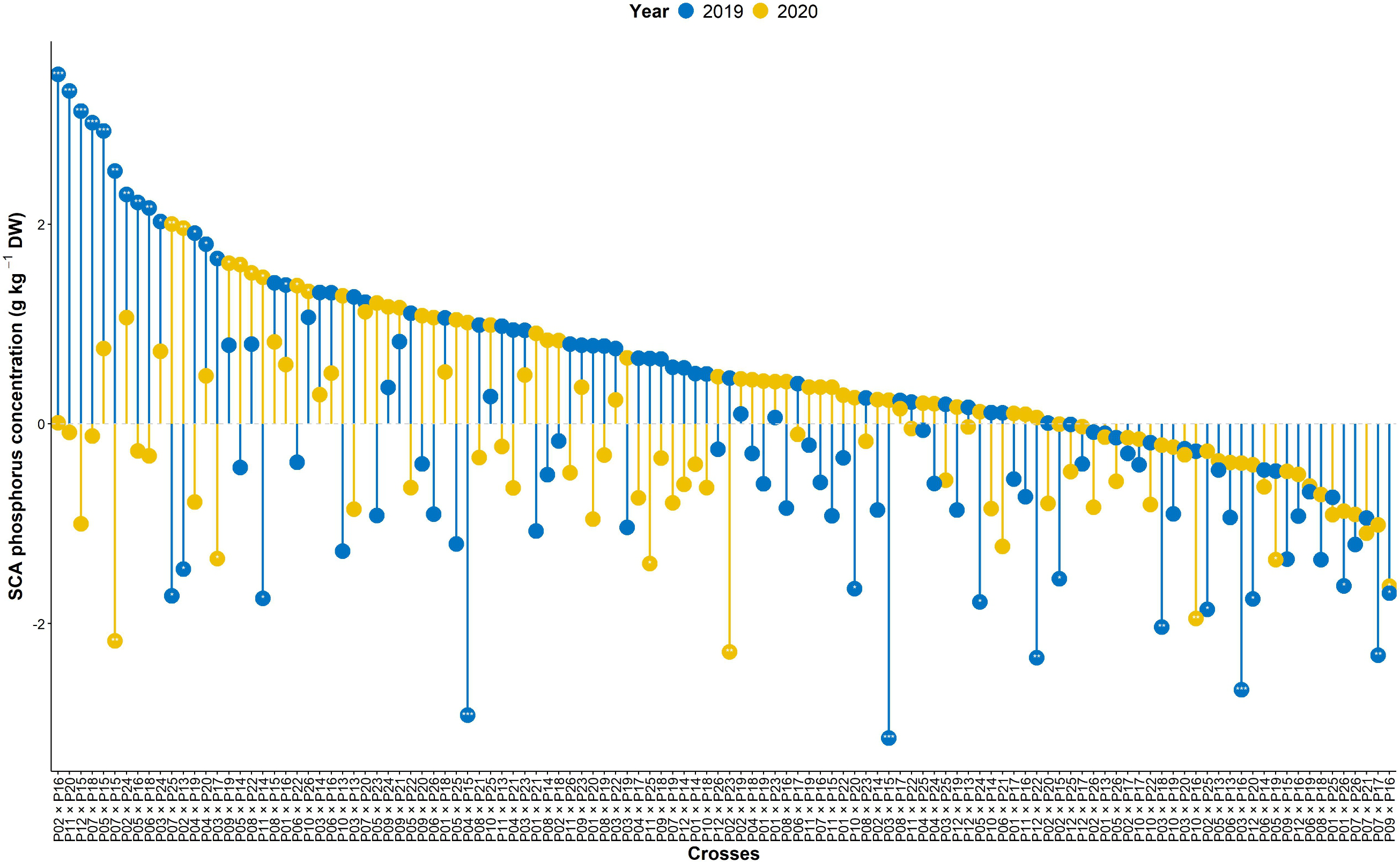

Supplement: S4 Fig — ***, **, * refer to estimate of specific combining ability effect significantly different from zero at p < 0.001, 0.01 and 0.05, respectively. (TIF) [file pone.0332095.s006.tif]

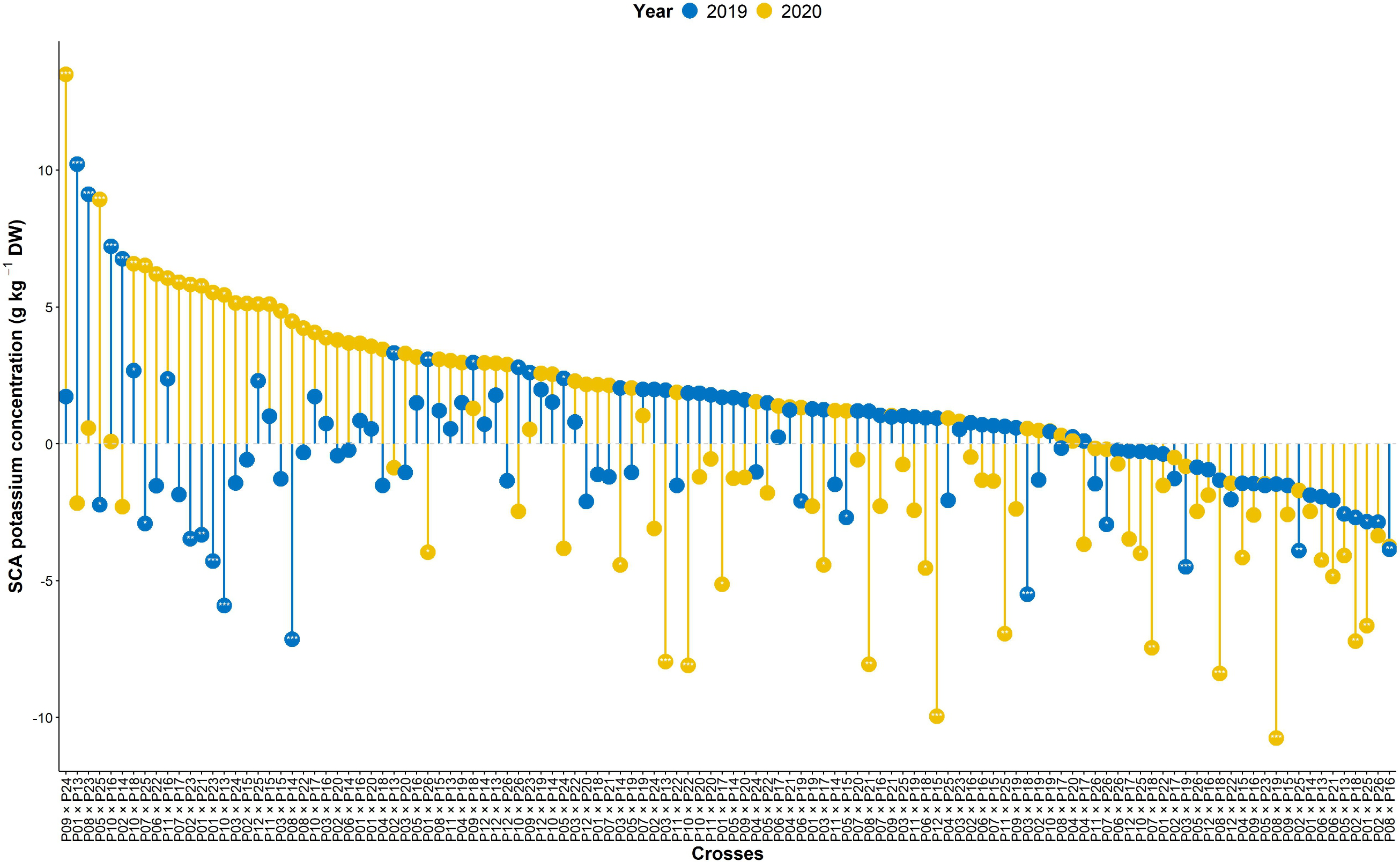

Supplement: S5 Fig — ***, **, * refer to estimate of specific combining ability effect significantly different from zero at p < 0.001, 0.01 and 0.05, respectively. (TIF) [file pone.0332095.s007.tif]

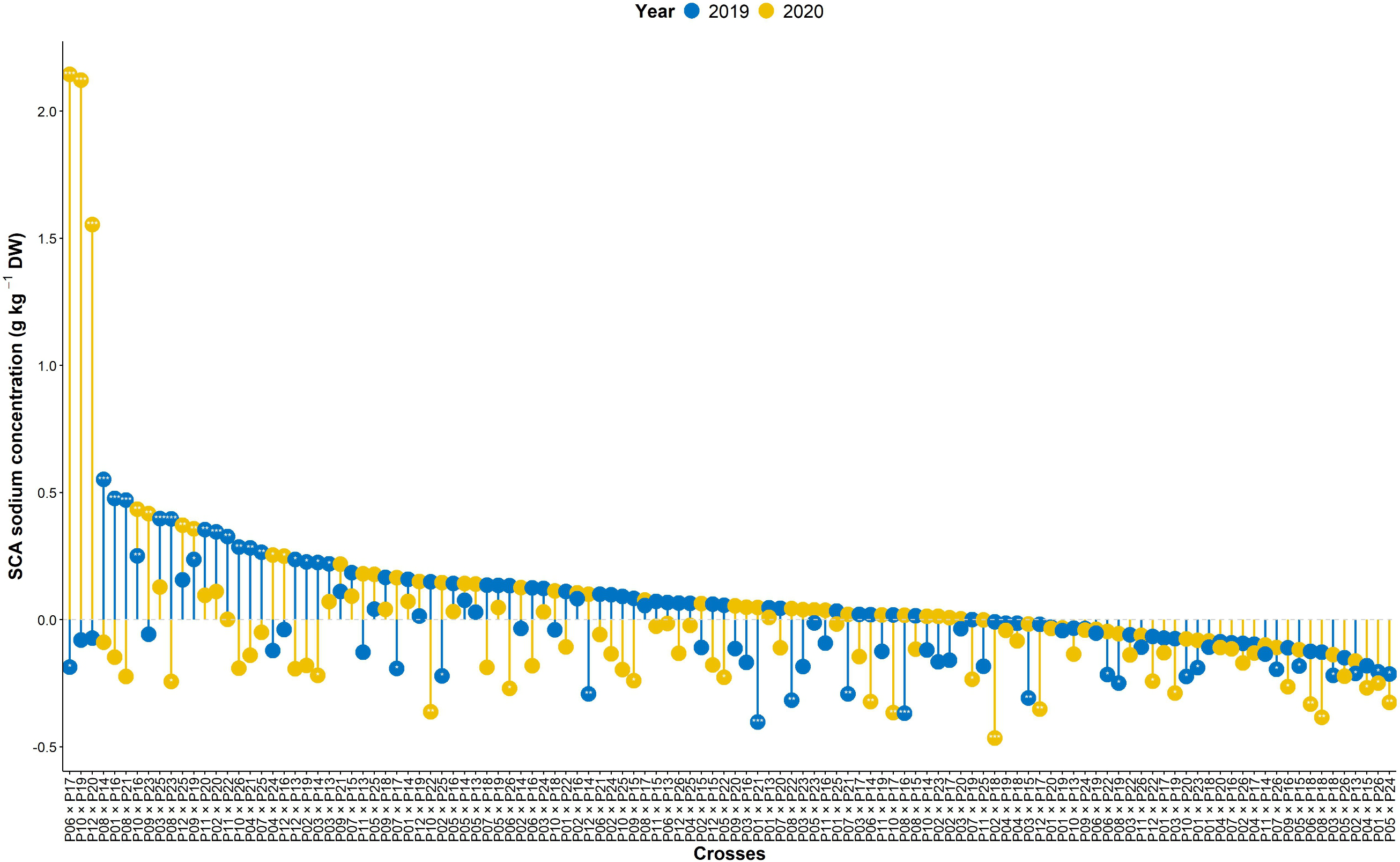

Supplement: S6 Fig — ***, **, * refer to estimate of specific combining ability effect significantly different from zero at p < 0.001, 0.01 and 0.05, respectively. (TIF) [file pone.0332095.s008.tif]

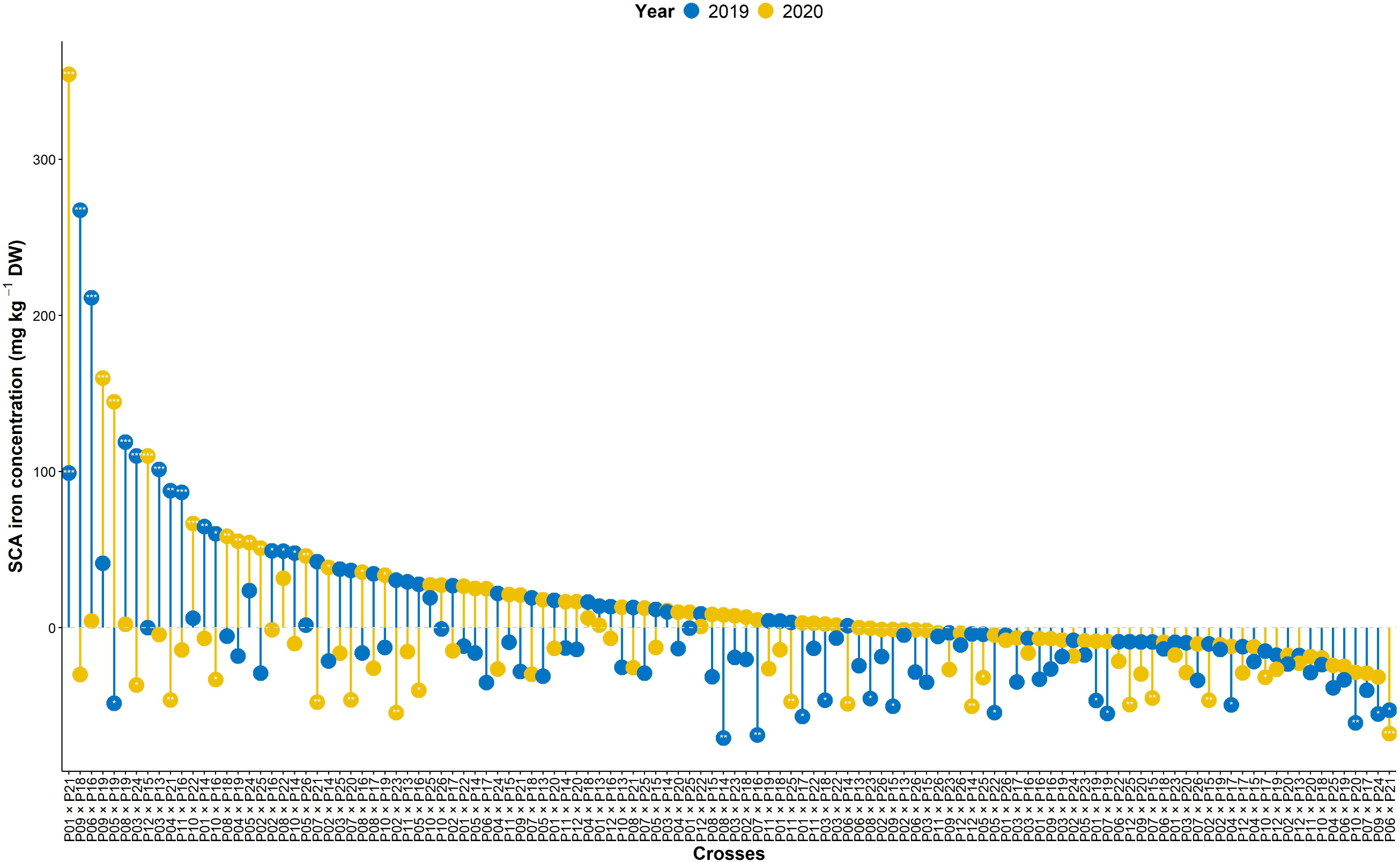

Supplement: S7 Fig — ***, **, * refer to estimate of specific combining ability effect significantly different from zero at p < 0.001, 0.01 and 0.05, respectively. (TIF) [file pone.0332095.s009.tif]

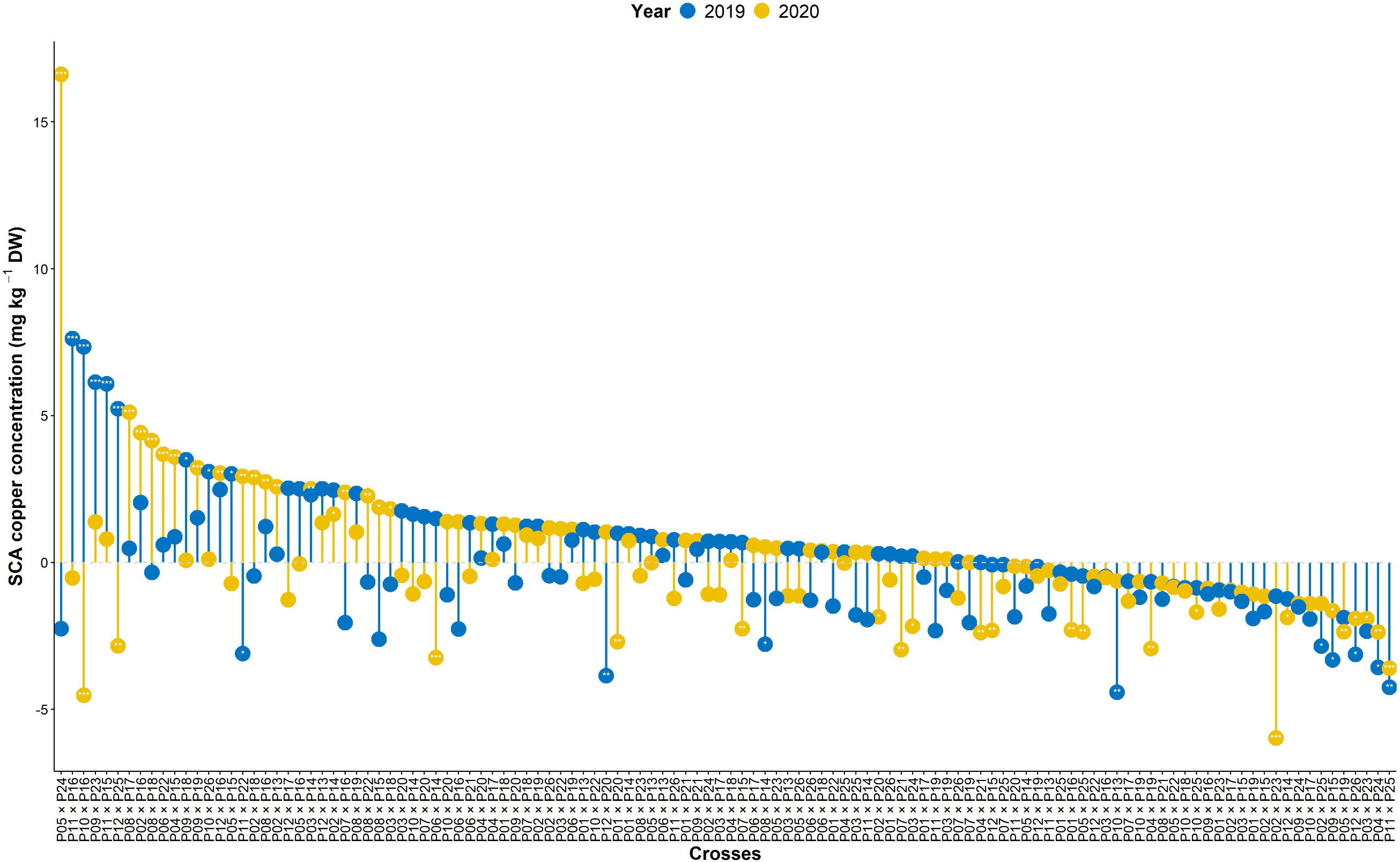

Supplement: S8 Fig — ***, **, * refer to estimate of specific combining ability effect significantly different from zero at p < 0.001, 0.01 and 0.05, respectively. (TIF) [file pone.0332095.s010.tif]

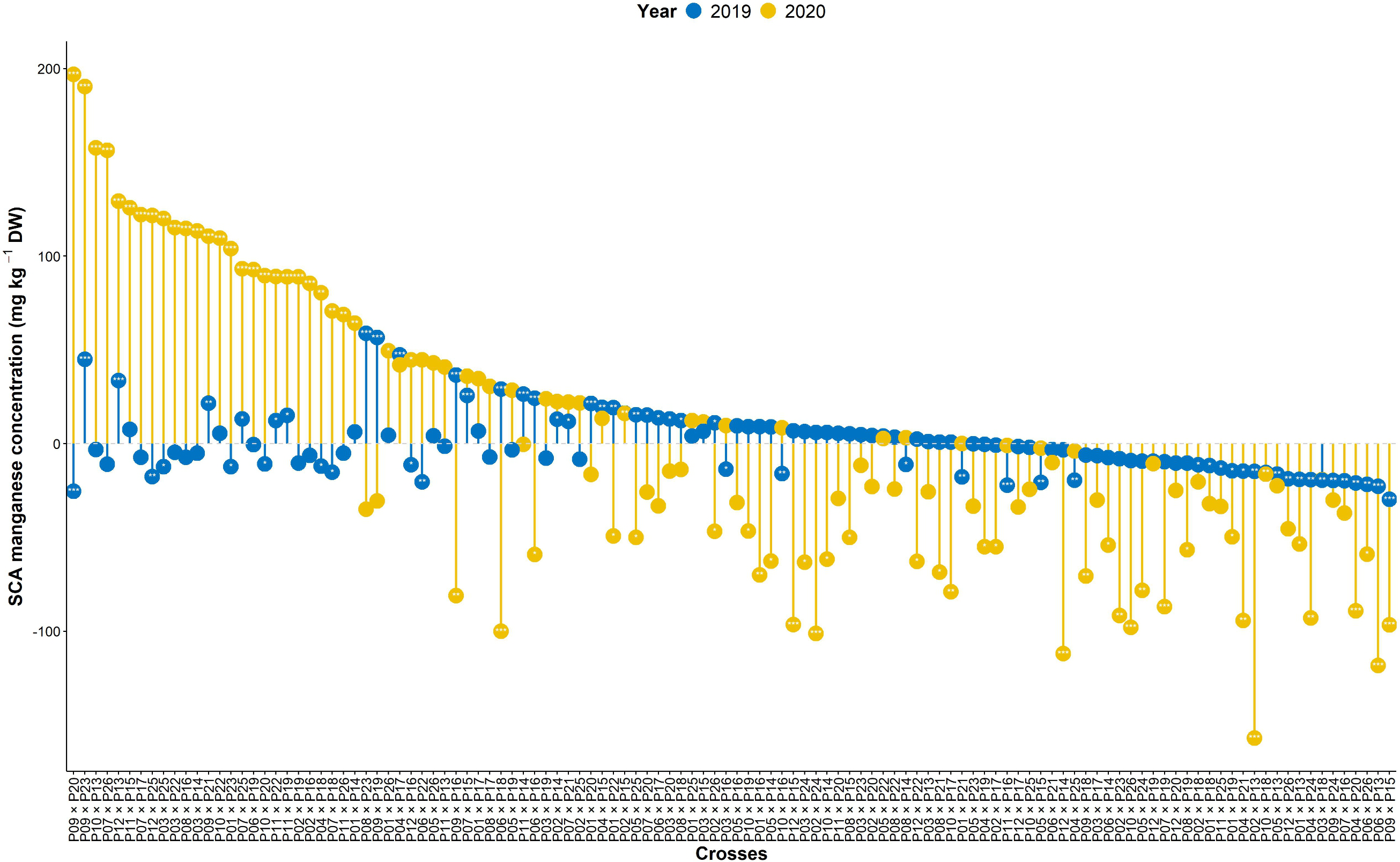

Supplement: S9 Fig — ***, **, * refer to estimate of specific combining ability effect significantly different from zero at p < 0.001, 0.01 and 0.05, respectively. (TIF) [file pone.0332095.s011.tif]

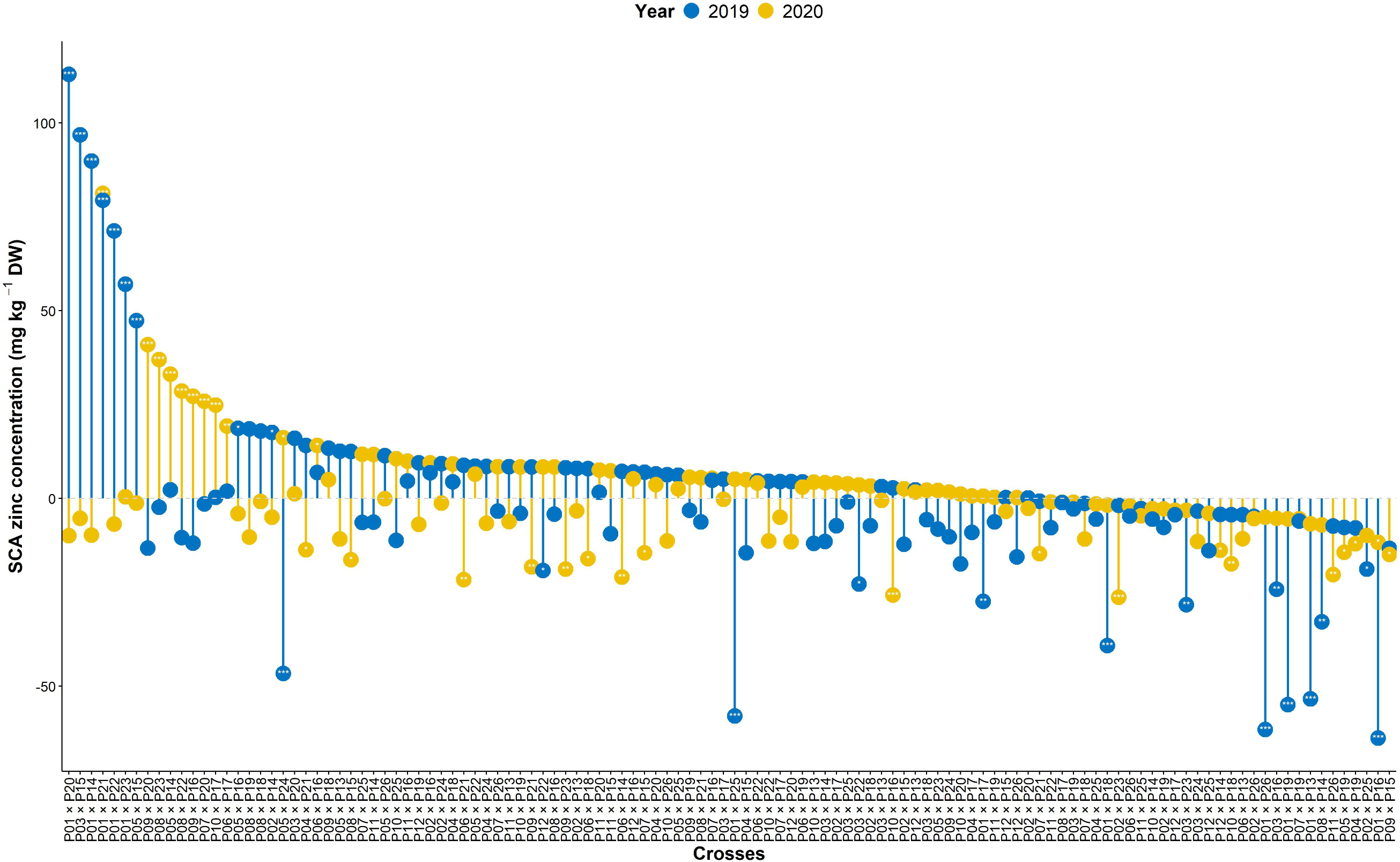

Supplement: S10 Fig — ***, **, * refer to estimate of specific combining ability effect significantly different from zero at p < 0.001, 0.01 and 0.05, respectively. (TIF) [file pone.0332095.s012.tif]

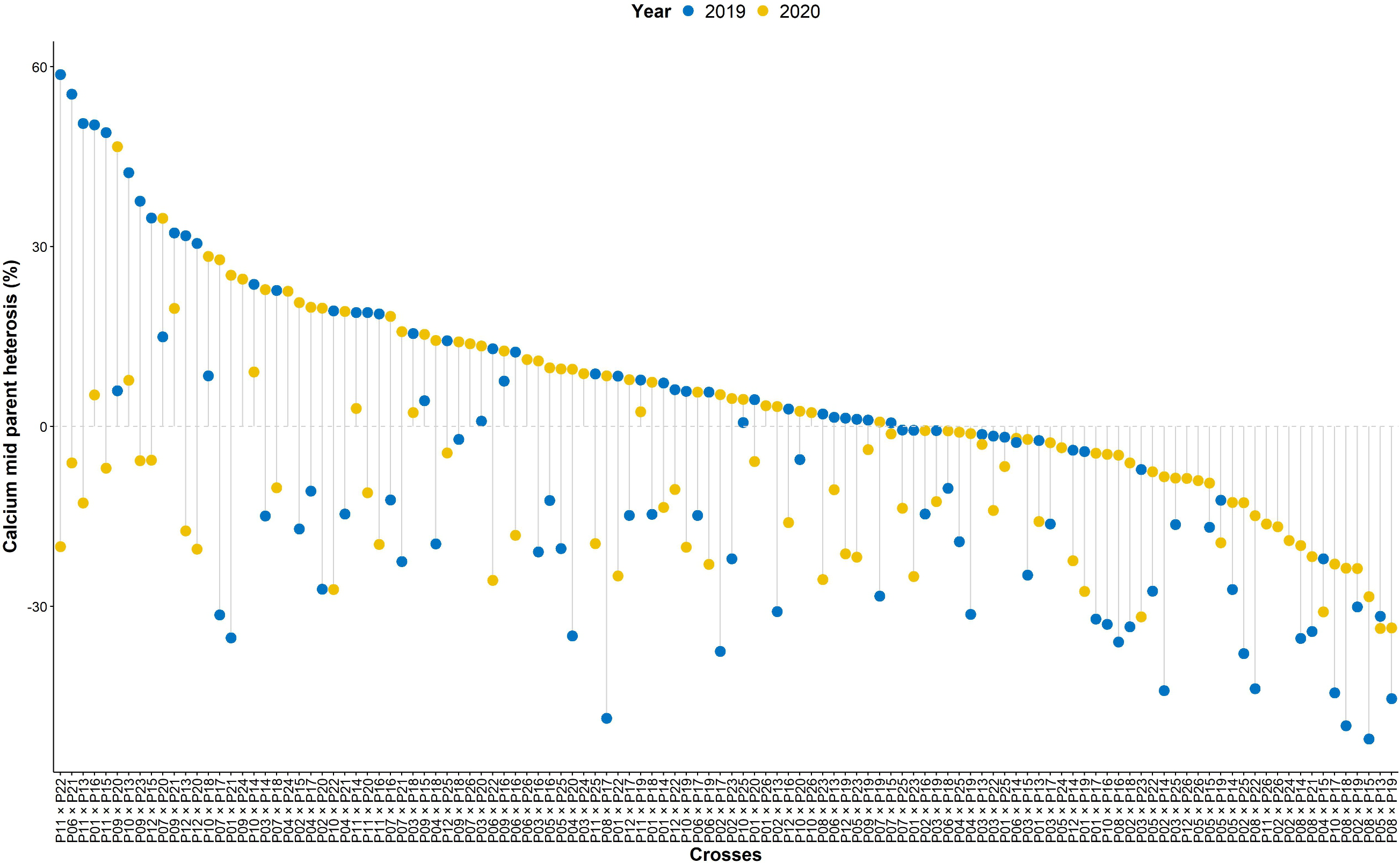

Supplement: S11 Fig — (TIF) [file pone.0332095.s013.tif]

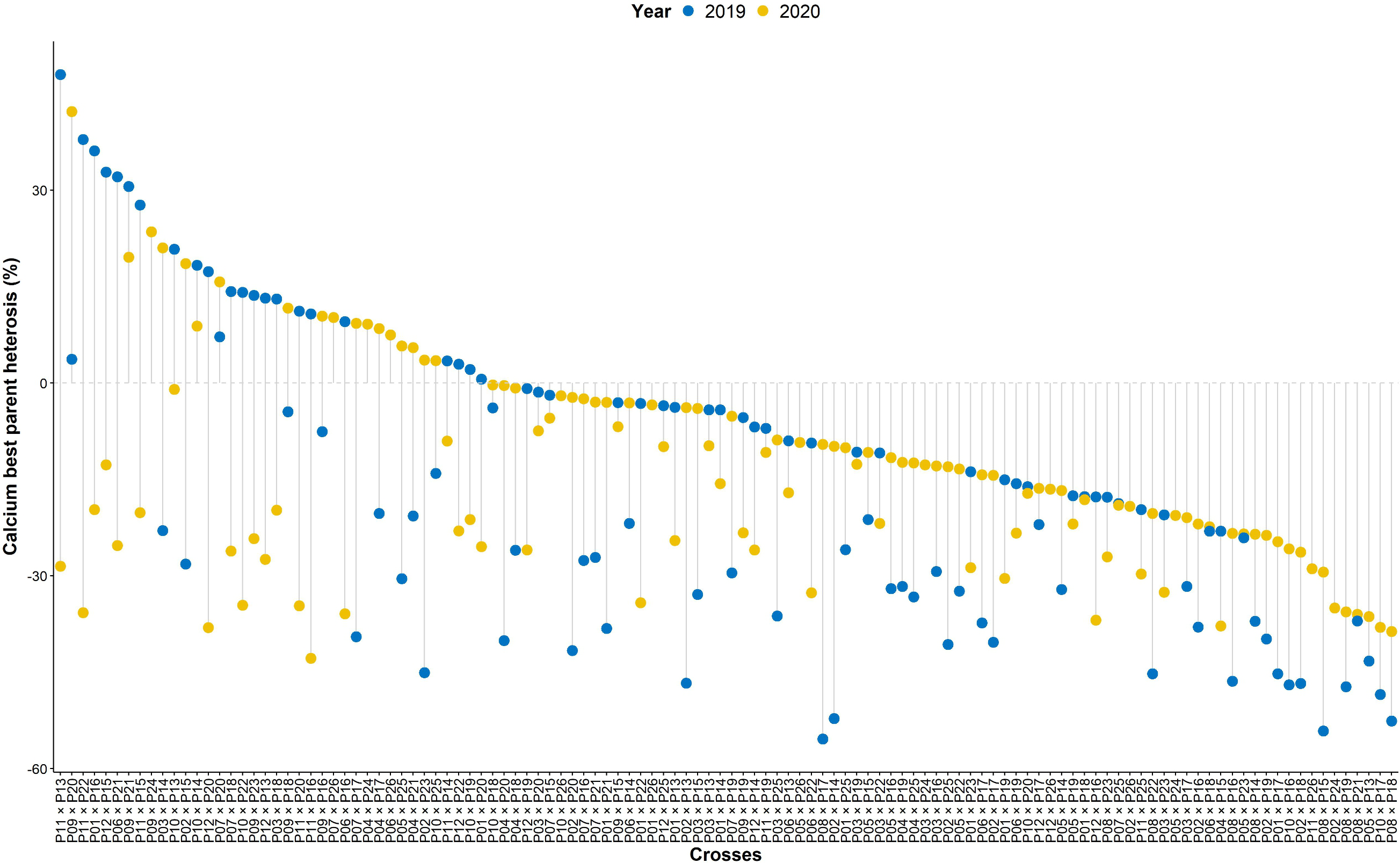

Supplement: S12 Fig — (TIF) [file pone.0332095.s014.tif]

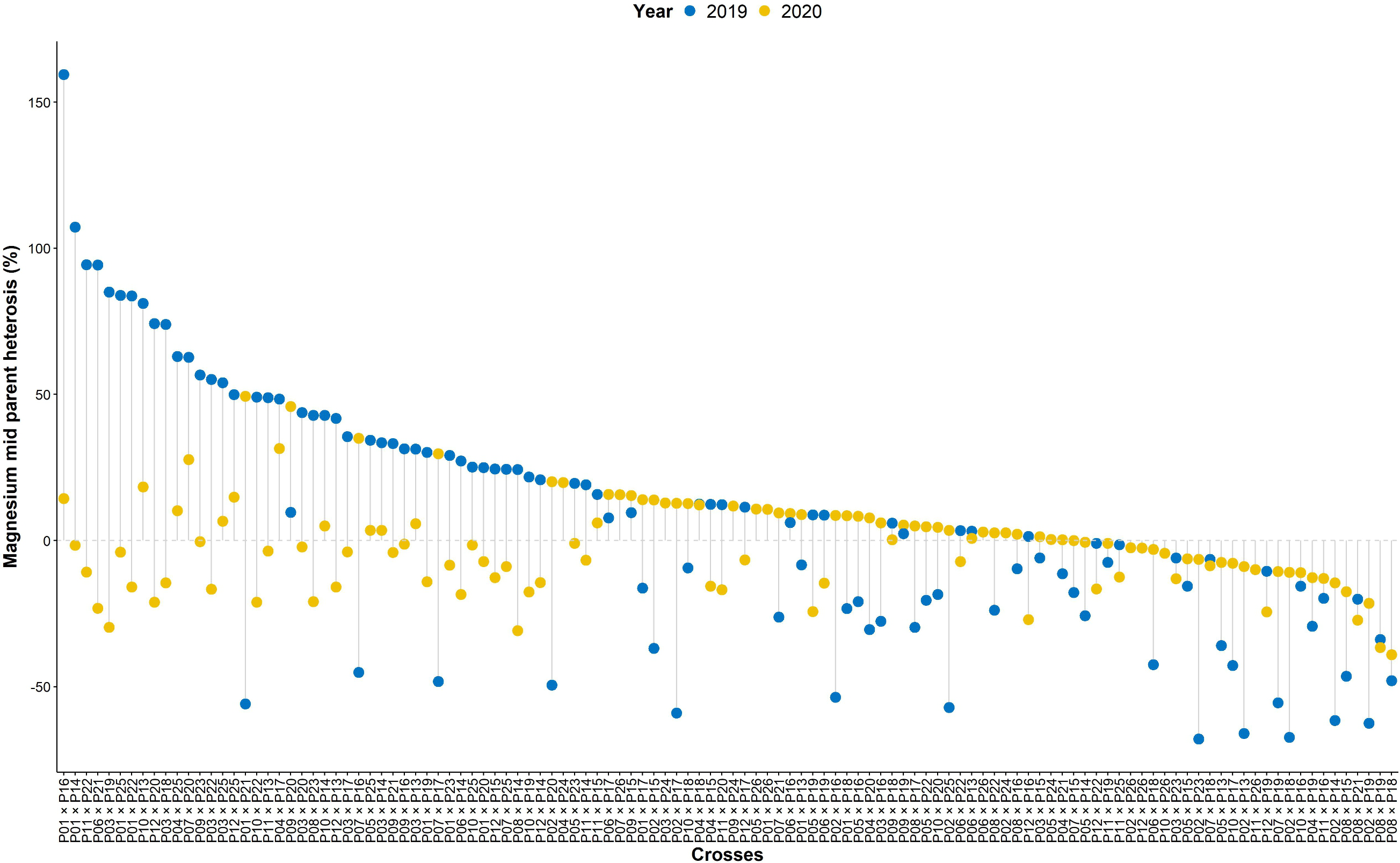

Supplement: S13 Fig — (TIF) [file pone.0332095.s015.tif]

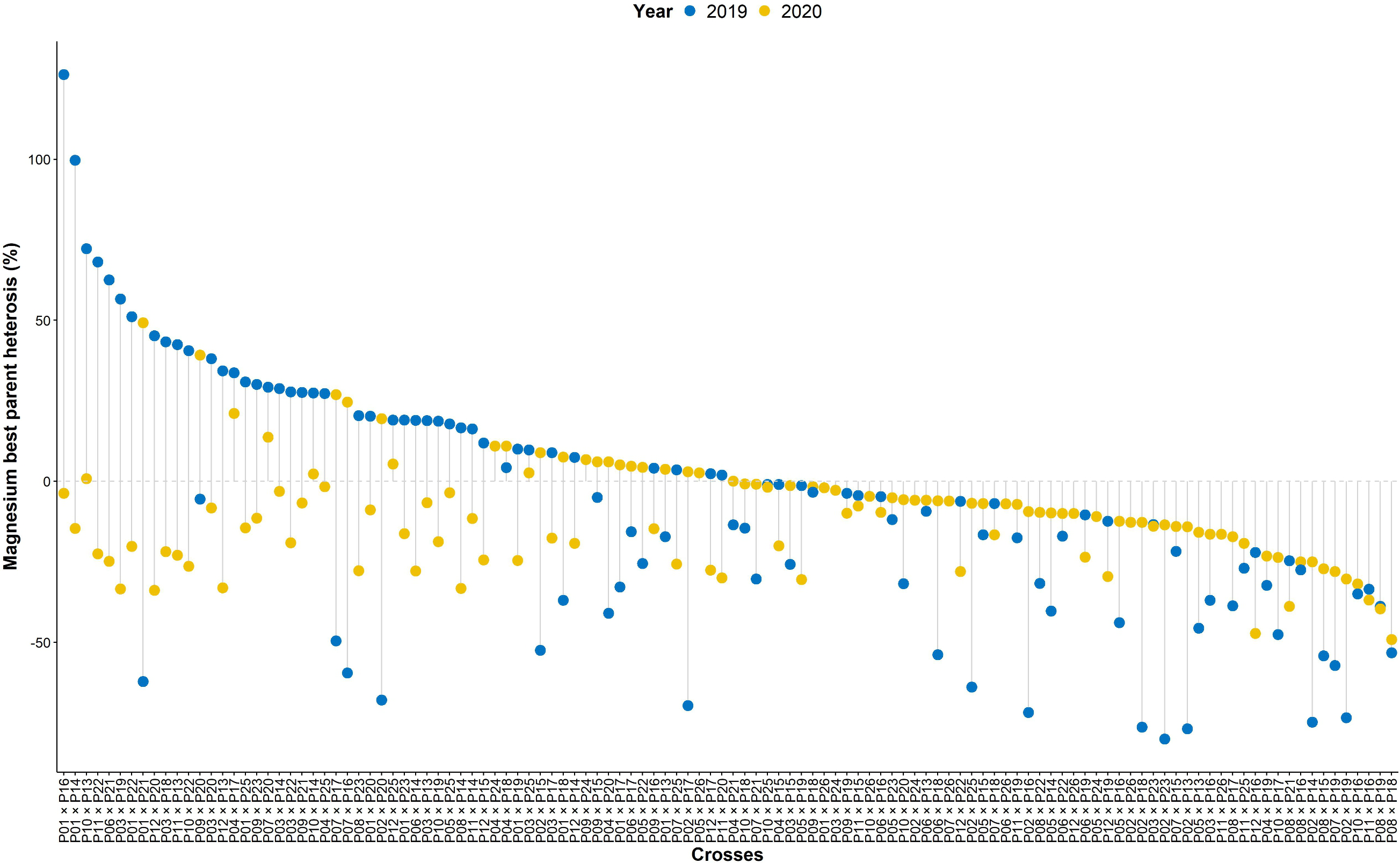

Supplement: S14 Fig — (TIF) [file pone.0332095.s016.tif]

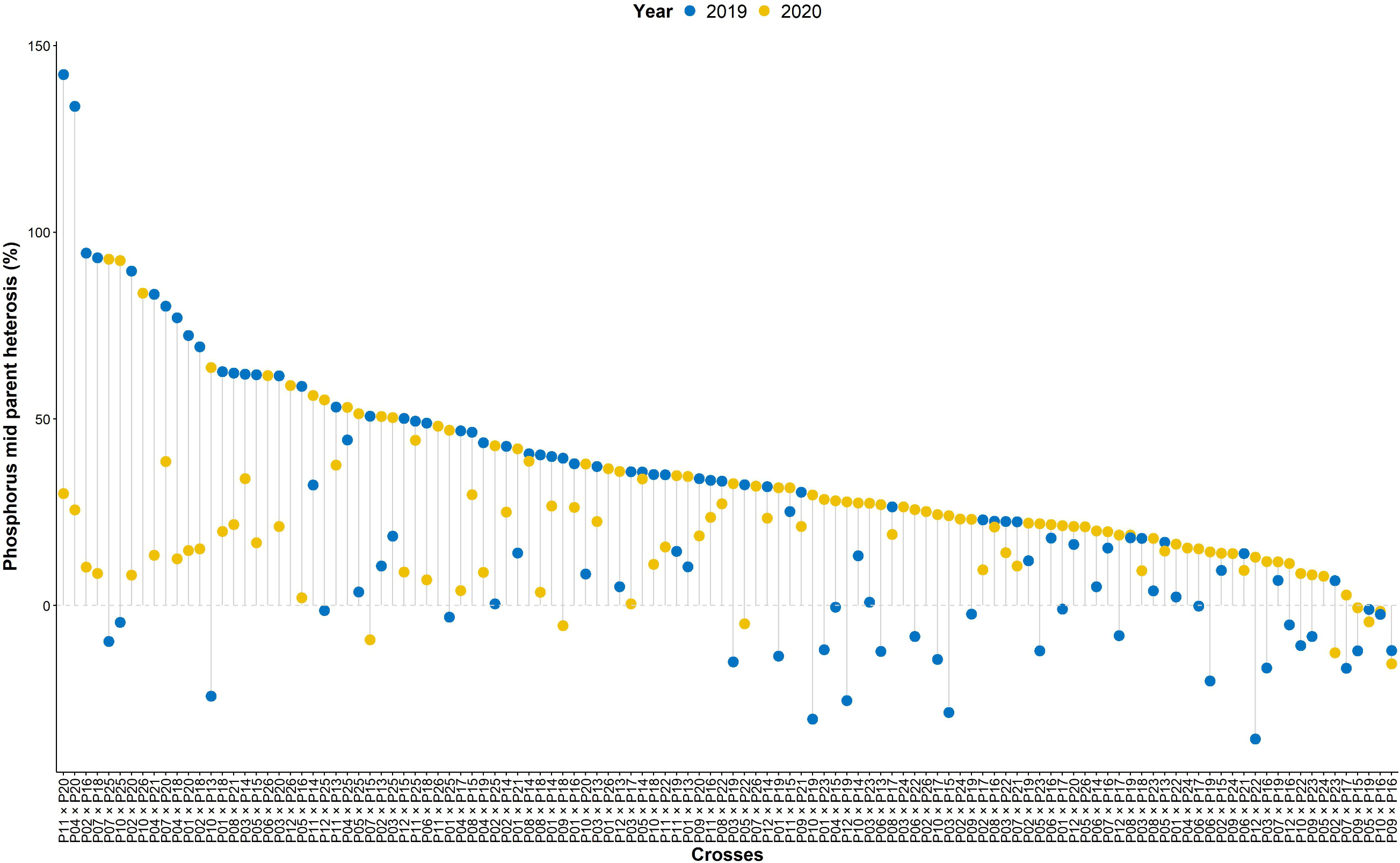

Supplement: S15 Fig — (TIF) [file pone.0332095.s017.tif]

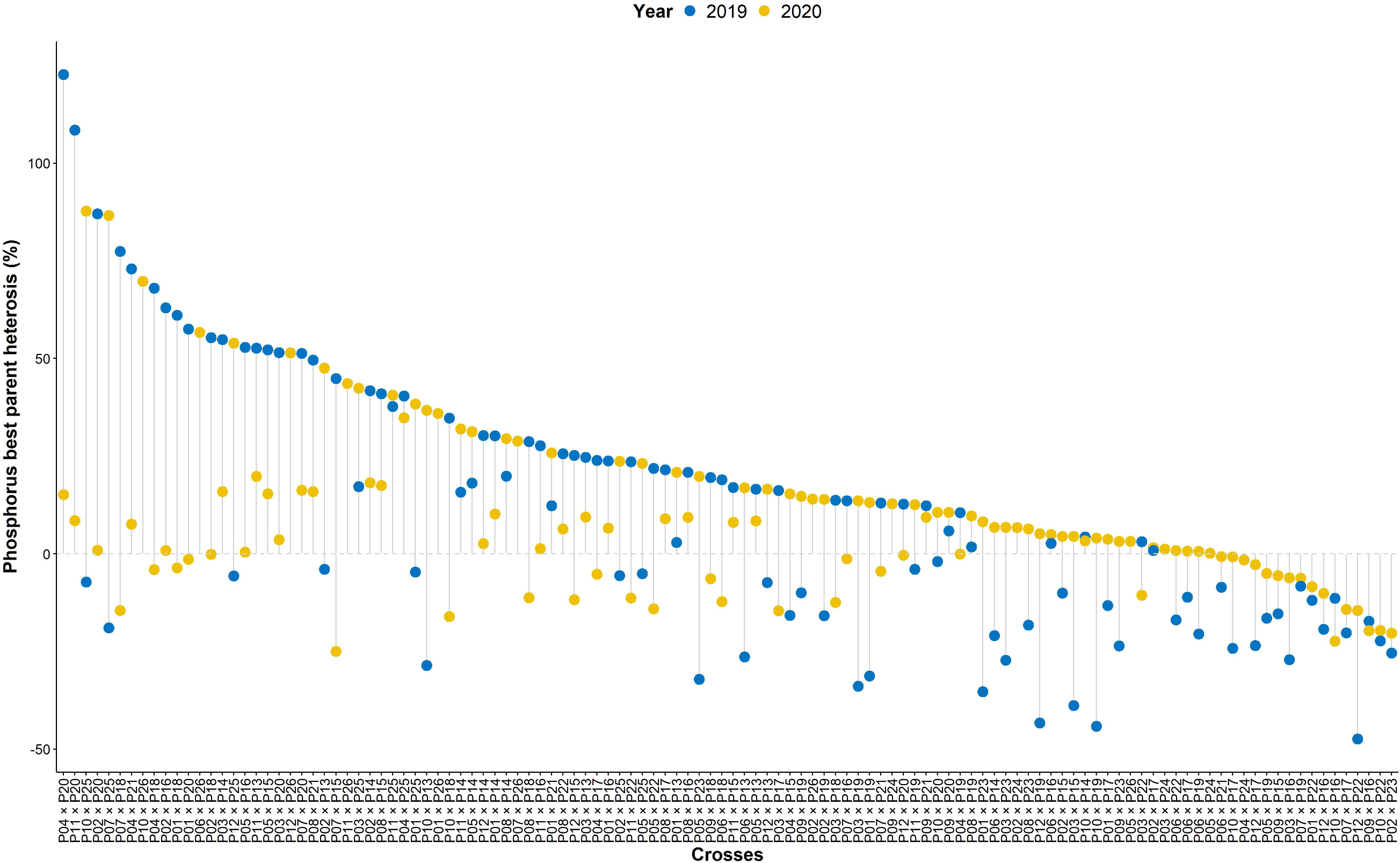

Supplement: S16 Fig — (TIF) [file pone.0332095.s018.tif]

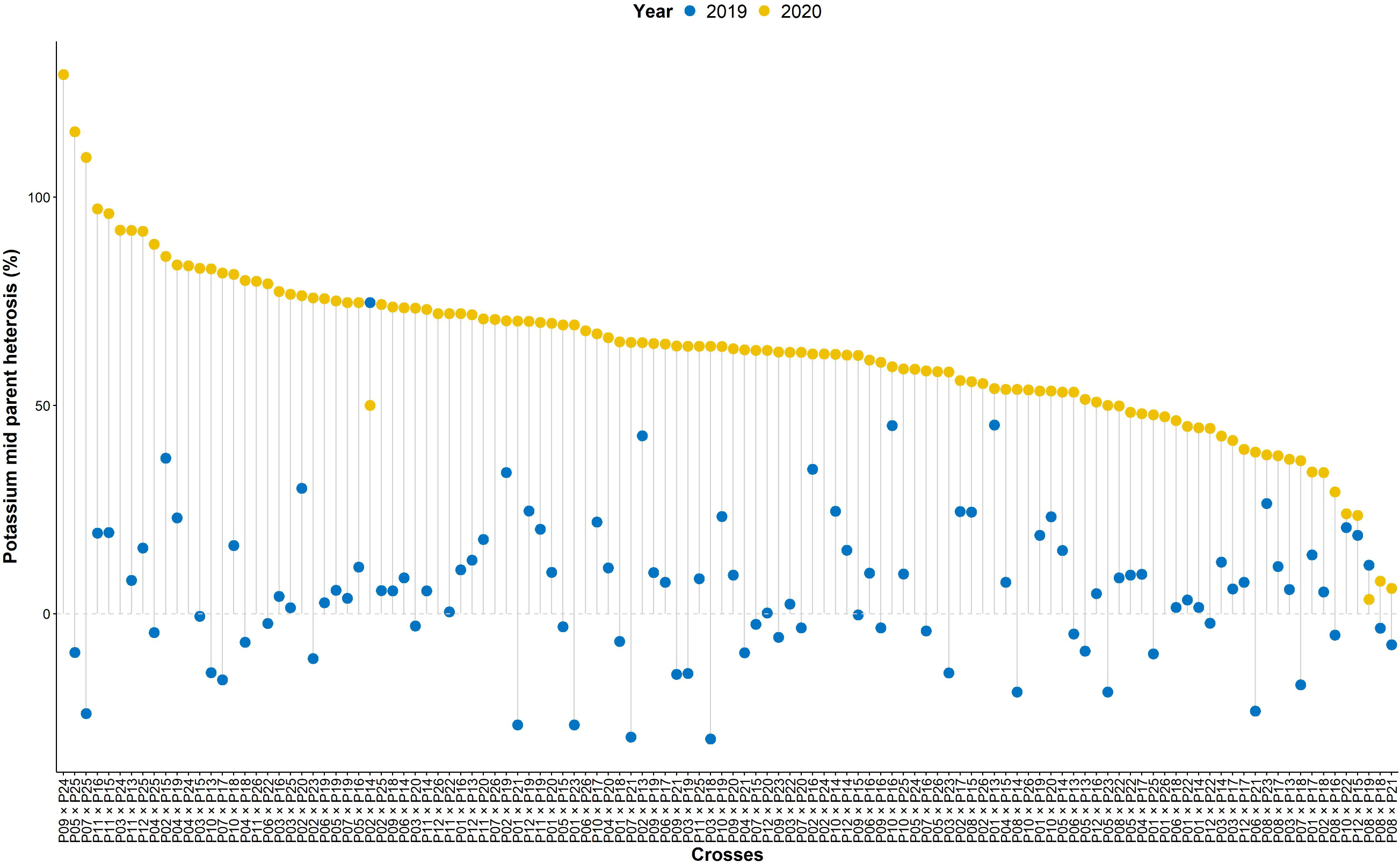

Supplement: S17 Fig — (TIF) [file pone.0332095.s019.tif]

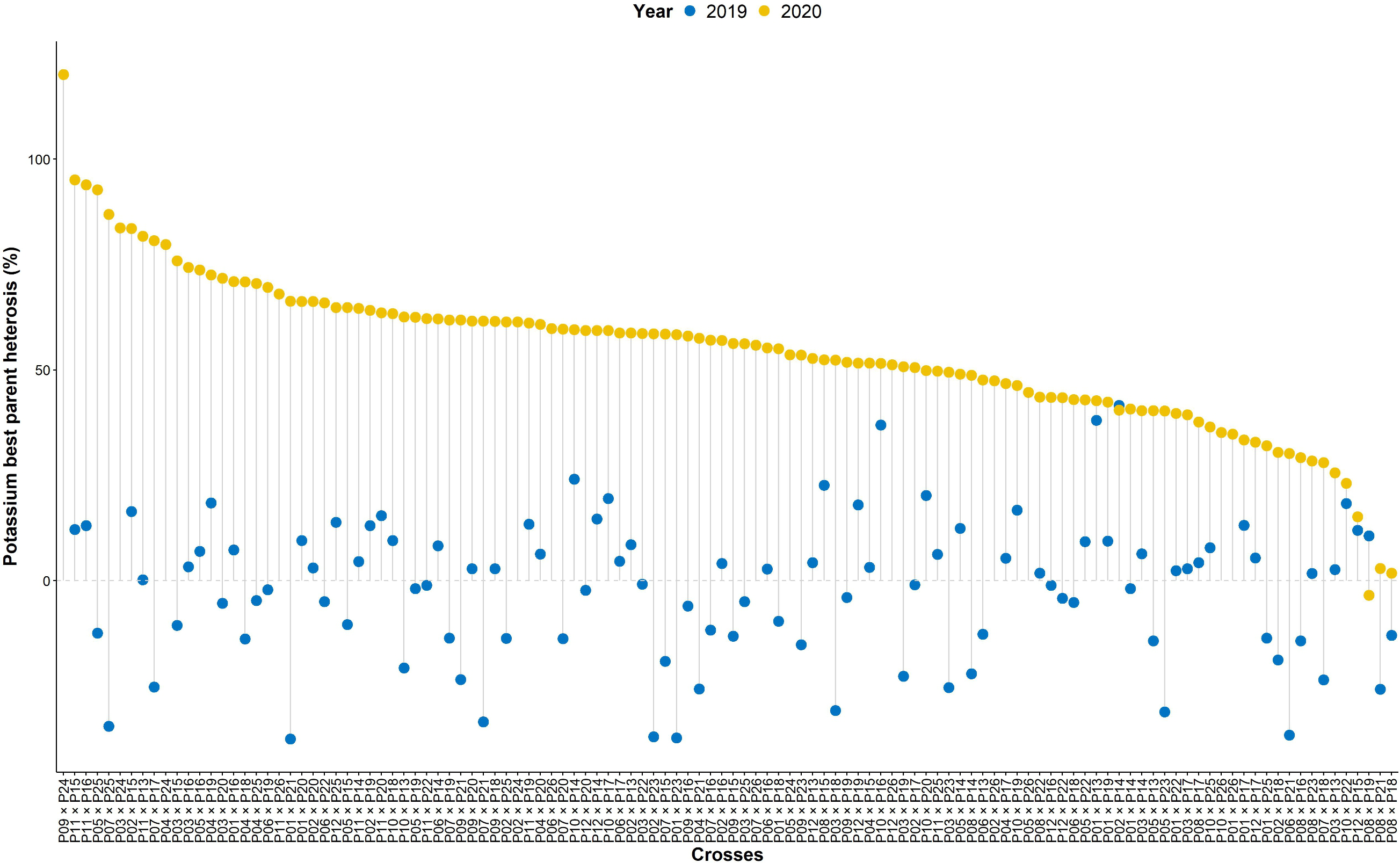

Supplement: S18 Fig — (TIF) [file pone.0332095.s020.tif]

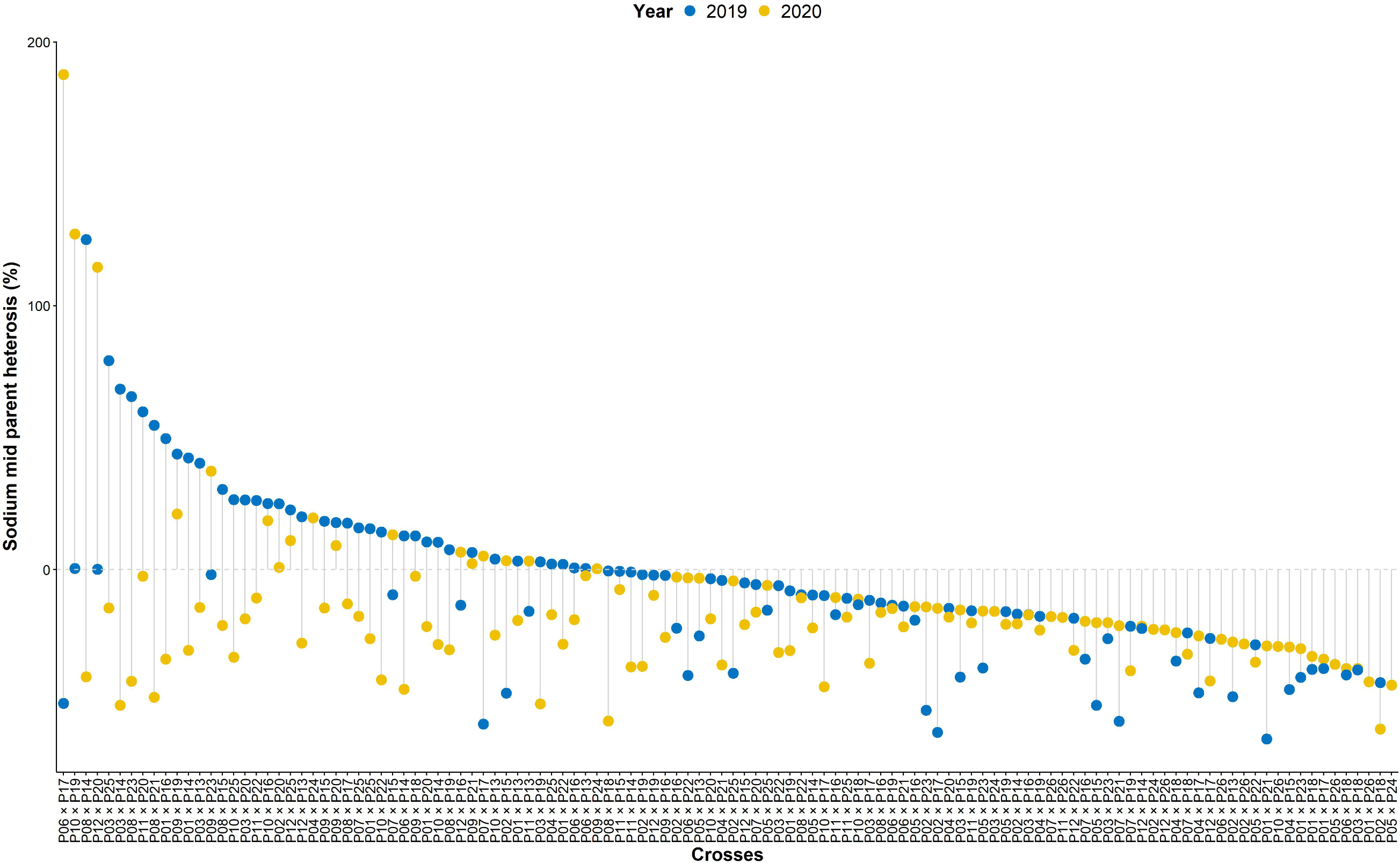

Supplement: S19 Fig — (TIF) [file pone.0332095.s021.tif]

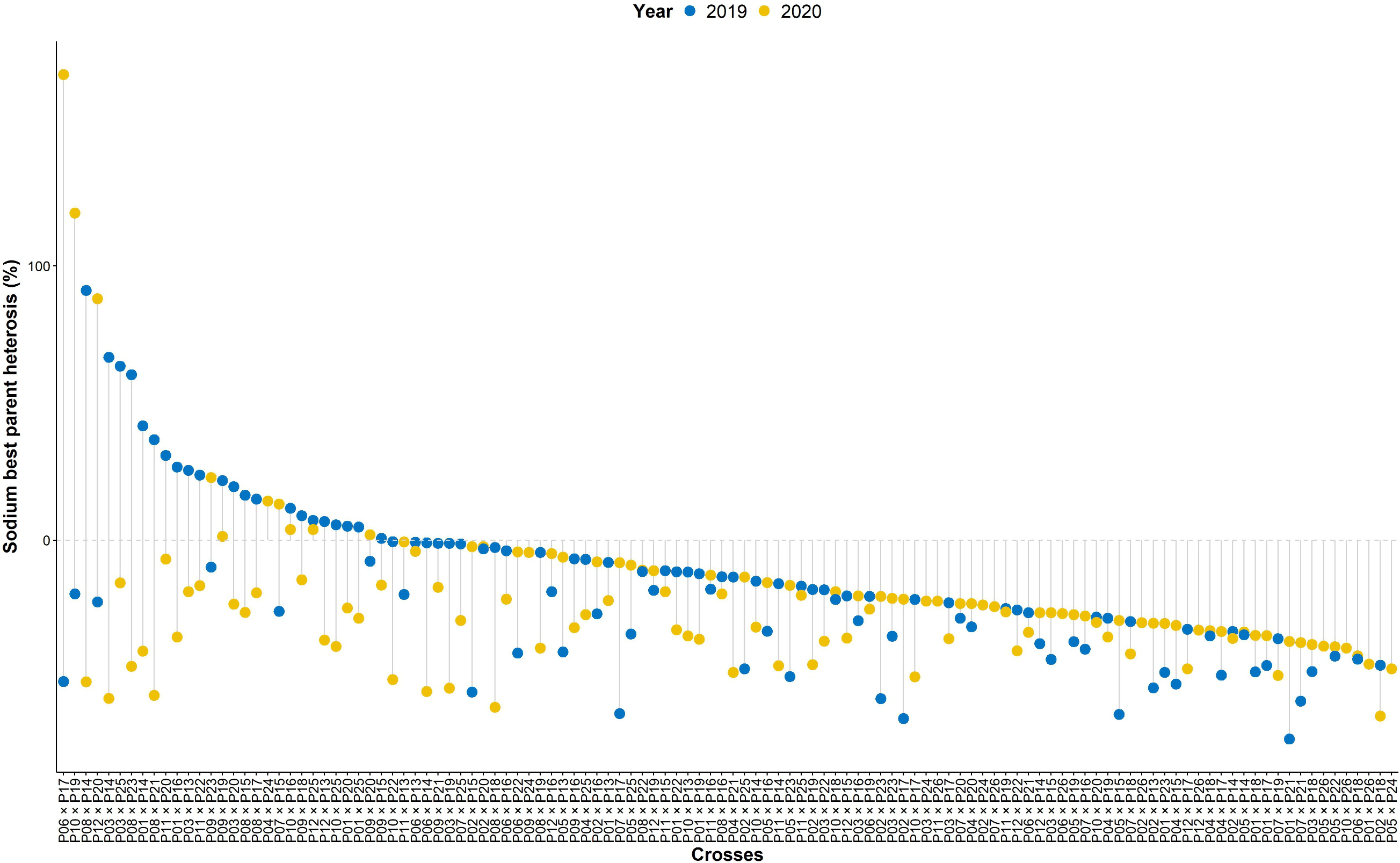

Supplement: S20 Fig — (TIF) [file pone.0332095.s022.tif]

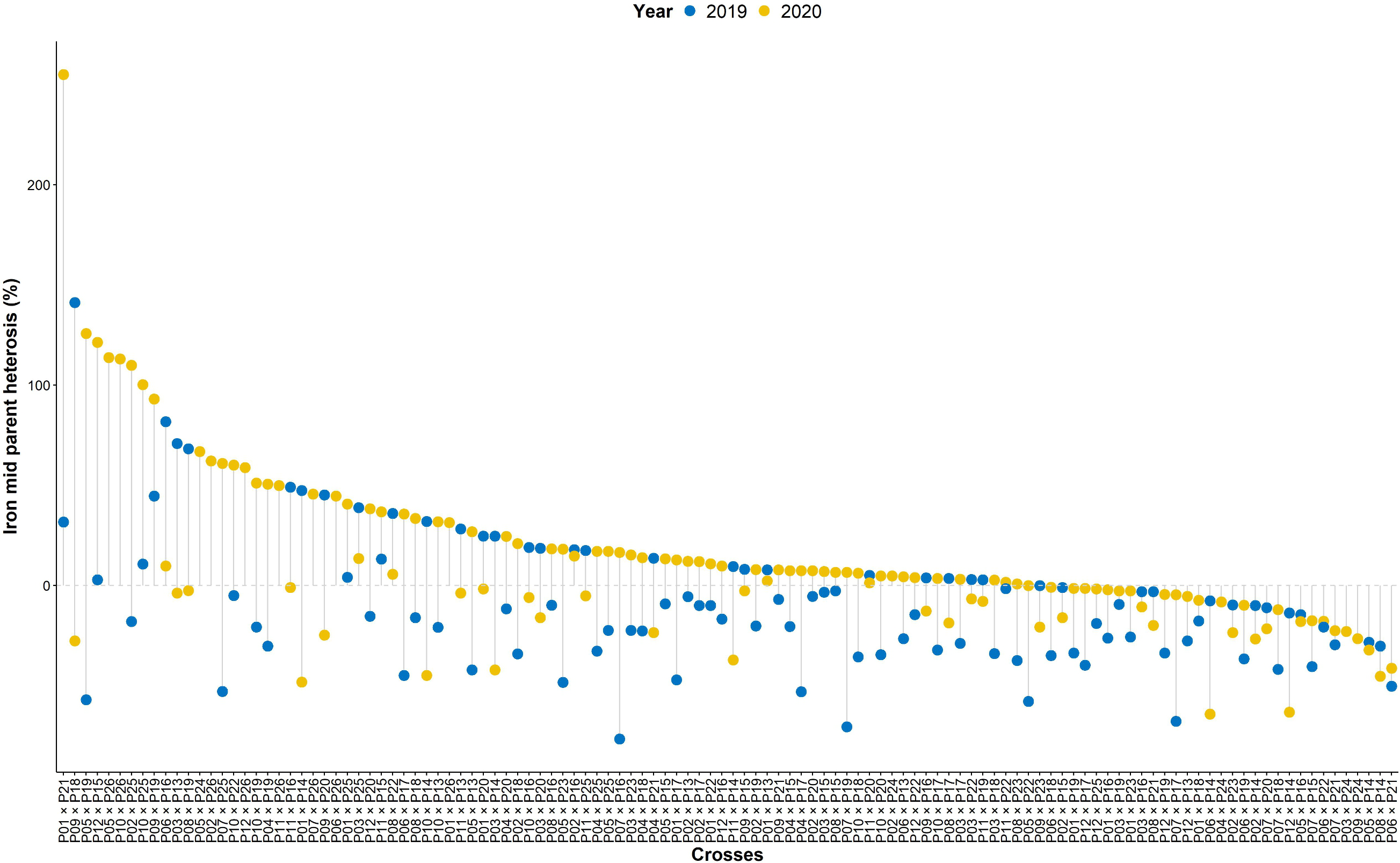

Supplement: S21 Fig — (TIF) [file pone.0332095.s023.tif]

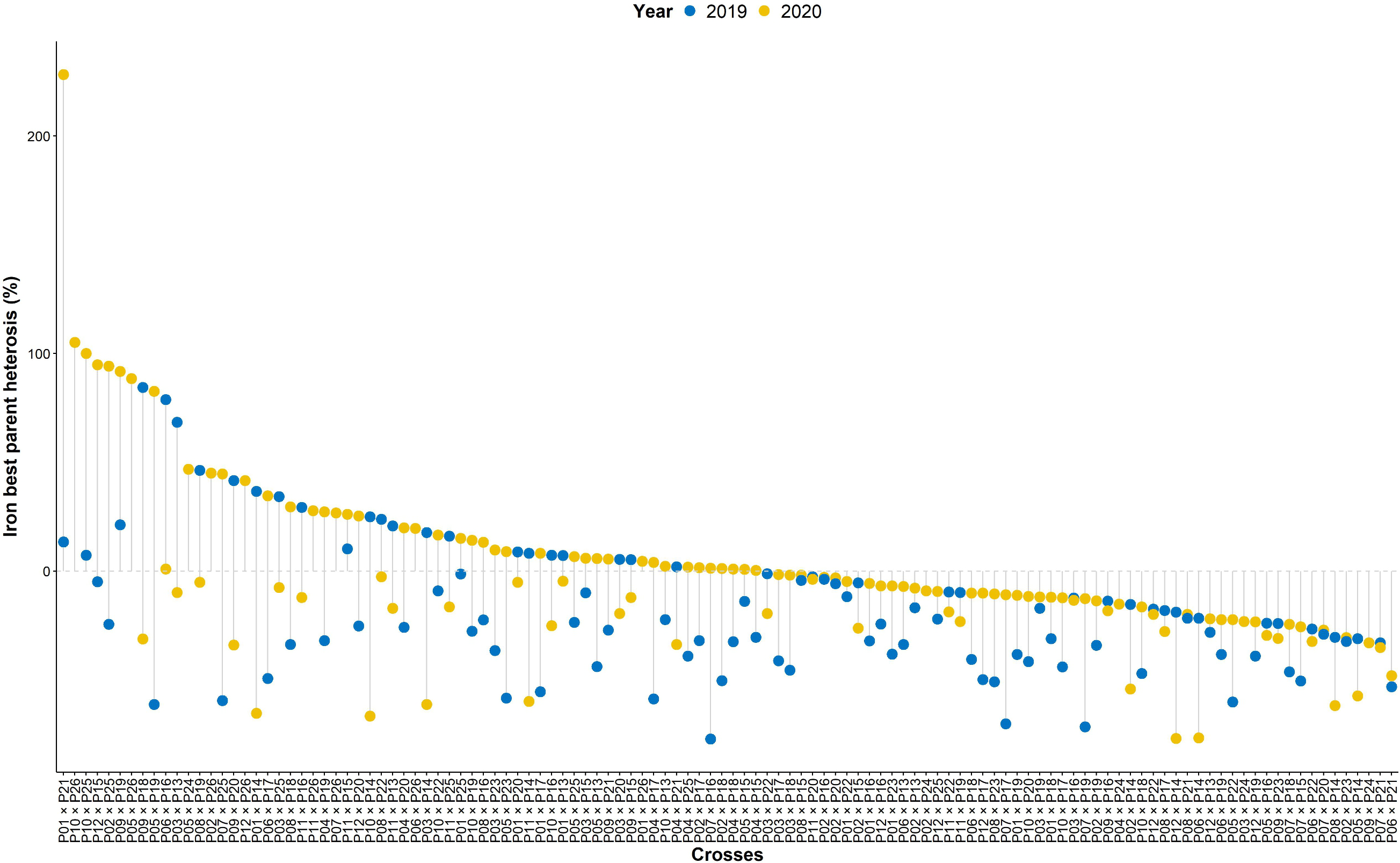

Supplement: S22 Fig — (TIF) [file pone.0332095.s024.tif]

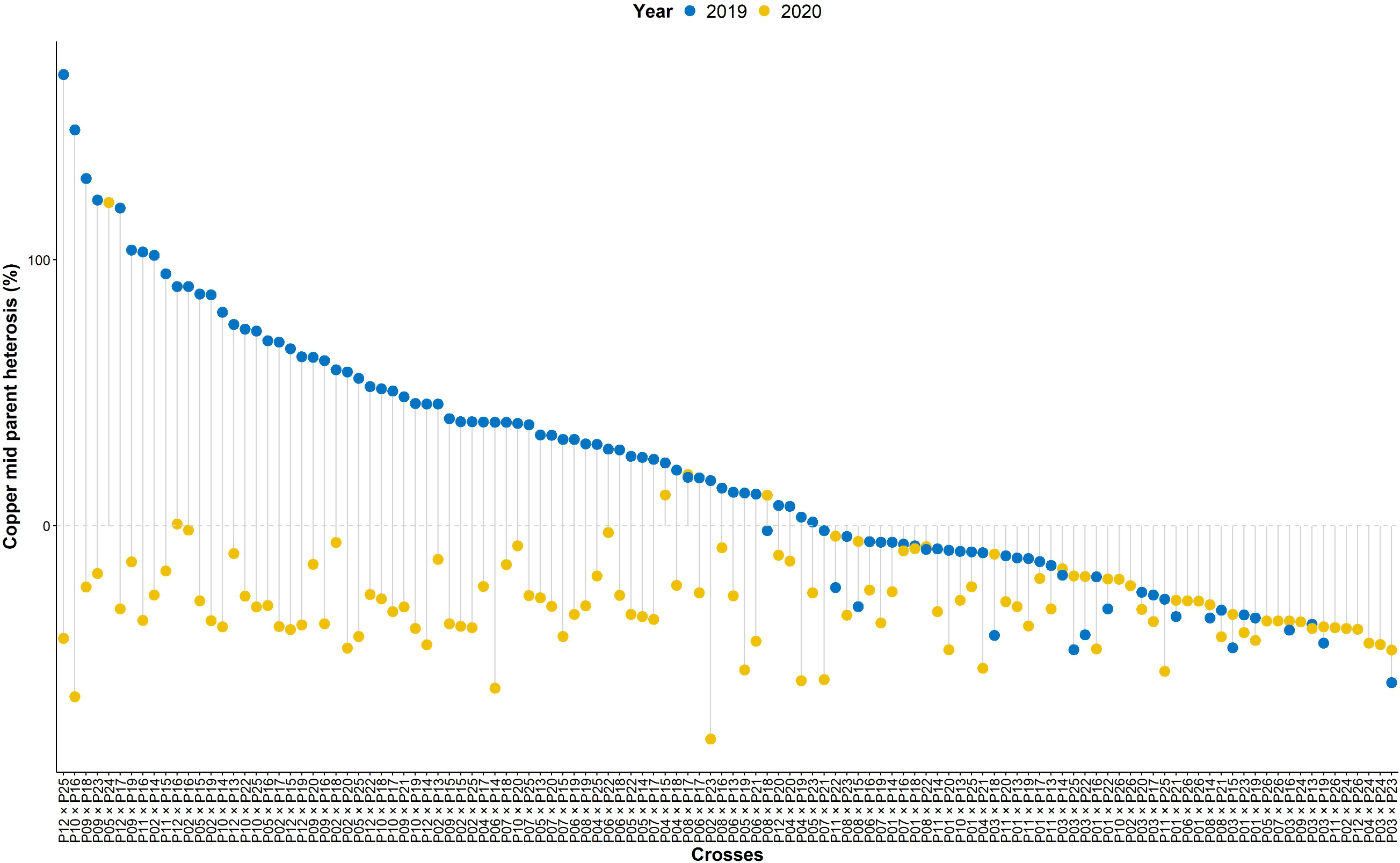

Supplement: S23 Fig — (TIF) [file pone.0332095.s025.tif]

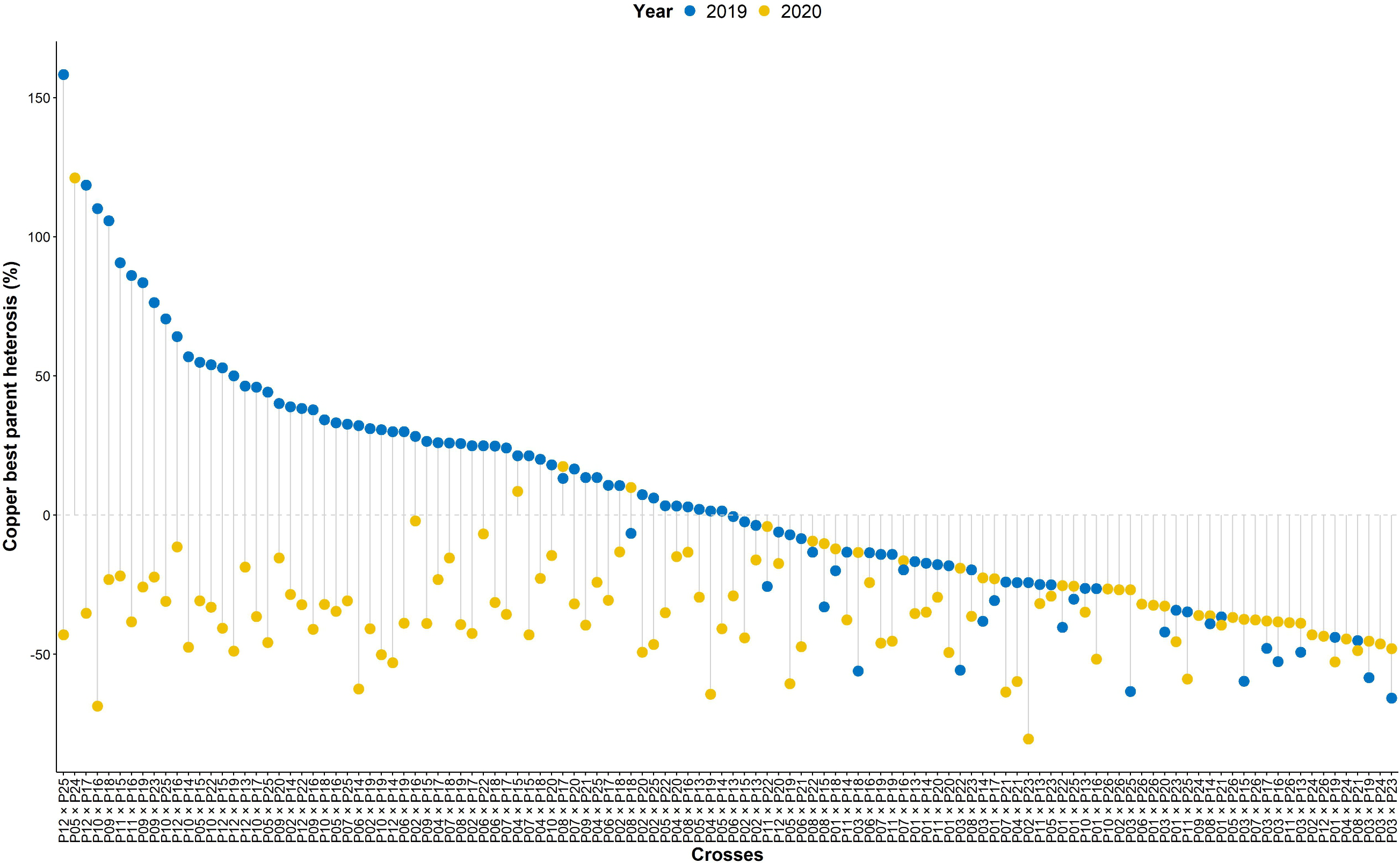

Supplement: S24 Fig — (TIF) [file pone.0332095.s026.tif]

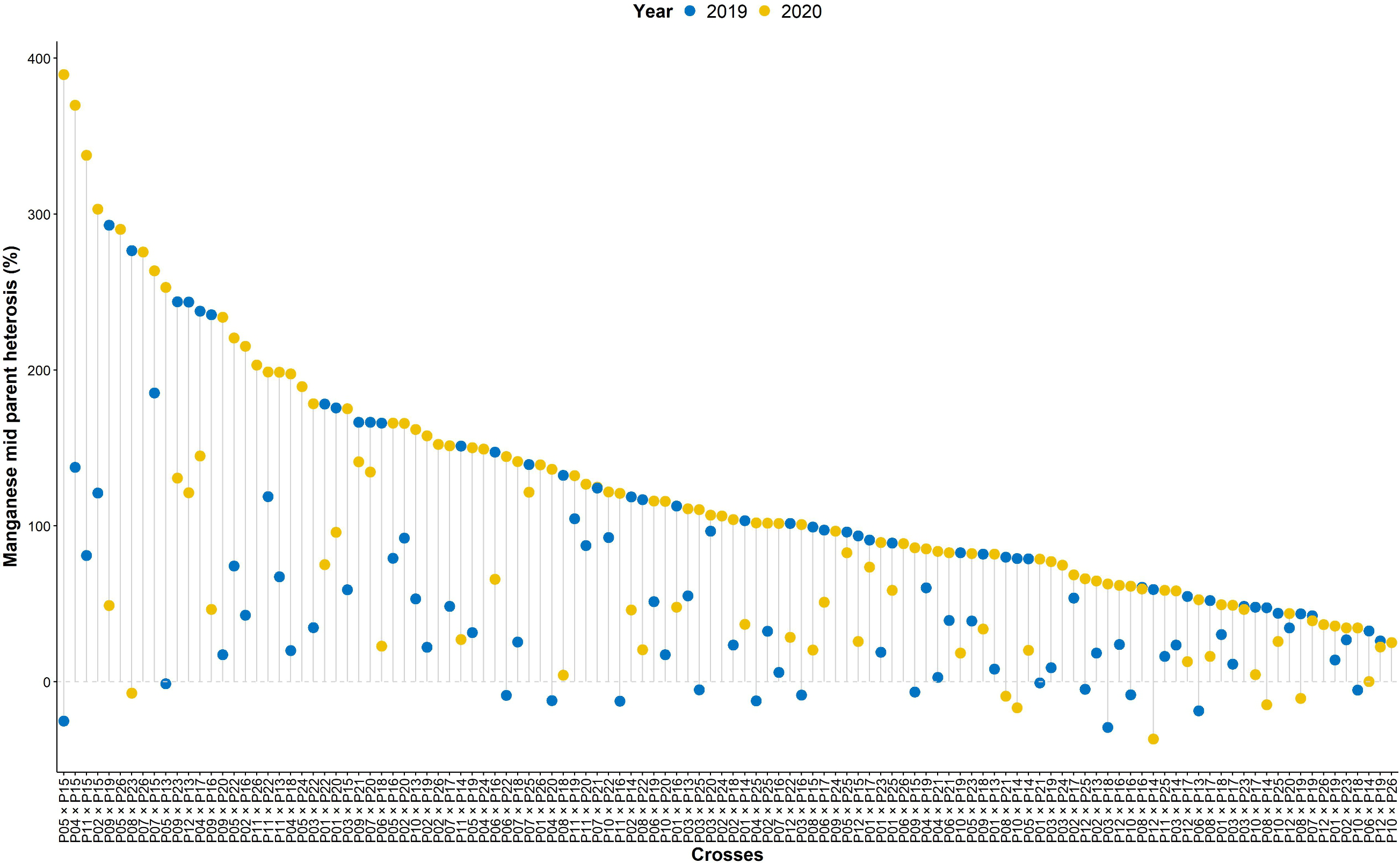

Supplement: S25 Fig — (TIF) [file pone.0332095.s027.tif]

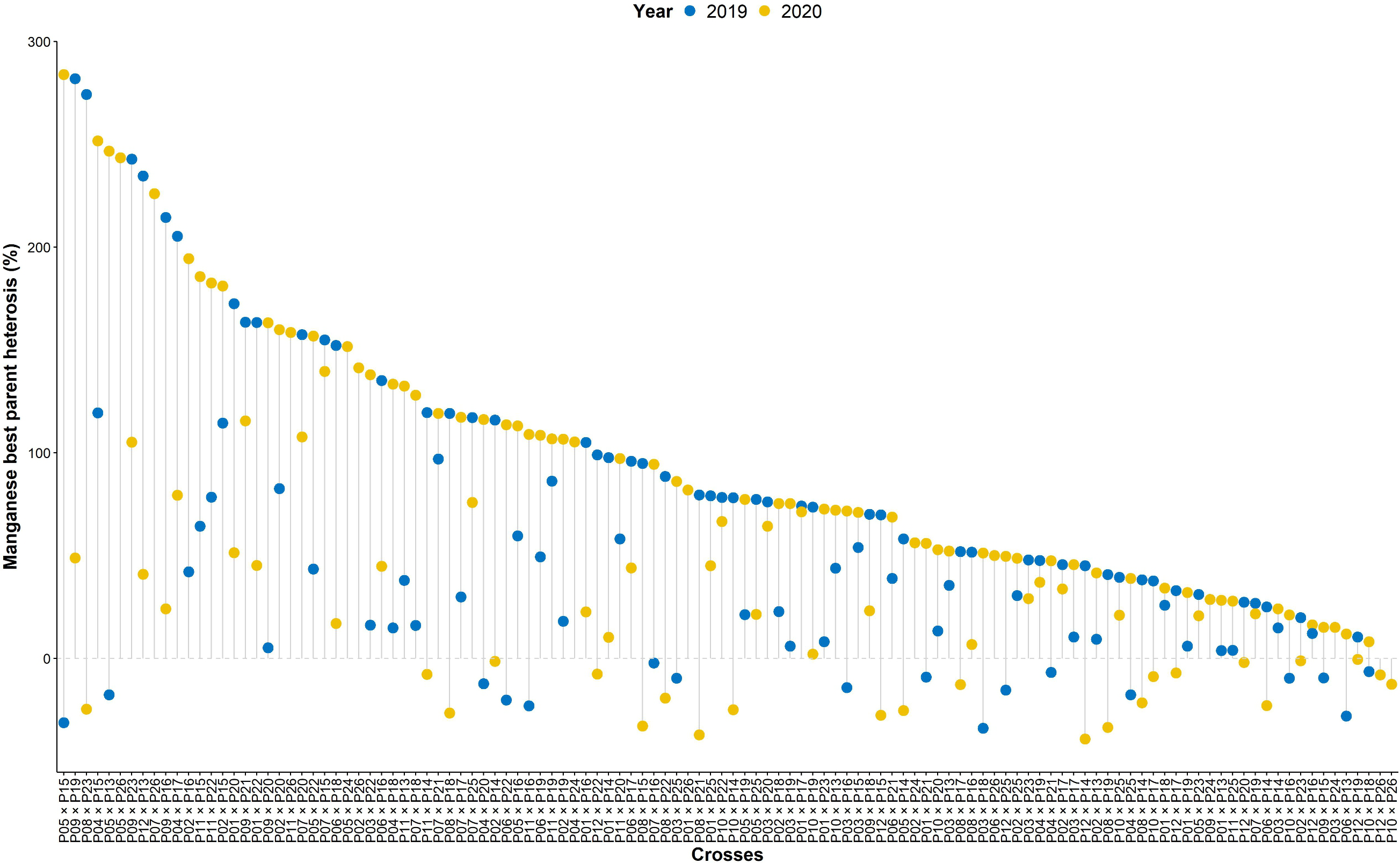

Supplement: S26 Fig — (TIF) [file pone.0332095.s028.tif]

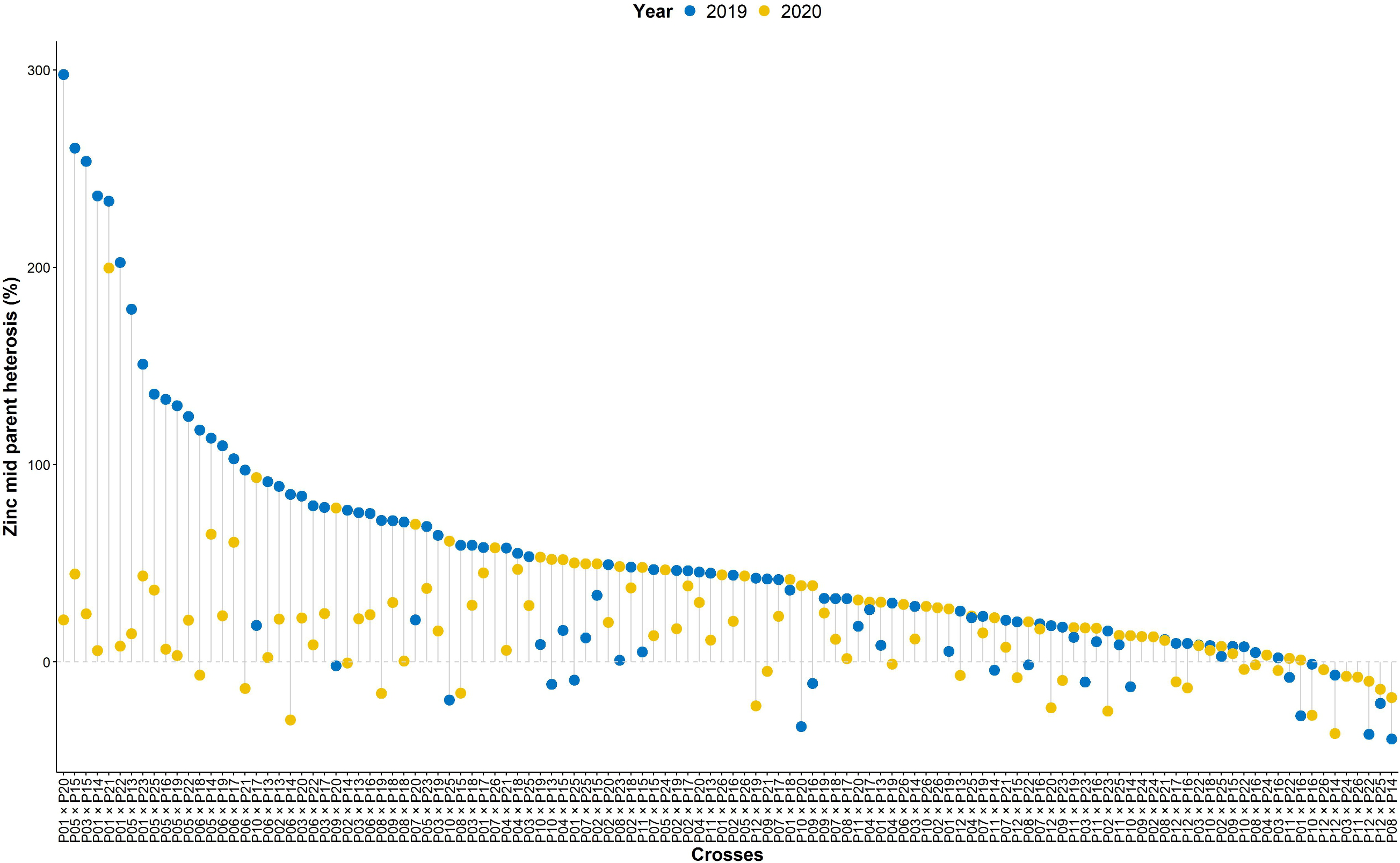

Supplement: S27 Fig — (TIF) [file pone.0332095.s029.tif]

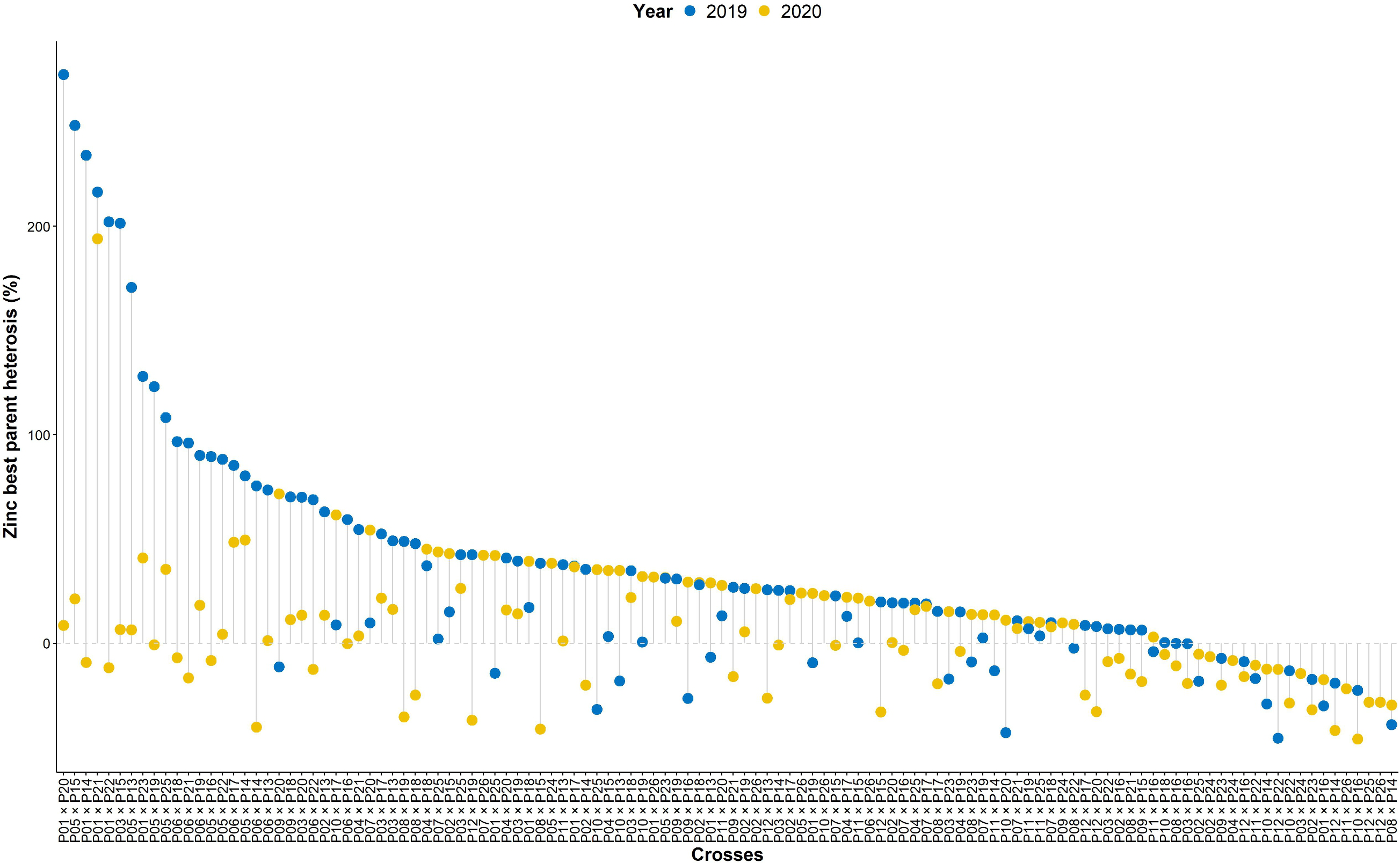

Supplement: S28 Fig — (TIF) [file pone.0332095.s030.tif]
