# Supplementary material for: Combining ability and heterosis analysis for mineral content in the leafy vegetable Gynandropsis gynandra (L.) Briq
Source: PLoS One. 2025 Sep 12;20(9):e0332095. doi: 10.1371/journal.pone.0332095 (PMC12431277; doi:10.1371/journal.pone.0332095)
Supplement: Attachment — Submitted filename: Responses to Reviewers.docx [file pone.0332095.s033.docx]

**Manuscript: PONE-D-25-18553**

**Combining ability and heterosis analysis for mineral content in the leafy vegetable *Gynandropsis gynandra* (L.) Briq.**

**Response to Editor and reviewers' comments**

We thank the editor and the reviewers for their valuable and helpful comments on the manuscript. We provided below a point-by-point response to their comments.

**Editor’s comments**

**Comment #1**. Please ensure that your manuscript meets PLOS ONE's style requirements, including those for file naming. The PLOS ONE style templates can be found at <https://journals.plos.org/plosone/s/file?id=wjVg/PLOSOne_formatting_sample_main_body.pdf> and <https://journals.plos.org/plosone/s/file?id=ba62/PLOSOne_formatting_sample_title_authors_affiliations.pdf>

**Answer #1**. We thank the editors and reviewers for their valuable comments. PLOS ONE’s style requirements were checked, and the revised manuscript was corrected accordingly.

**Comment #2**. We note that the grant information you provided in the ‘Funding Information’ and ‘Financial Disclosure’ sections do not match. When you resubmit, please ensure that you provide the correct grant numbers for the awards you received for your study in the ‘Funding Information’ section.

**Answer #2**. The information in the ‘Funding Information’ and ‘Financial Disclosure’ were crosschecked. The grant number for the award received for this study provided in the ‘Funding Information’ section was corrected to match the information in the ‘Financial Disclosure’.

**Reviewer #1**

**Overall comment:**

**Point#1.** The manuscript titled “Combining ability and heterosis analysis for mineral content in the leafy vegetable *Gynandropsis gynandra*” presents a comprehensive and technically robust study on the genetic analysis of mineral content using North Carolina Design II. The experimental design is appropriate, the dataset is extensive, and the statistical analyses have been conducted with rigor using REML-based mixed models in ASReml-R. The manuscript addresses an important area in plant breeding and nutrition, particularly for underutilized crops with high micronutrient value.

The findings are well-supported by the presented data. The authors successfully demonstrate the prevalence of non-additive gene action in the inheritance of mineral content and the significant potential of heterosis in improving these traits. The study provides practical insights for hybrid breeding strategies in *G. gynandra*. The manuscript is of publishable quality after minor revision. I would recommend that you accept the correction of typographical and referencing issues noted in the attached file, and confirm that all underlying data are publicly available in compliance with PLOS ONE policy. Please ensure that the revised version clearly addresses these points.

**Answer#1.** We thank the reviewer for his valuable and helpful comments on the manuscript. All the correction of typographical and referencing issues provided were accepted (**See the revised manuscript**).

We confirm that all underlying data are publicly available in compliance with PLOS ONE policy as all relevant data are within the manuscript and its supporting information files. The present study's dataset was uploaded as supporting information files (S2 Table). This was clearly mentioned in the **Data Availability Statement** as follows: **“Data Availability: All relevant data are within the manuscript and its Supporting Information files.”**

**Reviewer #2**

**Overall comment:** I have completed our review of your manuscript "Combining ability and heterosis analysis for mineral content in the leafy vegetable *Gynandropsis gynandra* (L.) Briq." While the research addresses an important topic in orphan crop improvement, several major revisions are needed to strengthen the manuscript's impact and scientific rigor.

**Point#2.1-** **Introduction:** Add specific data on impact potential, compare to other biofortification efforts, and better justify why this crop deserves genetic research investment compared to established biofortification programs.

**Answer#2.1:** We thank the reviewer for this suggestion. Data on impact potential, compararison to other biofortification efforts, and justification of why this crop deserves genetic research investment compared to established biofortification programs were included in **the revised manuscript with track changes** (see **lines** **92-104**) as follows:

**“More importantly, *G. gynandra* leaves have 4.7- and 3.2-fold of vitamin C, 4- and 2-fold iron, 1.2- and 2.1-fold zinc, 2.7- and 10.4-fold calcium, 3.3- and 5.5-fold phosphorus, 1.4- and 1.8-fold potassium, 2.59 and 1.37-total phenolics, and 5.70- and 2.46-fold flavonoids concentrations higher than two world leading commercial and consumed vegetables namely cabbage (*Brassica oleracea* var. *capitata* cv. Drumhead) and Swiss chard (*Beta vulgaris* L. cv. Fordhook Giant), respectively [1]. Breeding efforts in *G. gynandra* will result in significant impacts on healthier and balanced diets for local communities in Africa and Asia where the species is mostly consumed [2, 3] and high micronutrient deficiencies occurs. Moreover, *G. gynandra* is a climate resilient crop as it is a C_4_ plant with the ability to withstand various harsh conditions [3] ; the species was used as a model crop to understand C_4_ traits, evolution and gene expression in plants [4-6]. The production and the exploitation of the nutritional potential of *G. gynandra* like many other opportunity crops are limited due to lack of improved varieties resulting from the lack of sustainable breeding program.”**

**Point#2.2** **Introduction:** Define GCA/SCA and heterosis concepts clearly for broader readership, discuss breeding implications of the outcrossing nature, and connect to related Brassicaceae breeding work to provide context.

**Answer#2.2.** We have edited the **revised manuscript with track changes** (see **lines 105-143**) as follows:

**“Breeding strategies and type of cultivars for a given crop species are guided by the species reproductive biology and mating systems. Both self- and cross-compatibility occurs in *G. gynandra* with outcrossing being predominant [7-9], offering the possibility for developing both hybrids and inbred cultivars. Because of the predominance of outcrossing, the species might exhibit heterosis and the choice for developing hybrids cultivars sounds as good strategy to exploit the potential heterosis that needs to be properly documented. Heterosis or hybrid vigour refers to the outperformance of the first generation of progenies compared to their parents [10-13] and has been highly researched by breeders for open-pollinated crops but also for self-pollinated crops for higher productivity. Four steps are crucial in the development of a hybrid cultivar: (i) the establishment of populations for selection; (ii) inbred lines development; (iii) inbred lines’ evaluation for combining ability; and (iv) the production of hybrid seed [14]. Following the development of the inbred/advanced lines in *G. gynandra* [15, 16], their testing for combining ability is the next step towards hybrids creation. Combining ability refers to the ability of a line to combine with another one during hybridization so that desirable genes or traits are transferred to their progenies and encompasses two types, namely the general combining ability (GCA) and the specific combining ability (SCA) [17]. According to Sprague and Tatum [17], a GCA of a line is the average performance of this line in a set of its hybrid combinations and the SCA is the deviation of a specific cross performance from the sum of the average performance or GCA of its parental lines. Combining ability informs on the nature and magnitude of gene action controlling the considered trait. GCA are associated with additive genes effects, while the SCA effects are attributed to nonadditive gene action, including dominance and epistasis gene effects [17]. The GCA helps in the selection of good parents for breeding and the SCA is useful for the selection of the best hybrid combination to better exploit heterosis. The assessment of the combining ability of a set of lines and the inheritance patterns for a target species is done using mating designs such as North Carolina Design II, diallel and line by tester, among others [18-20].**

***G. gynandra* belongs to Cleomaceae family, a sister family of Brassicaceae and could gain more from breeding strategies implemented in *Brassica* crops. Most developed varieties for *Brassica* crops nowadays are hybrids and the evaluation of combining ability, heterosis level and gene action has been intensively done for several parents in *Brassica oleracea* var. *capitata* [21], *Brassica* *rapa* L. [22], and *Brassica* *oleracea* var. *botrytis* L. [23, 24] for nutritional traits such as minerals, antioxidant pigments, and vitamins. Unfortunately, to the best of our knowledge, the heterosis level and the combining ability potential for mineral content in *G. gynandra* are yet to be investigated to inform breeding program of good parents, heterotic crosses and breeding methods for improved cultivars development.”**

**Point#2.3.** **Introduction:** Replace vague objectives with measurable ones (e.g., "quantify heterosis magnitude" vs "determine gene action"), add missing hypotheses about correlations and stability, and connect each objective to practical breeding decisions.

**Answer#2.3:** The objectives were replaced with measurable ones that are connected to practical breeding decisions. The revised objectives were included in **the revised manuscript (lines 147-154)** as follows:

**“Specifically, the study (i) compared the leaf mineral content of 118 experimental F1 *G. gynandra* hybrids and their parental lines; (ii)** **quantified heterosis magnitude for leaf mineral content in *G. gynandra* to select the most suitable breeding method; (iii) evaluated the combining ability effects for leaf mineral content of the parental lines of *G. gynandra*** **to identify good parents and best crosses; and (iv) estimated the extent of association between mean performance, heterosis and combining ability for mineral content in *G. gynandra*** **to guide prediction of hybrid performance and multiple traits selection.”**

The missing hypotheses about correlations and stability were now included in **the revised manuscript with track changes (lines 164-166)** as follows:

**“(v) the leaf mineral elements content in *G. gynandra* are correlated; and (vi) the correlations between leaf mineral elements content are stable from parents to hybrids.”**

**Point#2.4.** **Introduction:** The current single-sentence gap statement is too brief and narrow, only mentioning that "no information exists for *G. gynandra*." This fails to justify why this research matters beyond academic curiosity and should be rewritten to demonstrate clear translational impact potential.

**Answer#2.4.** The gap statement was revised and could been seen in the introduction section. Please see **lines 102-143 of the revised manuscript with track changes**.

**Point#2.5 Materials and Methods-** Justify experimental design choices: Why were specific lines assigned as females vs. males? This affects interpretation of GCA estimates and should be explicitly explained.

**Answer#2.5.** Previous studies of the reproductive biology revealed that most individual plants of *G. gynandra* are andromonoecious, producing both staminate and fertile hermaphrodite floral types [7, 8]. The proportion of each flower type on individual plant varied from one population to another, ranging from 48 to 70% for staminate flowers and 30 to 52% for fertile hermaphrodite flowers [7, 8]. Therefore, parental lines with abundant fertile hermaphrodite flowers were considered as females and those with abundant staminate flowers were considered as males. This information is now included in **the revised manuscript with track changes (lines 173-180)** as follows:

**“The lines were separated into two groups; 12 and 14 lines used as females and males, respectively,** **based on the male/staminate flowers/pollen and female/hermaphrodite flowers production ability (Table 1) as most individual plants in the species were reported to be andromonoecious with differential flowers productivity [7, 8].** **Males were crossed with females in a North Carolina design II** **during two summer seasons (season 1, from October 2018 to February 2019 and season 2, from October 2019 to March 2020) in a greenhouse at the Controlled Environment Facility (29°46′ S, 30°58′ E) of the University of KwaZulu-Natal, Pietermaritzburg Campus, South Africa.”**

**Point#2.6**- **Materials and Methods:** Address incomplete factorial: Only 118 of 168 possible crosses succeeded. Explain selection criteria and how this affects design balance and statistical power.

**Answer#2.6:** The crossings between the 12 females and 14 lines males were expected to generate 168 single crosses, but only 118 crosses had sufficient amount of seeds for evaluation. Therefore, the only criteria used was the hybrid seed quantity for planting. This resulted in an incomplete factorial mating design. Luckily, Keuls and Garretsen [25] and Garretsen and Keuls [26] provided a comprehensive framework for the analysis of genetic variation in complete and incomplete diallels and North Carolina II designs. However, their analysis was based on analysis of variance. Here we employed linear mixed-effect model (LMM), a modification of the linear model of ANOVA, due to its advantages over the ANOVA models [27]. Firstly, LMM allows using both fixed and random effects, while ANOVA is employed for only fixed effects. Secondly, a linear mixed effects model provides better results as it handle well missing data, if any. Moreover, LMM provides information not only on the significant effect of phenotypic variation but also on the replication/block. All the factors were set as random, the likelihood ratio test [28] was used to test their significance. This approach has been used in several studies involving incomplete factorial mating design for various crop species, including tomato [29], maize [30], wheat [31-35], sorghum [36].

**Point#2.7**- **Materials and Methods**: Improve sampling protocols: The "random collection" of leaf samples needs more specific description (which leaves, plant age, time of day, etc.) for reproducibility.

**Answer#2.7:** Leaf samples were collected from plants four weeks after transplanting, meaning eight weeks old plants from sowing date. Tenders and edible leaves were randomly picked from plants in the morning between 08:00 am and 11:00 am. This information was included in the revised manuscript as follows **(lines 227-229 of the revised manuscript)**:

**“A sample of 20 g of tenders and edible fresh leaves per genotype was randomly collected from all the plants in each replicate in paper bags four weeks after transplanting, meaning eight weeks old plants from sowing date. The leaves were picked from plants between 08:00 and 11:00 am.”**

**Point#2.8**- **Materials and Methods:** Enhance environmental documentation: Provide more detailed greenhouse conditions (daily temperature ranges, humidity fluctuations, light levels) as these affect mineral content.

**Answer#2.8:** The greenhouse structure was made of corrugated polycarbonate material. The polycarbonate material that covered the greenhouse reduced the outside solar irradiance by an average 50%. Therefore, the maximum of solar radiation was 356 and 567 W m^-2^. In 2019, the day temperature ranged between 20 and 35°C and the night temperature ranged between 15 and 25°C. At the same time, the relative humidity was between 75 and 87% at night and between 45 and 70% at day hours. In 2020, while the day temperature ranged between 25 and 38°C and the night temperature ranged between 20 and 25°C. The day relative humidity was between 50 and 80% and between 80 and 90% at night. The hourly day and night variations in temperature, relative humidity fluctuations and solar radiation in the under the greenhouse were presented in S1 Fig.

This information was included in **the revised manuscript (lines 218-225)** as follows:

**“****The greenhouse structure was made of corrugated polycarbonate material, which reduced the outside solar irradiance by 50% on average. Therefore, the maximum of solar radiation was 356 and 567 W m^-2^ in 2019 and 2020, respectively. In 2019, the day temperature varied between 20 and 35°C and the night temperature ranged from 15 to 25°C (S1 Fig). At the same time, the relative humidity was between 75 and 87% at night and 45 and 70% during day hours. In 2020, while the day temperature ranged from 25 and 38°C and the night temperature ranged between 20 and 25°C. The day relative humidity was between 50 and 80% and between 80 and 90% at night (S1 Fig).”**

**S1 Fig. Variations in temperature, relative humidity and solar radiation under the greenhouse in 2019 and 2020.** (A) Temperature in 2019. (B) Temperature in 2020. (C) Relative humidity in 2019. (D) Relative humidity in 2020. (E) Solar radiation in 2019. (F) Solar radiation in 2020.

**Point#2.9**- **Results:** Since G×Y variance > genotypic variance, present results by year separately rather than just combined, as the current approach may be masking important patterns.

**Answer#2.9:** This is correct. Because G×Y variance > genotypic variance, results by year were presented separately (**see all Figures and Tables 4, 5 and 6**) and was also mentioned in the manuscript (**lines 404-407 of the revised manuscript with track changes) as follows:**

**“Given the significant effect of year and its interaction with genotype and combining abilities, estimates of genetic variance components, heritability estimates, degree of dominance, Baker’s [41] predictability ratio, and genetic advance were estimated in each year and summarized for macroelements (Ca, Mg, Na, K, P) in Table 4 and microelements (Fe, Mn, Cu, Zn) in Table 5.”**

**Point#2.10**- **Results:** Address how the substrate change (soil vs. growing medium) affects the trustworthiness of combining ability estimates and breeding recommendations.

**Answer#2.10:** The substrate change (soil vs. growing medium) is very important in evaluating and confirming the combining abilities estimates and breeding recommendations. In fact, a consistency observed in the combining ability estimates across substrates confirm the trustworthiness of the results.

**Point#2.11**- **Results:** Perhaps the "year" effects are actually substrate effects. Discuss what this means for practical applications and generalizability.

**Answer#2.11:** Well, the year effect might be associated with the substrate effects. This has been discussed in the discussion section, **lines 646-668 of the revised manuscript**.

**Point#2.12**- **Results:** Many figures are difficult to interpret. Use clearer labeling, better color schemes, and more intuitive layouts to enhance accessibility.

**Answer#2.12.** Figures were revised according to PLOS One recommendations (https://journals.plos.org/plosone/s/figures).

**Point#2.13**- **Results:** Don't just report statistical significance. Discuss what effect sizes mean biologically and for each major finding, explain what it means for breeding strategy and variety development.

**Answer#2.13:** Each major finding was discussed in the Discussion section to provide biological meaning and its implication for breeding strategy and variety development. Please refers to Discussion section of the manuscript.

**Point#2.14**- **Discussion:** Can results from controlled greenhouse conditions with artificial substrates be applied to field breeding programs? This limitation needs explicit discussion.

**Answer#2.14:** Results from controlled greenhouse conditions with artificial substrates may not necessary be straightforward translated into field conditions. However, Poorter et al. [37] provided several avenues so that results from controlled envrionments could be applied to field conditions. The proposed avenues included: growing plants under exactly the same conditions as out-side; growing plants with conditions that change between days; the use of soils and mimics environmental conditions. In our case, soil was also used in addition to growing media. For light supply, we relied on the sunlight though its intensity was recuded on average by 50% in the greenhouse, its daily variation was consistent with the outdoor one in order to allow the plants to experience a probable daily light intensity variation. Moreover, in another study, which combined analysis of greenhouse and field grown hybrids of *G. gynandra* for combining ability for biomass and related traits, the analysis led to similar results in field and greenhouse. However, evaluation under field conditions is required. This was included in the manuscript (**lines 609-611 of the revised manuscript with track changes)** as follows :

**“Further investigations are therefore required to assess the phenotypic plasticity and stability of the newly developed hybrids, identifying the best genotypes under various agroecological and agronomic practices in field conditions.”**

**Point#2.15**- **Discussion:** The strong Ca-Mg correlation deserves deeper mechanistic explanation beyond just mentioning "pleiotropic loci" - discuss potential physiological and molecular mechanisms.

**Answer#2.15:** Thanks for this suggestion. Potential physiological and molecular mechanisms contributing to the obrseved strong Ca-Mg correlation are now included in the discussion section of the revised manuscript (**lines 792-809 of the revised manuscript)** as follows:

**“****Moreover, this strong association could be explained by the antagonistic interaction between calcium and magnesium in plant cells [38, 39]. Consequently, a homeostatic balance between calcium and magnesium ions are important for plant development and growth [38, 40]. While calcium play a crucial role for cytosolic signalling, membrane and cell wall integrity [41], magnesium is a key constituent of chlorophyll, essential for photosynthesis, cofactor for enzymes, protein synthesis and energy metabolism [42]. Though calcium and magnesium have different biochemical and physiological roles in plants, homeostasis of calcium and magnesium ions appeared to be closely linked and at least partly regulated by common signaling networks [38]. Specifically, Ca^2+^ signaling participate in regulating the dynamic homeostasis of Mg^2+^ [38]. Therefore, it was suggested that high external magnesium ions would lead to a transient rise in cytosolic calcium ions in plant cells. In case of excess magnesium, the Ca^2+^ signal is detected by two tonoplast-localized calcineurin B-like (CBL) proteins (CBL2 and CBL3), which concomitantly activate four of CBL-interacting protein kinases (CIPKs) (CIPK3, 9, 23 and 26). This CBL–CIPK complex, therefore, recruits Mg^2+^ and regulate downstream specific transporters in the tonoplast for efficient sequestration of Mg^2+^ in vacuoles, thereby ensuring a non-toxic level of Mg^2+^ in the cell as a protective mechanism [43]. This opens roons for the use of *G. gynandra* to uncover the physiological and regulatory mechanism of calcium and magnesium homeostasis in plants.”**

**Point#2.16**- **Discussion:** How would hybrid breeding actually work for this outcrossing species? Discuss male sterility systems, seed production logistics, and economic feasibility.

**Answer#2.16:** This was discussed and added to the revised manuscript (**lines 831-842 of the revised manuscript**) as follows:

**“As this study pointed out that hybrid cultivars will help improve the species productivity, the establishment of hybrid breeding will be beneficial. However, for its this might face some challenges, such as the cost-effectiveness of the breeding program and the willingness of farmers to buy hybrid seeds as hybrids seeds are often expensive compared with pures lines or open pollinated cultivars. Given the competitive nutritional advantages of *G. gynandra* over some world leading *Brassica* vegetables [1], a proper advocacy and rising awareness could boost the species demand across Africa and the globe. The success of the development of hybrid cultivars for spider plant will require (i) an investigation of male sterility to ease the hybrid production, (ii) the evaluation of the cost-effectiveness of hybrid cultivars and (iii) the establishment of seed production logistics as vegetable seed system in Africa is still dominated by informal system. Despite these challenges, experience from Brassicaceae species, a sister family to Cleomaceae family, which *G. gynandra* belongs to could be useful.”**

**Point#2.17**- **Discussion:** How do these results compare to successful biofortification programs (iron beans, zinc wheat, vitamin A crops)? What can be learned from those experiences?

**Answer#2.17:** Huey et al. [44] presented a holistic review of the facilitators and barriers to adoption of biofortified foods and food products, which were found to be crop specific. Irrespective to crop, enablers included awareness of benefits/knowledge about importance/relative advantage, efficient production and management practices, access to information, farmers’ networking and high farming experience. Barriers included lacking timely access to quality planting materials and market remoteness, poor road networks and scarcity of improved technology including inadequate processing/storage facilities, low knowledge of biofortification techniques, low awareness and concerns regarding productivity and quality. Similarly, Ahoudou et al. [45] pointed out that adoption factors of the orange-fleshed sweetpotato in sub-Saharan Africa included: (i) knowledge and awareness; (ii) agronomic traits; (iii) social factors and networks; (iv) taste and sensory attributes; (v) market and economic considerations and (vi) health and nutrition. Based on these findings, the implications for the hybrid breeding program of *G. gynandra* for better adoption of potential cultivars were discussed and included in the manuscript (**lines 842-850 of the revised manuscript**) as follows:

**“The implementation of hybrid breeding program should consider a holistic approach that consider all stakeholders along the crop value chains. Therefore, actions could include: (i) the evaluation of best parents and crosses for agronomic traits and adapatatility though participatory breeding and tricot citizen science taking into account social factors and networks; (ii) farmers training on good agronomic practices; (iii) the development of seed system to ensure permanent seed availability; and (iv) the assessment of market acceptability, the taste and sensory attributes. At the same time, raising awarenesss and knowledge sharing of nutritional and health benefits of the species activities should be implemented to stimulate demand for market creation.”**

**Point#2.18- Discussion:** Address unique constraints of breeding underutilized species vs. major crops, including market development, value chains, and resource limitations.

**Answer#2.18:** Contraints of breeding underutilized species have been documented in literature. These constraints might be crop specific given that all neglected crops are not at the same level of domestication, including genetic improvement. Specifically, a roadmap for breeding orphan leafy vegetable with focus on our target species, *G. gynandra*, has already been developed by Sogbohossou et al. [2]. More importantly, progress in the species breeding over the last decade has been developed by Achigan-Dako et al. [46] with details on the way forward for speeding up the breeding of the species, enhanced consusumption as well as strategies for market development, value chains and valorisation in industrial food and drug production.

**References**

1.	Moyo M, Amoo SO, Aremu A, Gruz J, Šubrtová M, Jarošová M, et al. Determination of mineral constituents, phytochemicals and antioxidant qualities of *Cleome gynandra*, compared to *Brassica oleracea* and *Beta vulgaris*. Front Chem. 2018;5:128. doi: 10.3389/fchem.2017.00128.

2.	Sogbohossou EOD, Achigan-Dako EG, Maundu P, Solberg S, Deguenon EMS, Mumm RH, et al. A roadmap for breeding orphan leafy vegetable species: a case study of *Gynandropsis gynandra* (Cleomaceae). Hortic Res. 2018;5(1):2. doi: 10.1038/s41438-017-0001-2.

3.	Mashamaite CV, Manyevere A, Chakauya E. Cleome gynandra: A wonder climate-smart plant for nutritional security for millions in semi-arid areas. Front Plant Sci. 2022;13:1003080. doi: <https://doi.org/10.3389/fpls.2022.1003080>.

4.	Reeves G, Singh P, Rossberg TA, Sogbohossou EOD, Schranz ME, Hibberd JM. Natural variation within a species for traits underpinning C4 photosynthesis. Plant Physiol. 2018;177(2):504. doi: 10.1104/pp.18.00168.

5.	Hoang NV, Sogbohossou EOD, Xiong W, Simpson CJC, Singh P, Walden N, et al. The Gynandropsis gynandra genome provides insights into whole-genome duplications and the evolution of C4 photosynthesis in Cleomaceae. Plant Cell. 2023;35(5):1334-59. doi: 10.1093/plcell/koad018.

6.	Singh P, Stevenson SR, Dickinson PJ, Reyna-Llorens I, Tripathi A, Reeves G, et al. C4 gene induction during de-etiolation evolved through changes in cis to allow integration with ancestral C3 gene regulatory networks. Sci Adv. 2023;9(13):eade9756. doi: 10.1126/sciadv.ade9756.

7.	Zohoungbogbo HPF, Houdegbe CA, Sogbohossou DEO, Tossou MG, Maundu P, Schranz EM, et al. Andromonoecy in *Gynandropsis gynandra* (L.) Briq. (Cleomaceae) and effects on fruit and seed production. Genet Resour Crop Evol. 2018;65(8):2231–9. doi: 10.1007/s10722-018-0687-5.

8.	Raju AS, Rani DS. Reproductive ecology of *Cleome gynandra* and *Cleome viscosa* (Capparaceae). Phytol Balcan. 2016;22(1):15-28.

9.	Omondi EO, Debener T, Linde M, Abukutsa-Onyango M, Dinssa FF, Winkelmann T. Mating biology, nuclear DNA content and genetic diversity in spider plant (*Cleome gynandra*) germplasm from various African countries. Plant Breed. 2017;136:578-89. doi: 10.1111/pbr.12485.

10.	Shull GH. The Composition of a Field of Maize. J Hered. 1908;os-4(1):296-301. doi: 10.1093/jhered/os-4.1.296.

11.	Hochholdinger F, Baldauf JA. Heterosis in plants. Curr Biol. 2018;28(18):R1089-R92. doi: 10.1016/j.cub.2018.06.041.

12.	Darwin C. The effects of cross and self fertilisation in the vegetable kingdom: D. Appleton; 1877.

13.	Shull GH. What is" heterosis"? Genetics. 1948;33(5):439.

14.	Fehr WR, Suza WP. Principles of cultivar development. Ames, Iowa, United States of America: Iowa State University Digital Press; 2025 March 28, 2025. 81 p.

15.	Houdegbe AC, Achigan-Dako EG, Sogbohossou EOD, Schranz ME, Odindo AO, Sibiya J. Leaf elemental composition analysis in spider plant [*Gynandropsis gynandra* L. (Briq.)] differentiates three nutritional groups. Front Plant Sci. 2022;13:841226. doi: 10.3389/fpls.2022.841226.

16.	Houdegbe AC, Achigan-Dako EG, Sogbohossou EOD, Schranz ME, Odindo AO, Sibiya J. Phenotypic variation in biomass and related traits among four generations advanced lines of Cleome (*Gynandropsis gynandra* L. (Briq.)). PLoS One. 2022;17(10):e0275829. doi: 10.1371/journal.pone.0275829.

17.	Sprague GF, Tatum LA. General vs. Specific Combining Ability in Single Crosses of Corn. Agron J. 1942;34(10):923-32. doi: 10.2134/agronj1942.00021962003400100008x.

18.	Nduwumuremyi A, Tongoona P, Habimana S. Mating designs: helpful tool for quantitative plant breeding analysis. J Plant Breed Genet. 2013;1(3):117–29.

19.	Beavis W, Lamkey K, Mahama AA, Suza W. Mating Designs. In: Suza WP, Lamkey KR, editors. Quantitative Genetics for Plant Breeding: Iowa State University Digital Press; 2023.

20.	Hallauer AR, Carena MJ, Miranda Filho Jd. Quantitative genetics in maize breeding. London: Springer Science & Business Media; 2010.

21.	Singh BK, Sharma SR, Singh B. Heterosis for mineral elements in single cross-hybrids of cabbage (*Brassica oleracea* var. *capitata* L.). Sci Hortic. 2009;122(1):32-6. doi: 10.1016/j.scienta.2009.04.007.

22.	Xie F, Zha J, Tang H, Xu Y, Liu X, Wan Z. Combining ability and heterosis analysis for mineral elements by using cytoplasmic male-sterile systems in non-heading Chinese cabbage (*Brassica rapa*). Crop Pasture Sci. 2018;69(3):296-302. doi: 10.1071/CP17357.

23.	Ram H, Dey SS, Gopala Krishnan S, Kar A, Bhardwaj R, Arun Kumar MB, et al. Heterosis and Combining Ability for Mineral Nutrients in Snowball Cauliflower (*Brassica oleracea* var. *botrytis* L.) Using Ogura Cytoplasmic Male Sterile Lines. Proc Natl Acad Sci, India, Sect B Biol Sci. 2018;88(4):1367-76. doi: 10.1007/s40011-017-0874-8.

24.	Karigar GP, Singh S, Mangal M, Saroha S, Saini N, Ray M, et al. Dietary micronutrient content, heterosis and combining ability for breeding mineral-rich hybrids in early- and mid-maturity groups of Indian cauliflower. Sci Hortic. 2023;312:111848. doi: 10.1016/j.scienta.2023.111848.

25.	Keuls M, Garretsen F. A general method for the analysis of genetic variation in complete and incomplete diallels and North Carolina II designs. Part I: Procedures and general formulas for the random model. Euphytica. 1977;26(3):537-51. doi: 10.1007/BF00021679.

26.	Garretsen F, Keuls M. A general method for the analysis of genetic variation in complete and incomplete diallels and North Carolina II (NC II) designs. Part II. Procedures and general formulas for the fixed model. Euphytica. 1978;27(1):49-68. doi: 10.1007/BF00039119.

27.	Mathews KL, Crossa J. Experimental Design for Plant Improvement. In: Reynolds MP, Braun H-J, editors. Wheat Improvement: Food Security in a Changing Climate. Cham: Springer International Publishing; 2022. p. 215-35.

28.	Self SG, Liang K-Y. Asymptotic Properties of Maximum Likelihood Estimators and Likelihood Ratio Tests under Nonstandard Conditions. J Am Stat Assoc. 1987;82(398):605-10. doi: 10.1080/01621459.1987.10478472.

29.	Ayenan MAT, Danquah A, Hanson P, Asante IK, Danquah EY. Combining abilities and heterotic patterns for heat tolerant traits in tomatoes (Solanum lycopersicum). Plant Breeding. 2022;141(4):585-97. doi: <https://doi.org/10.1111/pbr.13037>.

30.	Lorenzi A, Bauland C, Mary-Huard T, Pin S, Palaffre C, Guillaume C, et al. Genomic prediction of hybrid performance: comparison of the efficiency of factorial and tester designs used as training sets in a multiparental connected reciprocal design for maize silage. Theor Appl Genet. 2022;135(9):3143-60. doi: 10.1007/s00122-022-04176-y.

31.	Longin CFH, Gowda M, Mühleisen J, Ebmeyer E, Kazman E, Schachschneider R, et al. Hybrid wheat: quantitative genetic parameters and consequences for the design of breeding programs. Theor Appl Genet. 2013;126(11):2791-801. doi: 10.1007/s00122-013-2172-z.

32.	Schwarzwälder L, Thorwarth P, Zhao Y, Reif JC, Longin CFH. Hybrid wheat: quantitative genetic parameters and heterosis for quality and rheological traits as well as baking volume. Theor Appl Genet. 2022;135(4):1131-41. doi: 10.1007/s00122-022-04039-6.

33.	Gowda M, Longin CFH, Lein V, Reif JC. Relevance of Specific versus General Combining Ability in Winter Wheat. Crop Sci. 2012;52(6):2494-500. doi: <https://doi.org/10.2135/cropsci2012.04.0245>.

34.	Boeven PHG, Zhao Y, Thorwarth P, Liu F, Maurer HP, Gils M, et al. Negative dominance and dominance-by-dominance epistatic effects reduce grain-yield heterosis in wide crosses in wheat. Sci Adv. 2020;6(24):eaay4897. doi: 10.1126/sciadv.aay4897.

35.	Zhao Y, Thorwarth P, Jiang Y, Philipp N, Schulthess AW, Gils M, et al. Unlocking big data doubled the accuracy in predicting the grain yield in hybrid wheat. Sci Adv. 2021;7(24):eabf9106. doi: 10.1126/sciadv.abf9106.

36.	Kante M, Rattunde F, Nébié B, Sissoko I, Diallo B, Diallo A, et al. Sorghum Hybrids for Low-Input Farming Systems in West Africa: Quantitative Genetic Parameters to Guide Hybrid Breeding. Crop Sci. 2019;59(6):2544-61. doi: <https://doi.org/10.2135/cropsci2019.03.0172>.

37.	Poorter H, Fiorani F, Pieruschka R, Wojciechowski T, van der Putten WH, Kleyer M, et al. Pampered inside, pestered outside? Differences and similarities between plants growing in controlled conditions and in the field. New Phytol. 2016;212(4):838-55. doi: <https://doi.org/10.1111/nph.14243>.

38.	Tang R-J, Luan S. Regulation of calcium and magnesium homeostasis in plants: from transporters to signaling network. Curr Opin Plant Biol. 2017;39:97-105. doi: <https://doi.org/10.1016/j.pbi.2017.06.009>.

39.	Bradshaw Jr HD. Mutations in CAX1 produce phenotypes characteristic of plants tolerant to serpentine soils. New Phytol. 2005;167(1):81-8. doi: <https://doi.org/10.1111/j.1469-8137.2005.01408.x>.

40.	Lenz H, Dombinov V, Dreistein J, Reinhard MR, Gebert M, Knoop V. Magnesium Deficiency Phenotypes Upon Multiple Knockout of Arabidopsis thaliana MRS2 Clade B Genes Can be Ameliorated by Concomitantly Reduced Calcium Supply. Plant Cell Physiol. 2013;54(7):1118-31. doi: 10.1093/pcp/pct062.

41.	Thor K. Calcium—Nutrient and Messenger. Front Plant Sci. 2019;Volume 10 - 2019.

42.	Ahmed N, Zhang B, Bozdar B, Chachar S, Rai M, Li J, et al. The power of magnesium: unlocking the potential for increased yield, quality, and stress tolerance of horticultural crops. Front Plant Sci. 2023;Volume 14 - 2023. doi: 10.3389/fpls.2023.1285512.

43.	Tang R-J, Zhao F-G, Garcia VJ, Kleist TJ, Yang L, Zhang H-X, et al. Tonoplast CBL–CIPK calcium signaling network regulates magnesium homeostasis in Arabidopsis. Proceedings of the National Academy of Sciences. 2015;112(10):3134-9. doi: 10.1073/pnas.1420944112.

44.	Huey SL, Islam S, Mehta NH, Konieczynski EM, Friesen VM, Krisher JT, et al. Review of the facilitators and barriers to adoption of biofortified foods and food products. Nutr Res Rev. 2025;38(1):371-92. Epub 2024/10/08. doi: 10.1017/S0954422424000258.

45.	Ahoudou I, Sogbohossou DEO, Fassinou Hotegni VN, Fanou-Fogny N, Moumouni-Moussa I, Achigan-Dako EG. A systematic review on the factors influencing adoption and consumption of orange-fleshed sweetpotato in sub-Saharan Africa. Frontiers in Sustainable Food Systems. 2025;Volume 9 - 2025.

46.	Achigan-Dako EG, Sogbohossou DEO, Houdegbe CA, Salaou MA, Sohindji FS, Blalogoe J, et al. Ten Years of Gynandropsis gynandra Research for Improvement of Nutrient-Rich Leaf Consumption: Lessons Learnt and Way Forwards. Annual Plant Reviews online2021. p. 767-812.
